# Communicating about overdiagnosis: Learning from community focus groups on osteoporosis

**DOI:** 10.1371/journal.pone.0170142

**Published:** 2017-02-03

**Authors:** Ray Moynihan, Rebecca Sims, Jolyn Hersch, Rae Thomas, Paul Glasziou, Kirsten McCaffery

**Affiliations:** 1 Centre for Research in Evidence-Based Practice, Bond University, Gold Coast, Queensland, Australia; 2 School of Public Health, The University of Sydney, Sydney, New South Wales, Australia; Van Andel Institute, UNITED STATES

## Abstract

**Background:**

Overdiagnosis is considered a risk associated with the diagnosis of osteoporosis–as many people diagnosed won’t experience harm from the condition. As yet there’s little evidence on community understanding of overdiagnosis outside cancer- where it is an established risk of some screening programs–or effective ways to communicate about it. We examined community understanding around overdiagnosis of osteoporosis, to optimise communication strategies about this problem.

**Methods and findings:**

Using a qualitative design we recruited a community sample of women, 50–80 years, from the Gold Coast community around Bond University, Australia, using random digit dialing, and conducted 5 focus groups with 41 women. A discussion guide and 4-part presentation were developed and piloted, with independent review from a consumer and clinical experts. Initial discussion had 4 segments: osteoporosis; bone density vs. other risk factors; medication; and overdiagnosis. The second half included the 4 short presentations and discussions on each. Analysis used Framework Analysis method. Initially participants described osteoporosis as bone degeneration causing some fear, demonstrated imprecise understanding of overdiagnosis, had a view osteoporosis couldn’t be overdiagnosed as bone scans provided “clear cut” results, expressed belief in early diagnosis, and interest in prevention strategies enabling control. Following presentations, participants expressed some understanding of overdiagnosis, preference for describing osteoporosis as a “risk factor” not “disease”, concern about a poor risk-benefit ratio for medications, and surprise and unease the definition of osteoporosis decided bone density of young women was “normal”, without age adjustment. Limitations include English-speaking backgrounds of the sample and complex materials.

**Conclusions:**

Our findings suggest a gap between community expectations and how experts sometimes arbitrarily set low diagnostic thresholds which label those at risk as “diseased”. Optimal communication about overdiagnosis could build on community scepticism about treatments, encouraging weighing up benefits and harms of tests and diagnoses, and framing this information as positively adding to knowledge.

## Introduction

Overdiagnosis is increasingly recognised as a significant source of harm and waste within healthcare systems, and there are growing global initiatives to combat it [1]. Overdiagnosis happens when people are given a diagnosis for a disease that will never harm them. It can lead to unnecessary tests and treatments that may carry harms to the patient and may also divert health resources away from treating and preventing genuine illness. The problem has technological, commercial and cultural causes, and is commonly associated with cancers diagnosed as a result of screening healthy people [2]. An inquiry in the United Kingdom has estimated, for instance, that perhaps 1 in 5 cancers diagnosed via mammography screening may never cause women harm [3].

Osteoporosis–low bone mineral density–has the potential to be overdiagnosed, since according to its definition, many otherwise healthy people are labelled with a “disease” because they are at risk for future fracture [4]. The modern definition of osteoporosis comes from a study group which included representation from the World Health Organisation, and had funding from pharmaceutical companies [5]. In 1994, that group decided to define “normal” bones as those of young adult women, and proposed the threshold for osteoporosis as bone mineral density 2.5 standard deviations below the young adult mean. At the time, the group noted: their decision automatically classified around 30% of all post-menopausal women as having a “disease” called osteoporosis; the cut-off value they used was “somewhat arbitrary”; and bone density values could also be seen as a “risk factor” for future fracture [5]. Prefiguring contemporary debates about overdiagnosis the 1994 group also asserted “the disease may or may not give rise to symptoms” [5].

While a diagnosis of osteoporosis can motivate preventive measures, it also carries downsides: bringing fear that may limit activities to avoid falling [6], and leading women to see themselves as weak [7]. Welch, Schwartz and Woloshin have suggested many women diagnosed with osteoporosis can be considered overdiagnosed because they would never experience a fracture [2]. Järvinen and colleagues similarly argue osteoporosis is overdiagnosed, putting many women who are diagnosed at risk of experiencing more harm than good [4]. They point to small absolute reductions in fracture risks from widely used medications which mean large numbers of healthy people are treated without benefit, but with the chance of potentially serious, though rare, physical harms. Critics of this view reject concern about overdiagnosis and respond that strengthening the skeleton with medications can meaningfully reduce risk, particularly for people at high risk, and should be part of management strategies along with lifestyle modifications and falls prevention [8].

A recent national community survey about overdiagnosis in Australia found few people had been informed about the topic and a large majority wanted more information on it [9]. To date, studies on communicating with people about overdiagnosis have focussed almost solely on cancer, where, for instance, initial qualitative focus group research [10] has informed a randomised trial, ultimately finding decision-aids could enhance women’s informed choices around mammography [11]. In an era of shared–decision making, there is an urgent need to inform people about the risks of overdiagnosis associated with non-cancer conditions, including osteoporosis.

We aimed to explore community awareness of the overdiagnosis of osteoporosis and related controversies surrounding the condition, including the definition, whether it is best understood as a “disease” or a “risk factor”, and the perceived value of the most common medications–as well as responses to potentially new information about these issues Our findings will inform current global efforts to better communicate about osteoporosis and about overdiagnosis generally.

## Methods

### Design

We used focus groups to examine community thinking about the complex, and often counter-intuitive issues around overdiagnosis, exploring both underlying awareness and responses to new information about the problem. While osteoporosis is sometimes diagnosed in men, due to different changes in bone density between men and women as they age, the 1994 definition axiomatically labels many more women, so we chose to recruit women only. The focus groups offered an informal setting in which to hear women’s unprompted understanding of overdiagnosis and their responses to new information. Women were encouraged to interact with each other, and were told there were no right answers. While structured, the focus group offered the opportunity for discussion to move in unanticipated directions. Ethics approval was granted by the Bond University Human Research Ethics Committee (Application# 0000015292) and all participants gave informed consent in writing.

### Recruitment and participants

A university-based research organisation, the Population Research Laboratory, was engaged to recruit a community sample of 65 women aged 50–80 years, fluent in English and without a diagnosis of osteoporosis, from the Gold Coast community in Queensland, Australia, chosen due to its proximity to Bond University where the focus groups were conducted. Recruiters used sample provider Sampleworx to randomly generate landline telephone numbers within Gold Coast post-codes. Purposive sampling was used to recruit roughly equal numbers of women across the 50s, 60s and 70s age groups, and across educational backgrounds. Following recruitment the research company provided names and contact details to the research team, and one author, RS, sent women an Explanatory Statement (Supporting Information file S1 Text) and facilitated attendance.

To assess unprompted awareness of overdiagnosis during the initial focus group discussion, the Explanatory Statement did not mention the term overdiagnosis but stated the study was designed “to discover more about community understanding of the diagnosis of osteoporosis” and was “part of broader research about the risks and benefits of medical diagnoses.” Each group was approximately 2 hours 15 minutes and held in an accessible meeting room. All participants were paid A$100, offered refreshments and assistance with travel costs. Focus groups were audio recorded and professionally transcribed. Reasons for non-participation of those recruited were recorded.

### The research team

Following the COREQ checklist for reporting qualitative research [12] we report we have expertise across clinical medicine, clinical epidemiology, communication, psychology, and overdiagnosis. RM has previously written about osteoporosis and medicalization [13], and completed his PhD on overdiagnosis in 2015. RS is a research assistant with a psychology degree and no background in overdiagnosis. RT is a psychologist and senior researcher with an interest in overdiagnosis. JH is a post-doctoral researcher specialising in communicating about overdiagnosis. PG is an academic general practitioner and clinical epidemiologist with specialist interests in evidence-based practice and overdiagnosis. KM is a psychologist and senior qualitative researcher with an interest in overdiagnosis and communication science.

### Focus group materials

The focus group structure, moderator guide and presentation material were iteratively developed, with input from all team members, two independent external clinical reviewers with expertise in osteoporosis, and one independent consumer representative acknowledged below. To test materials and processes, we ran two pilot focus groups with convenience samples of university administration staff in the target age and gender, and made several revisions to format and presentation.

The final format included approximately one hour of introduction and initial group discussion, guided by structured, open-ended questions across four topic areas. (see Box 1 and Supporting Information files S2 and S3 Texts). The segments were: 1) What is Osteoporosis?; 2) Relative importance of bone mineral density compared to other risk factors; 3) Common medications; and, 4) Overdiagnosis. The second hour comprised of four short pre-recorded videoed powerpoint presentations addressing these topics (up to six minutes), with each presentation followed by participant discussion. (Supporting Information files S3 and S4 Texts). The content for each short presentation was drawn from a range of scientific literature, cited in Box 1, and powerpoint slides were presented in the short video segments by co-investigator and internationally recognised expert in evidence based practice Professor Paul Glasziou, PG.

**Box 1. Structure of 2-hour focus group session**

*First hour*: *Women’s initial understanding and perceptions about osteoporosis and overdiagnosis of this condition*:

Q: What is osteoporosis?

Q: Apart from bone density are there other things increasing fracture risk?

Q: How well do common medications for osteoporosis work?

Q: Among people diagnosed, how many will never have a fracture?

*Second hour*: *Four brief expert presentations and women’s responses to new information about osteoporosis and overdiagnosis*

1. Presentation about 1994 definition of osteoporosis and debate about whether best described as “risk factor” or “disease” [4,5,13,15–17]
  Q: How do you feel about describing osteoporosis as a “risk factor” or a “disease”?
2. Presentation about bone mineral density, BMD as one of many risk factors, and mention of absolute risk calculators [4,15,18,19]
  Q: How do you feel about the importance of BMD compared to other risk factors?
3. Presentations about medications, including 7 examples drawn from the Cochrane review [20] with text and pictograms about benefits of alendronate, for both primary and secondary prevention, including impacts on hip, spinal and non-spinal fractures, and text about potential harms [21–24]
  Q: How do you feel about benefits? How do you feel about potential harms?
4. Presentation about overdiagnosis, including brief explanation of overdiagnosis, and discussion of possibility of women diagnosed with osteoporosis being overdiagnosed [2,4,18]
  Q: How do you feel about the possibility some women with osteoporosis might be overdiagnosed?

*Note*: *Full text and details in Supporting Information files* S2, S3 and S4 Texts.

RT was moderator for all focus groups, with technical assistance from RS. PG was also available in person or via phone consultation at the end of each group to answer questions. Using short written questionnaires, participants provided demographic information at the start of each group, and answered questions assessing comprehension of overdiagnosis at the close.

### Data capture, coding and analysis

The theoretical framework was drawn from the phenomenology approach, seeking to understand the “constructs, concepts or ideas people use in everyday life to make sense of their world” [14]. The method of thematic analysis was based on “Framework Analysis” as described by Ritchie and colleagues [14].

During preliminary thematic analysis, two authors, RM and RS, independently reviewed transcripts and independently developed an initial list of topics/themes arising from the transcripts, analysing: i) responses to each question; and, ii) overall responses across the transcripts. Following discussion and feedback from co-investigators (RT, who was present during all focus groups, and JH, KM who had also reviewed some transcripts), a comprehensive list of themes and sub-themes was developed. Using this list, RM and RS independently indexed and coded the transcript of focus group 5. Following discussion of agreements and discrepancies in indexing and coding, small changes were made to the definition of several sub-themes to achieve increased clarity. RM and RS then each coded two of the remaining four transcripts. The “framework analysis” method involved using Excel spreadsheets for indexing and summarising data. Following indexing and coding, RM and RS developed a list of potential findings and shared the framework with co-investigators (KM, RT, JH) for discussion and elaboration. JH and RT independently assessed participant answers to written comprehension questions, and discrepancies were adjudicated by RM.

## Results

Forty-one women aged 50–80 from a range of educational backgrounds participated in five focus groups, each containing between seven and nine women, with participant characteristics in Table 1. Women were almost entirely from English-speaking backgrounds, a phenomenon reflecting the demographics of the Gold Coast community [25]. Of the 65 potential participants recruited initially, 24 did not participate in a focus group, with reasons including: unable to be contacted (n = 8); work commitments (n = 5); unable to attend scheduled times (n = 3); and, no explanation (n = 8). Despite the recruitment organisation excluding women who reported a diagnosis of osteoporosis during initial recruitment, (in response to a question about whether they had ever had a diagnosis of osteoporosis), in written demographic questions after the focus groups had commenced five women reported having a current and/or a previous diagnosis, though we found no meaningful differences between the comments of these participants and the rest of the group.

**Table 1:** Characteristics of participants (n = 41).

Age	50–59	60–69	70–80
	12	16	13
Age when finished full time education	Under 15	15–18	18+
	11	11	19
	Yes	No	Don’t know
Current diagnosis of osteoporosis	3	34	4
Previous diagnosis of osteoporosis	4	35	2
Diagnosis of osteopenia(a)	5	29	7
Tested for osteoporosis	16	23	2
Previous fracture	21	20	0

All information is self-reported. Two women reported having a current and former diagnosis of osteoporosis, and three women having one or the other. (a) Osteopenia was defined in 1994 as BMD more than 1 SD below the young adult mean, but less than 2.5 SD below. [5]

Following the thematic analysis, findings were organised into a brief summary of participants’ initial underlying perceptions and then five domains within which we analysed responses to potentially new information in the presentation: “Risk factor” versus “disease”; The dilemma of diagnosis; Medications and prevention; Overdiagnosis; and Questioning the definition.

### Participants’ initial perceptions of osteoporosis

Women consistently described osteoporosis as bones weakening, degenerating, thinning and breaking down, which for some, was a cause of a level of concern or fear. A common theme across all groups was a strong interest in strategies they perceived to reduce the risk of falling and fractures, including use of calcium and Vitamin D supplements, and changes to diet, exercise, lifestyle, and modifications to home environments. Benefits of a diagnosis, and early diagnosis, were seen as motivating such changes, while downsides included increased worry and potential de-motivation to make changes. There was a low awareness of medications for osteoporosis, though comments displayed a general sensitivity to medication side effects.

### “Risk factor” versus “disease”: Preference for risk factor

Following the first expert presentation, which described the debate among doctors about whether osteoporosis was best described as a “risk factor” or a “disease”, many participants expressed a preference for describing it as a “risk factor”. They expressed a range of reasons for their preferences for “risk factor”, sometimes related to the sense of being able to take more personal control. In addition, “risk factor” suggests some women won’t experience harm, whereas “disease” implies automatic illness. Women felt the use of “disease” could “brand” people, and connote something out of individual control.

“This is a risk factor which there are certain things that can lead to us being at risk of fractures, but it's not a pathogen that's actually causing us to have that fracture.”

(aged 54)

“Disease makes me think of a really bad illness… this isn't really what I would class as a disease, it's something that you can actually help yourself with and do things about.”

(aged 66)

A few people were not entirely happy with either, feeling “risk factor” may not be a strong enough term to motivate healthy lifestyle changes.

“I hear the point about … people being labelled [with a disease] as perhaps being negative, but that might stimulate them to change some behaviours that reduces their risk of fractures … But are “risk factors” maybe not strong enough to make people do—have those lifestyle changes that is going to reduce their risk?”

(aged 53)

### The dilemma of diagnosis: Awareness of downsides, belief in early diagnosis

Participants articulated clear views about the potential benefits and, to a lesser extent, the downsides of receiving a diagnosis of osteoporosis. A diagnosis was seen as giving information or adding to knowledge, which could motivate change and enable people to take control and implement prevention strategies.

“I think knowledge is power, so if you're informed, you can do something about that”

(aged 56)

However, a diagnosis was also seen by some to potentially bring fear which might demotivate.

“I think you might have a fear, it might give you a fear and also prevent you from doing things because of that fear.”

(aged 79)

Unprompted expressions of belief in the value of *early* diagnosis were a feature of discussions before presentations, and were still present, though to a lesser extent, after the presentations.

“I think that the sooner they are diagnosed, the better the chances…a person will have of looking after themselves.”

(aged 68)

“I'm thinking that if it's early enough… the progression of the disease could be halted.”

(aged 62)

The idea that early diagnosis could potentially save money for the health system was also expressed as a potential benefit:

“…you do save the hospital system a lot if you find out early.”

(aged 69)

“…that will lead to less costs for the community and the governments…”

(aged 54)

### Medications and prevention: Underwhelmed by drugs, interest in other strategies

Initially there was generally low awareness of medications for osteoporosis, although a few women saw them as beneficial, and one was aware of potential harms. After the initial discussion about the meaning of overdiagnosis, but before any presentations, a number of participants expressed general concerns about potential side effects of medications and overprescribing, and about the influence of pharmaceutical companies over doctors.

“…the medical industry is very interesting, it is a business after all and they're there to make money, they're there to sell product…”

(aged 72)

“…people are getting a little bit suspicious of all these medicines and medication and these companies, drug companies that make huge marketing on these new pills that are popping up.”

(aged 68)

A dominant theme across all groups was strong interest in what they perceived as the value of non-medical strategies to reduce the risks of falls and fractures, including lifestyle changes, diet, exercise, calcium, vitamin D, and a range of ways in which people tried to take control of their health and minimise their chance of falling–particularly as they got older–eg removing slippery mats, using shoes with more grip.

“I'm nearly 69, I'm old and frail when I go down the steps. I am always thinking prevention because I'm not about to take the risk of falling down a step or tripping over…”

(aged 68)

During the presentation about medications for osteoporosis, women were shown pictograms and text about the absolute benefits and potential harms of the most common osteoporosis medication, alendronate. (See Supporting Information file S3 Text) In the participant discussion following this presentation, information about risks and benefits were weighed up, with a general perception of a poor risk/benefit ratio. This perception was nonetheless accompanied by openness to the value of medications, particularly for those who would benefit most.

“…I probably wouldn't take it. I'd just go with the risk…If there was a massive decrease in people [having a fracture], then I might sort of think about putting up with a few of the side effects, but I would not go on it.”

(aged 50)

“…If the benefits is only small, like in terms of the number of fractures reduced, maybe it doesn't sound all that good. But if you're the one who's had that fracture reduced, then it's worth it to you.[general agreement]

(aged 57)

### Overdiagnosis: Complexities in communicating counter-intuitive concept

When asked initially what overdiagnosis in general meant, there was overall an imprecise understanding compared to the commonly accepted medical definition [2], with participants often associating it with overtreatment/overtesting:

“I think it's overdiagnosing with too many tests and when there's something perhaps minor and the doctor would send you for this test, this test, this test, when really it's probably not necessary…”

(aged 66)

“I guess there's a danger that you might get treatment that you don’t really need and that treatment could cause problems too, plus the anxiety of having to go through it all.”

(aged 68)

In response to a question, during the initial discussion, about whether osteoporosis could be overdiagnosed, there were clearly expressed views osteoporosis could not be overdiagnosed because bone scans would provide a “clear cut”, “black and white” diagnosis. There was no awareness among participants of any controversy or uncertainty around the definition or diagnostic thresholds for osteoporosis, or understanding that people could be diagnosed but never have symptoms.

“…it's a black and white situation, you do have it or you don't.”

[general agreement] (aged 72)

“I would say if the diagnostic tool is a bone scan, then they can't overdiagnose that because the reading of it is quite clear cut.”

(aged 53)

The only disagreement with this view in any group came from one woman who had worked in breast cancer screening.

“… when I was … working in a breast screen department, there was differing opinions about what was a breast cancer according to the images. So even within the so-called experts, there was different degrees of what was an issue and what wasn't.”

(aged 58)

### Overdiagnosis in osteoporosis: Changing perceptions after new information

Following the presentation about overdiagnosis and osteoporosis, more participants demonstrated a comprehension of the problem, expressing concern some people might be diagnosed but never experience symptoms.

“… you can have osteoporosis or I think you can, never have a fracture and maybe it doesn't really affect you if you've got it.”

(aged 57)

“I think it is dishonest to tell someone they've got something wrong with them that they don't have wrong with them, then prescribe something for them that they don't need and cause the anxiety and the worry of it”

(aged 71)

### Questioning the definition of osteoporosis: Unease over young women’s bones defined as “normal”

The first short presentation provided to participants included information from the 1994 definition, stating that it had defined “normal” bones as those of young women [5]. When asked about this definition, participants expressed unease that “normal” was not adjusted for age, a sense that the definition was “strange” and nonsensical, and a view from some that it should be reformed. It was implicit in women’s responses they had assumed the definition of “normal” did take account of a person’s age.

“I would suggest that maybe we look at the bones of a wide range of 80-year-old people and then define healthy bones from an 80-year-old based on 80 years of standing upright, walking, eating, living…”

(aged 54)

“…if you're 50, you would think that you would be benchmarked against 50. I mean otherwise you're going to get huge overdiagnosis”

(aged 66)

### Understanding overdiagnosis: Written responses

Independent assessment (by two authors RT and JH) of anonymous written responses to comprehension questions regarding overdiagnosis at the close of the focus groups achieved strong agreement. Roughly a fifth of participants were deemed to have correctly described what overdiagnosis means–as compared to the definition offered during the presentation–with a further half offering a partially correct answer, suggesting a nascent understanding that people can be diagnosed with a disease that would not cause them harm.

“You may never get any pain or problems”

“Diagnosing something that will have no impact on your health and taking a preventative medication when not needed”

Many participants’ responses that did not show a comprehension of overdiagnosis specifically, did however demonstrate an awareness of overtreatment: treatment was mentioned in roughly half of all written explanations of overdiagnosis.

## Discussion

### Principal findings

The focus groups on osteoporosis and overdiagnosis with this community sample of women produced a number of key findings, both prior to and following brief informative presentations. Prior to presentations, group discussion revealed almost no comprehension of the concept of overdiagnosis in general, and strong views on the impossibility of any unnecessary diagnosis of osteoporosis due to the “clear-cut” nature of diagnostic test results. Participants also expressed a belief in the value of diagnostic labels and early diagnosis, with these thought to have the potential to motivate lifestyle changes in some individuals, and demotivate others. Initial discussions also revealed a general concern about medication side effects and the influence of the pharmaceutical industry, and a strong interest in a range of non-medical prevention strategies.

Following brief presentations, many participants expressed: a preference for describing osteoporosis as a “risk factor” rather than “disease”; concern about poor benefit/risk ratios of osteoporosis medication; a nascent, though partial, understanding that people could be diagnosed with a disease that would not cause harm; and, surprise and unease about the clinical definition of “normal” bones being those of a young woman, without adjustment for age. A key element abstracted from the focus group discussions was a desire by women to take control over their health, using knowledge arising from a diagnosis, and valuing information allowing them to weigh up the benefits and risks of medications and other strategies to prevent falls and fractures.

### Limitations and strengths

We deliberately recruited women only, and reflecting the demographics of the Gold Coast, the sample was predominantly from English-speaking backgrounds. While there was a range of participant ages and educational backgrounds, as is usual with qualitative work our sample was not designed to be representative. The expert presentations were short, restricting the time available to explain complex and counterintuitive concepts which are demonstrably difficult to communicate [26]–a view confirmed by the relatively small number who could accurately describe overdiagnosis at the completion of the session; however measures were taken to ensure presentations were evidence-based and comprehensible, including using independent clinicians and a consumer to review the presentations and running pilot focus groups. A potential for bias is present through several authors possessing strong interest in overdiagnosis, however this was minimised through: independent review of the presentations; no discussion of content with participants before the focus group, and the moderator offering no comment on medical content during the focus group; and thematic analysis conducted independently by two coders, with the wider team developing findings.

To our knowledge, this study is the first to qualitatively examine community views about the increasingly recognised health challenge of overdiagnosis, within a non-cancer condition. Similarly it is the first to seek community views on how expanded definitions of “disease” can lead to overdiagnosis, in contrast to the problem of overdetection arising via cancer screening. In order to reduce volunteer bias, we used an independent, not-for-profit research company to recruit a sample from the community using random digit dialling. Our findings have important implications and will help build the evidence-base from which to develop and evaluate optimal communication strategies about overdiagnosis, particularly where it occurs as a result of expanding disease definitions and lowering diagnostic thresholds.

### Implications for communicating about overdiagnosis

Prior to presentations many participants felt osteoporosis could not be overdiagnosed because bone scans were considered a definitive result, suggesting a belief in the truth of numerical test results, without any awareness of the uncertainty surrounding diagnostic processes or thresholds, or disease definitions. Importantly, the only participant who disagreed had direct experience of working in breast cancer screening, where overdiagnosis is an established risk and she’d witnessed debate about what constituted cancer. In contrast to belief in tests, participants were cautious about medications, explicitly weighing up risks and benefits. These findings align with previous qualitative findings of “great faith” in bone scans [7], general enthusiasm for tests, and wariness towards medications [27]. Communication about overdiagnosis can build on this healthy questioning of treatments, and extend it to diagnostic tests and subsequent labels, which also carry benefits and harms, including the risk of overdiagnosis.

Participants expressed strong belief in the benefit of early diagnosis which could bring knowledge and in turn could enable more control over future health, a belief only lessened slightly following presentations. Generally absent from participant understanding was awareness that “early diagnosis” can be a double-edged sword, with potential benefits and potential harms, including being labelled with a “disease” that will never cause symptoms. In line with previous qualitative findings that citizens favour “active, engaged, information-seeking roles” [27], framing information about overdiagnosis and the downsides of “early diagnosis” as positively adding to people’s knowledge about their health may optimise communication about these issues.

Participants often associated overdiagnosis, both in discussion and in their written answers, with problems of overtreatment, suggesting communication strategies should be sensitive to and build on this fact, particularly at a time when the definition of overdiagnosis is evolving [28]. While “overdiagnosis” and “overtreatment” are rightly considered separately for the purposes of research, in much of the community the terms appear to be considered closely integrated.

Our finding that some participants expressed surprise and unease that a disease definition would define the bones of the young as “normal”, may also be relevant to other conditions where overdiagnosis is a risk. With “chronic kidney disease”, similarly to osteoporosis, “normal” kidney function is based on young individuals and disease thresholds are unadjusted for age, creating conditions for the medicalisation of aging and overdiagnosis [29], and sparking concern among general practice [30]. Our findings have identified a potential gap between community expectations and the way some diseases are constructed and thresholds set, unadjusted for age and axiomatically causing overdiagnosis. They also help inform the reform of disease definition processes, currently underway within the Guidelines International Network [31] which is designed to ensure expert panels consider both benefits and potential harms before setting new diagnostic thresholds.

Some participants thought reduced bone density may be better described as a “risk factor” for future fracture, rather than a “disease” called osteoporosis. In recent years there’s been a move towards overall fracture risk assessment, with models including bone density as one of many factors, and even the original definition acknowledging it could be seen as a “risk factor” [5]. Despite this, however, much of the contemporary promotional material continues to describe osteoporosis directly as a “disease” [32]. If risk-based conditions like osteoporosis are best described and understood as “risk factors” rather than “diseases”, the question of whether they can be overdiagnosed becomes more complex. “Risk factor” inherently suggests no harm may come to some of those labelled, whereas “disease” implies illness. Responses to the challenge of overdiagnosis of risk-based conditions such as hypertension or high cholesterol, may require a different approach than that applied to diseases such as cancer.

## Conclusions

Our aim in this study was not to educate participants, but rather to learn more about how to communicate about overdiagnosis for a non-cancer condition for which the definition automatically labels many people who won’t experience harm as “diseased”. Osteoporosis offered a strong example, given professional awareness, from its inception [5], that a proportion of those diagnosed with this “disease” would not have symptoms, as well as more recent explicit concern about its overdiagnosis [2,4] and overtreatment with osteoporosis medications [33,34]. The findings suggest a need to better communicate about the arbitrary and controversial nature of some disease thresholds, as well as underscoring the need to reform the way they are set. It is also important to convey that early diagnosis is a double-edged sword because tests and diagnoses, like treatments, can have serious downstream harms. Ultimately, information about overdiagnosis might be framed optimally as positive knowledge that can help people take control of their health. (Box 2) Further research in this field will develop and evaluate materials and approaches to effectively inform people about overdiagnosis.

**Box 2. Implications for communicating about overdiagnosis**

1. Build on community healthy scepticism towards treatments and extend to diagnostic tests
2. Communicate idea “Early diagnosis is a double-edged sword” as important new knowledge
3. Build on the community’s association of “overdiagnosis” with overtreatment
4. Build on community antipathy to disease definitions which axiomatically overdiagnose
5. Risk-based conditions require different communication approaches to other diseases

## Supporting information

S1 Text This Explanatory Statement was given to focus group participants. (PDF)

S2 Text This was the moderator guide used to guide the moderator/facilitator. (PDF)

S3 Text This was the powerpoint presentation shown to participants, which included the discussion questions for the first hour and for the second hour, the video portions were embedded within it. (PPTX)

S4 Text This is the transcript of the audio for the video presentations given to participants. (PDF)
