# Supplementary material for: Communicating about overdiagnosis: Learning from community focus groups on osteoporosis
Source: PLoS One. 2017 Feb 3;12(2):e0170142. doi: 10.1371/journal.pone.0170142 (PMC5291414; doi:10.1371/journal.pone.0170142)
Supplement: S1 Text — (PDF) [file pone.0170142.s001.pdf]

Osteoporosis Focus Group Study

Explanatory Statement **BUHREC Protocol Number:** 0000015292

Dear potential study participant,

Thank you for agreeing to be contacted by the team of researchers from Bond University who are conducting a study on women's views about osteoporosis. This letter tells you what the study involves and how you might be able to help.

About the study

You are invited to attend a group discussion with a small group of women, to discover more about community understanding of the diagnosis of osteoporosis. This study is part of broader research about the risks and benefits of medical diagnoses. The results of the focus group study will help researchers find better ways to communicate about the risks and benefits of medical tests and treatments.

The study has been approved by the Bond University Human Research Ethics Committee and participation is completely voluntary.

If you agree to participate in a discussion group, your contributions will remain anonymous in any subsequent publication of results. The group discussions, also known as focus groups, will run for about 2 hours and will be recorded and transcribed. They will be led by a moderator, and will include some information that may be new to you, and you will be asked your reactions to this information.

All results will be strictly confidential and only study researchers will have access to them. Individual participants will not be identifiable in any scientific articles or presentations about the study.

The discussions will be held at the Robina Campus of Bond University. Detailed instructions on how to get there will be provided to you, and some assistance can be offered with transport, if that is needed. Participants will be paid $100 for their participation at the end of the session, and light catering will be available.

What to do next

A researcher will contact you shortly, to answer any questions you have and to invite you to take part in one of the group discussions we are planning, at a time that is convenient to you.

If you do decide to participate, you can withdraw at any time without having to give any reason for doing so. If you do take part in the discussion, and wish to withdraw after the session has commenced, you are free to do so. This will not affect your compensation for volunteering your time, but your comments will remain part of the recorded group discussion.

If you have any questions about the research or would like to be informed about the summary of research findings, please contact me, Dr Ray Moynihan, the Principal Investigator.

Yours Sincerely,

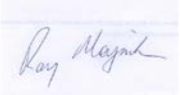

Dr Ray Moynihan
Centre for Research in Evidence-Based Practice,
Faculty of Health Sciences and Medicine,
Bond University,
Gold Coast, Queensland,
Australia, 4229,
Phone: 07 5595 4482;
Email: raymoynihan@bond.edu.au

This study has been approved by the Bond University Human Research Ethics Committee. If you have any concerns about the manner in which this research is being conducted, please contact the committee, c/o Bond University Office of Research Services. Bond University, Gold Coast, 4229 Phone: +61 7 5595 4194 Fax: +61 7 5595 1120 Email: buhrec@bond.edu.au
