# Supplementary material for: Communicating about overdiagnosis: Learning from community focus groups on osteoporosis
Source: PLoS One. 2017 Feb 3;12(2):e0170142. doi: 10.1371/journal.pone.0170142 (PMC5291414; doi:10.1371/journal.pone.0170142)
Supplement: S2 Text — (PDF) [file pone.0170142.s002.pdf]

Community Attitudes to Osteoporosis

FOCUS GROUP MODERATOR GUIDE

November 5, 2015

SUMMARY

0:00 – 1:00*	[A]	introduction, consent, questionnaire 1, discussion questions
1:00 – 2:00	[B]	presentation in 4 parts, discussion in 4 parts, questions
2:00 – 2:15	[C]	final discussion and conclusion

Participants who arrive early should be invited to have a cup of tea/coffee and complete the Consent Form.

*Rough guide to timings

[Part A]

SLIDE 1 INTRODUCTION

- *Welcome participants and brief introductions*
- *Request participants to switch off mobile phones or put them on 'silent'.*

THE STUDY

This study is part of a project with the overall aim of helping to improve communication about the risks and benefits of medical tests and treatments. The purpose of this study and the session today is to find out about your knowledge and feelings about **osteoporosis**.

AIMS OF SESSION

Following this brief introduction, we will ask those who haven't already to fill out the consent form and answer a few brief questions.

It's important to let you know before we start that we are not here to try and influence you or convince you to do one thing or another; we're here to find out what you think and feel about the subject matter.

Focus Groups are a chance for researchers to listen to what the community is saying – you may learn some new things – but the primary purpose is for us to learn from you – so this is not an educational session.

AUDIO RECORDING & CONFIDENTIALITY

As we have mentioned in the information you received, we would like to make an audio recording of this session, just to make sure we can accurately summarize the discussion. And as we've made clear in the Explanatory Statement, everything that's said or written today will be kept strictly confidential. When the study results are reported, it will not be possible to identify any individual participants.

- *Explain that this focus group cannot give medical advice but the presenter will answer questions about the presentation towards the end of today's session, and you can follow up any outstanding questions later.*

- *Explain set-up of session (food, bathroom, time frame).*

- *Administer **consent form** if not already done*

SLIDE 2 Answer a few short demographic questions.

SLIDE 3 STRUCTURE OF TODAY

For the first part of the session we are going ask a series of questions about osteoporosis, for the group to discuss. Next we're going to give you some brief information with the help of a PowerPoint presentation, and we'll ask you to talk about your reactions and views towards the information.

During the discussions we would like to hear everyone's opinions so that we can understand a wide range of views. We are not looking for a consensus about anything so it is completely fine for you to disagree with each other, but obviously please try not to criticise others. While I'm the facilitator, we'd like you to talk to each other.

It doesn't matter what you know about osteoporosis, we just want to hear your comments.

Are there any questions about the process?

- *With participants' consent,* ***start recording****.*
  Request that women speak up and avoid speaking at the same time as others.

- *Ask women to introduce themselves briefly.*

START RECORDING

DISCUSSION ITEMS [Part A] [moderator]

1. **What is osteoporosis?**
   What do you know and feel about osteoporosis?
   How does osteoporosis get diagnosed?
   What are the benefits of diagnosing it?
   Are there any downsides to being diagnosed with osteoporosis?
   Is it a disease?

2. **Apart from the density of your bones, are there other things that might increase the chances that you will have a fracture in the future?**
   (if needed – "a fracture is a broken bone – for example a hip fracture")
   Are there other factors that increase the risk of having a fracture?
   What are they?
   How important is bone mineral density in relation to all the other factors – in terms of increasing your risk of having a fracture?

3. **How well do common medications for osteoporosis work?**
   (if there is need to be specific, explain the most common medications are bisphosphonates, one of which is alendronate, or Fosamax)
   What are the benefits of using medications to treat osteoporosis?
   Can you describe the benefits using numbers? (could lead in gently – eg – if someone says 'prevents fractures' – could ask how many have a fracture prevented – or does everyone have a fracture prevented, or just some)
   Can you estimate, using numbers, the chances of a woman in her 50s having a hip fracture?
   What are the harms or downsides of treating osteoporosis with medications?

4. **Among people who are diagnosed with osteoporosis, how many of them will never have a fracture?**
   Almost all? About half? Very few? None
   Have you ever heard about the problem of overdiagnosis?
   (If people ask what do you mean, say we will explain later in the focus group)
   What do you know about overdiagnosis?
   Can someone who is diagnosed with osteoporosis be overdiagnosed?

[Part B]

Start main presentation (commence 01:00 latest or earlier)

SLIDE 15 BRIEF EXPLANATION OF PRESENTATIONS

START OF PRESENTATION *- in 4 parts, with each part followed by discussion*

[Part C]

FINAL DISCUSSION ITEMS [Part C] [moderator]

1. "What are the benefits of being diagnosed with osteoporosis and what are the downsides?"
2. "How do you respond to the idea that the definition of osteoporosis defines the bones of young women as normal?“
3. "Had you ever thought before about the way this condition was defined?“
4. Any general questions/comments?

HAND OUT WRITTEN QUESTIONS

CONCLUSION

- *Thank participants, conveying to them why this is important new research; distribute remuneration and get signatures for remuneration.*
- *Explain food outside room*
