# Supplementary material for: Communicating about overdiagnosis: Learning from community focus groups on osteoporosis
Source: PLoS One. 2017 Feb 3;12(2):e0170142. doi: 10.1371/journal.pone.0170142 (PMC5291414; doi:10.1371/journal.pone.0170142)
Supplement: S3 Text — (PPTX) [file pone.0170142.s003.pptx]

## Slide 1
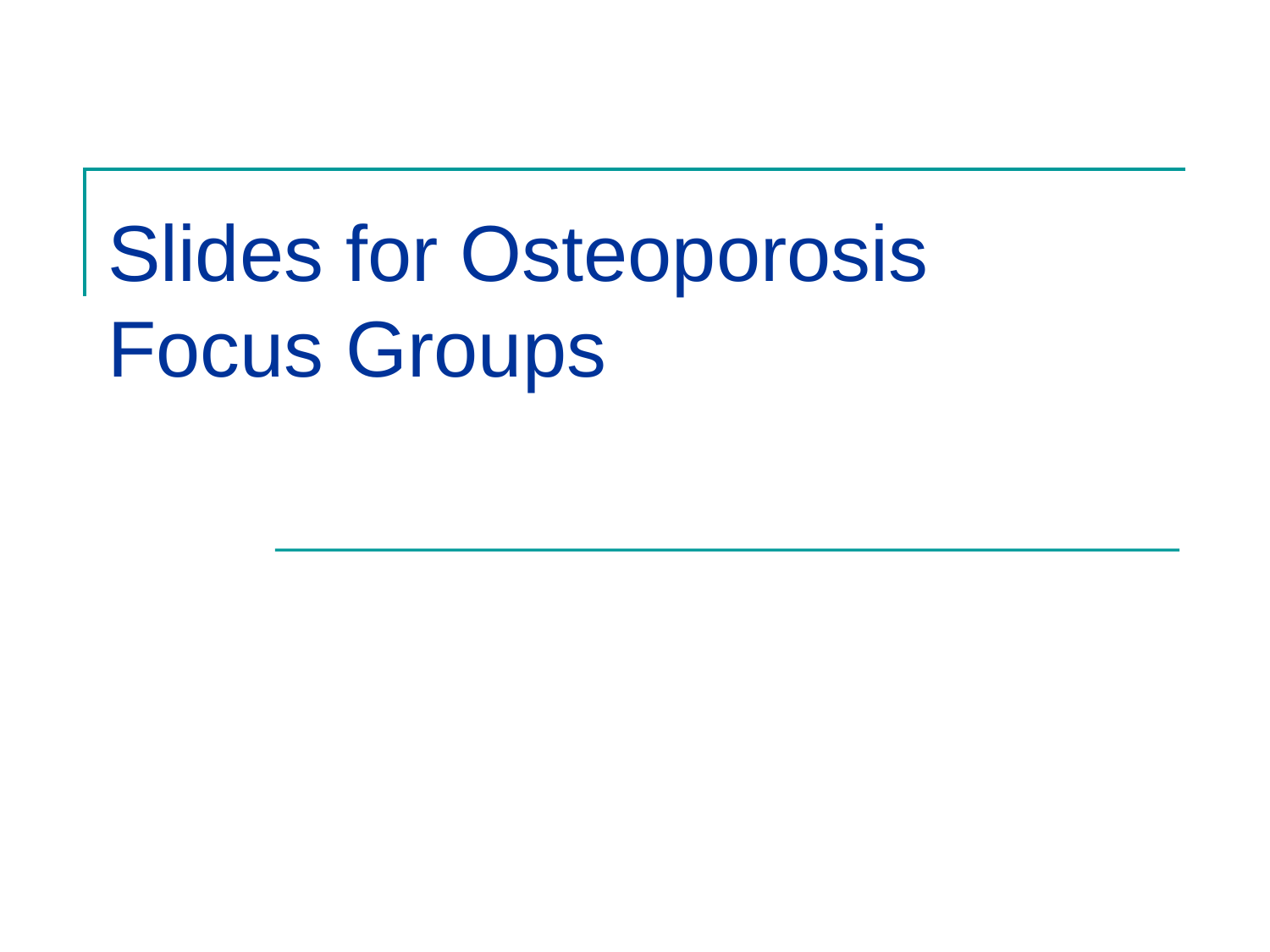

# Slides for Osteoporosis Focus Groups

## Slide 2
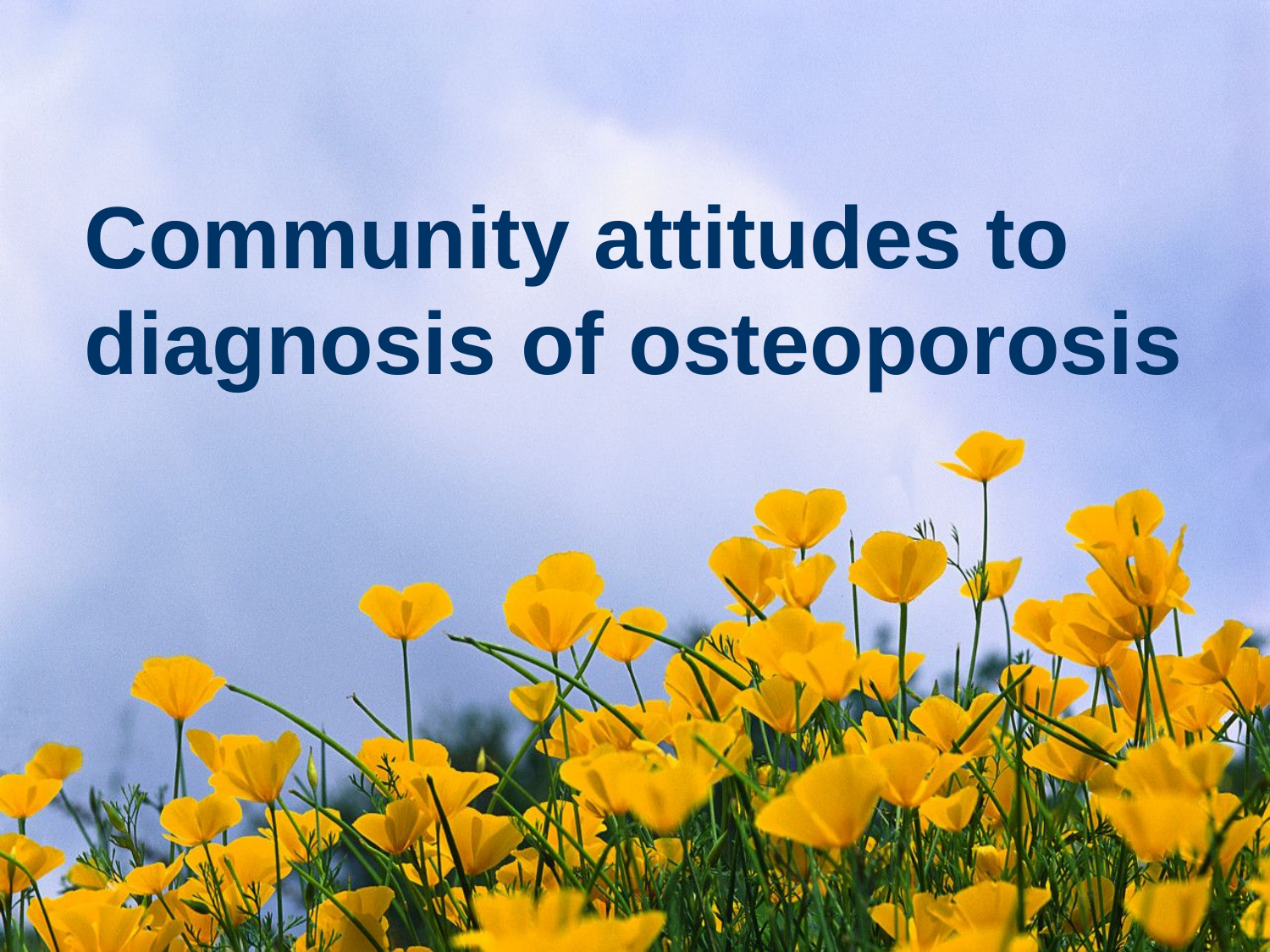

# Community attitudes to diagnosis of osteoporosis
2

## Slide 3
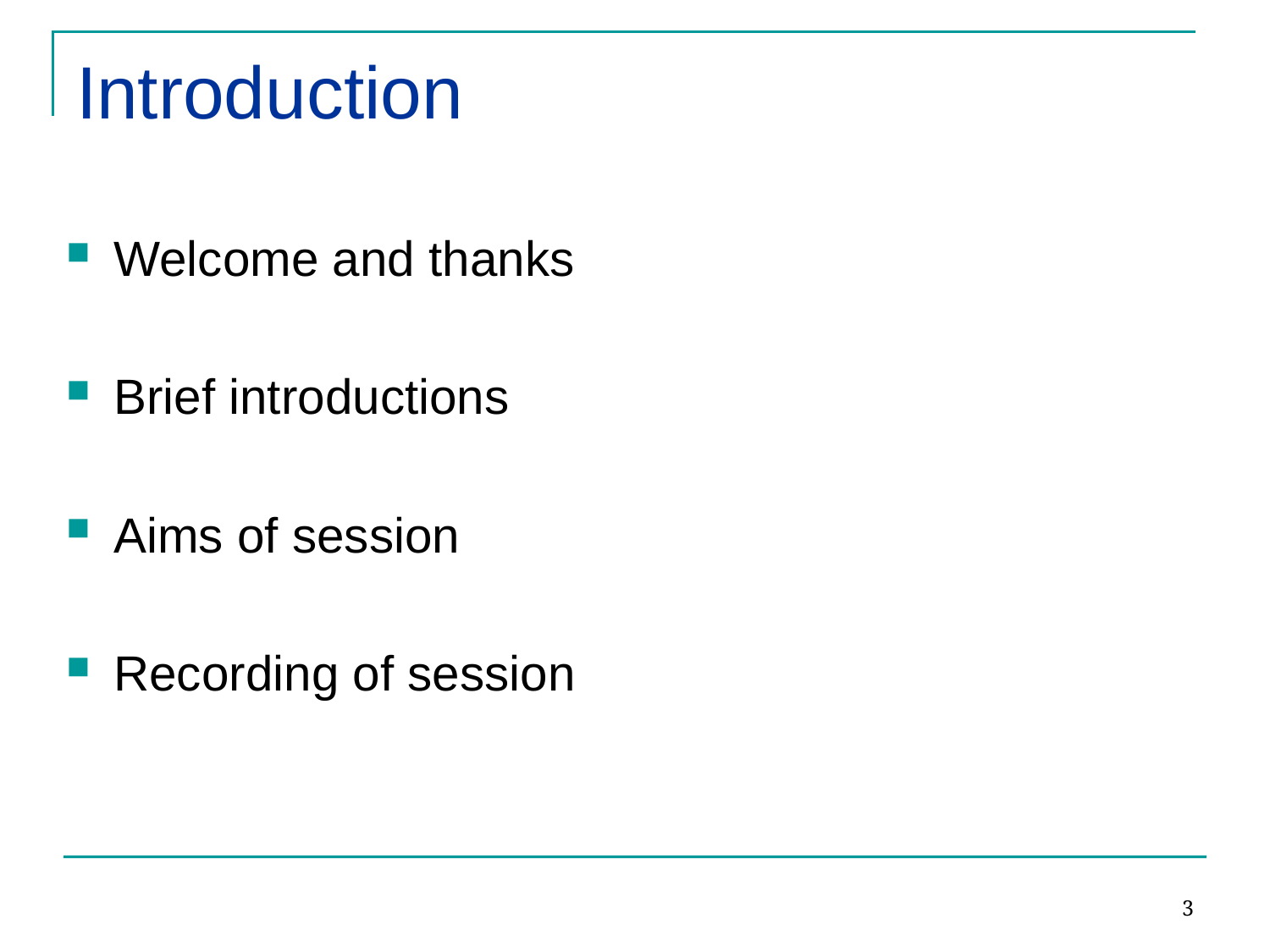

# Introduction
Welcome and thanks
Brief introductions
Aims of session
Recording of session
3

## Slide 4
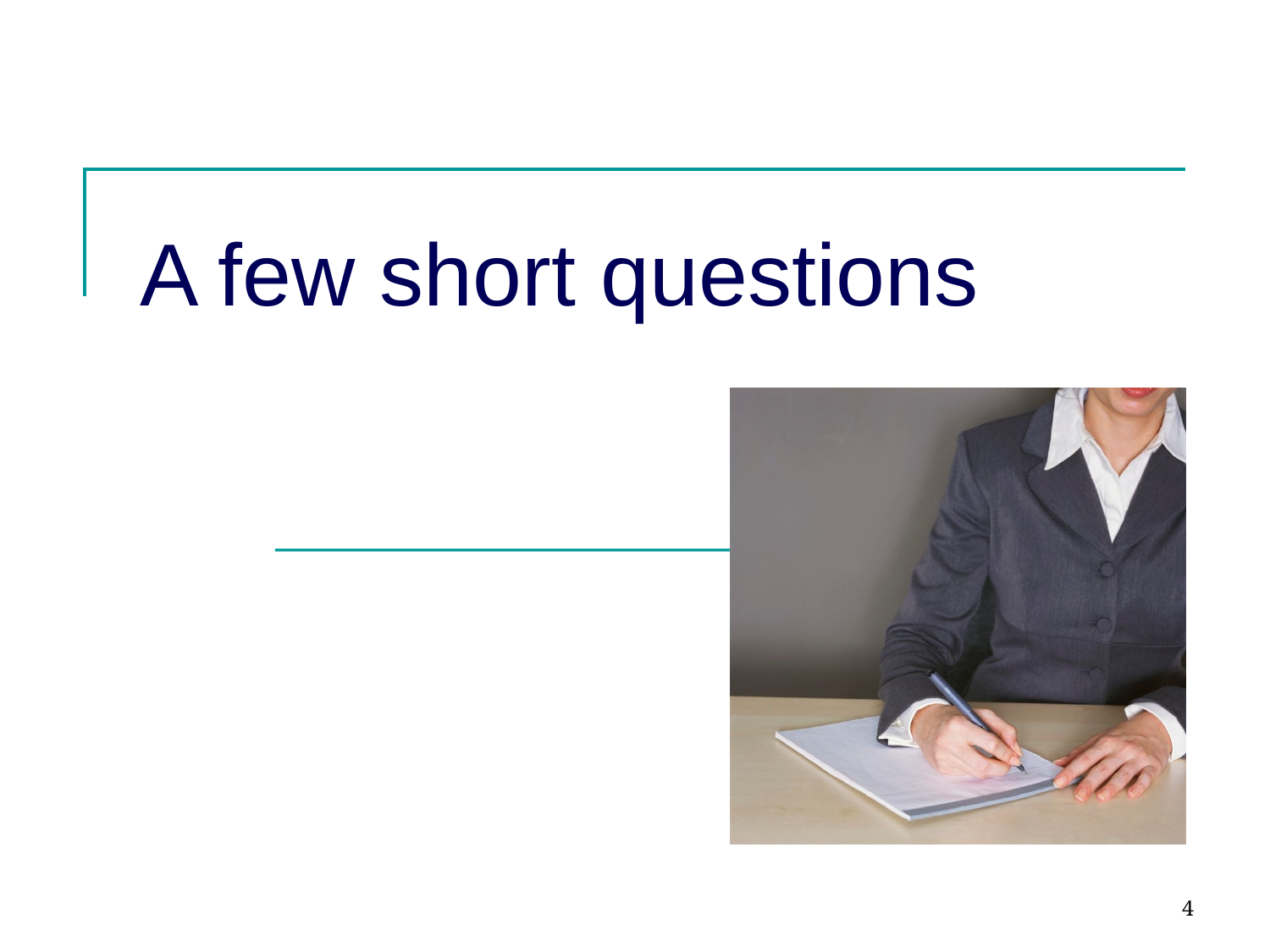

# A few short questions
4

## Slide 5
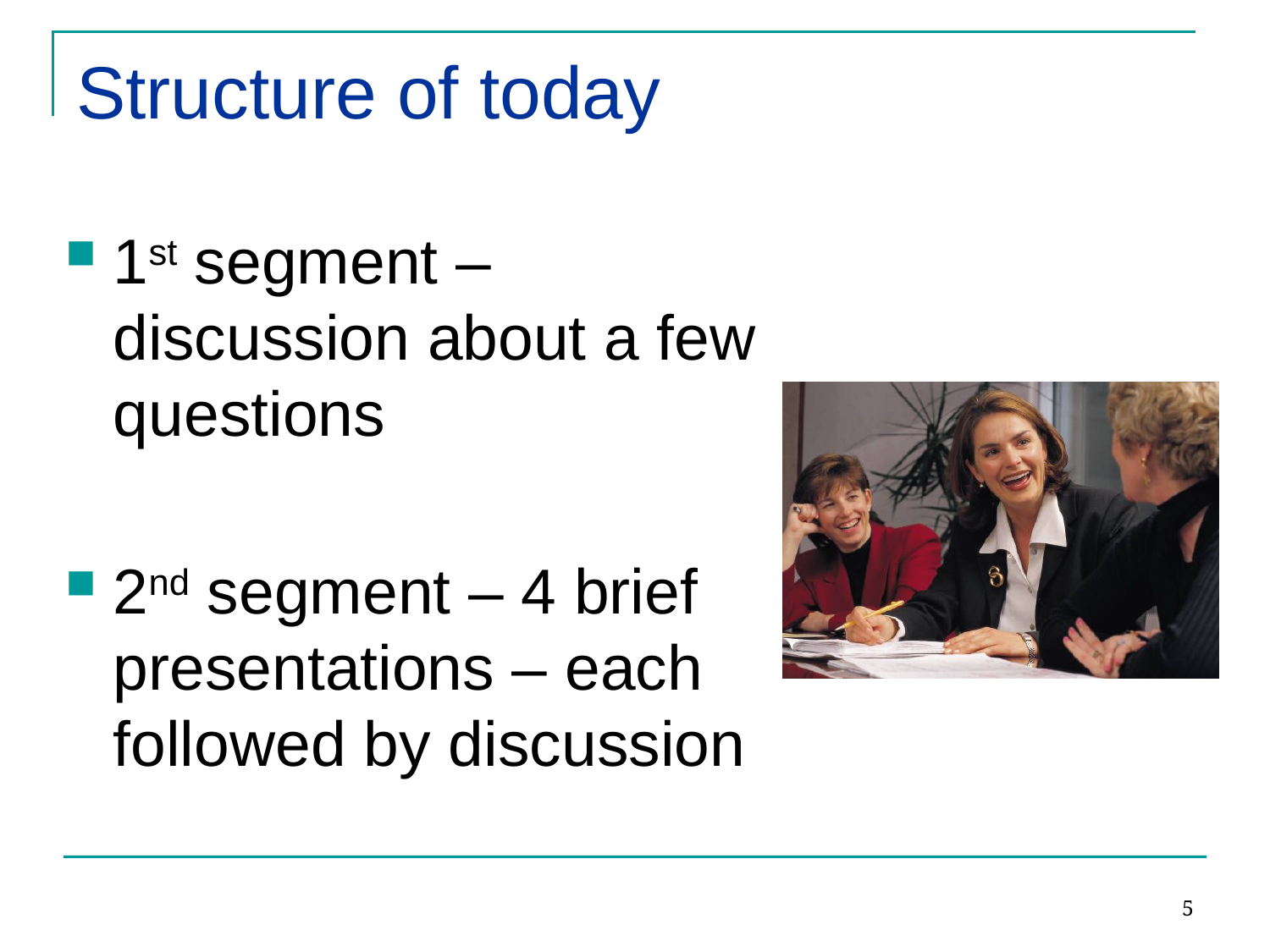

# Structure of today
1st segment – discussion about a few questions
2nd segment – 4 brief presentations – each followed by discussion
5

## Slide 6
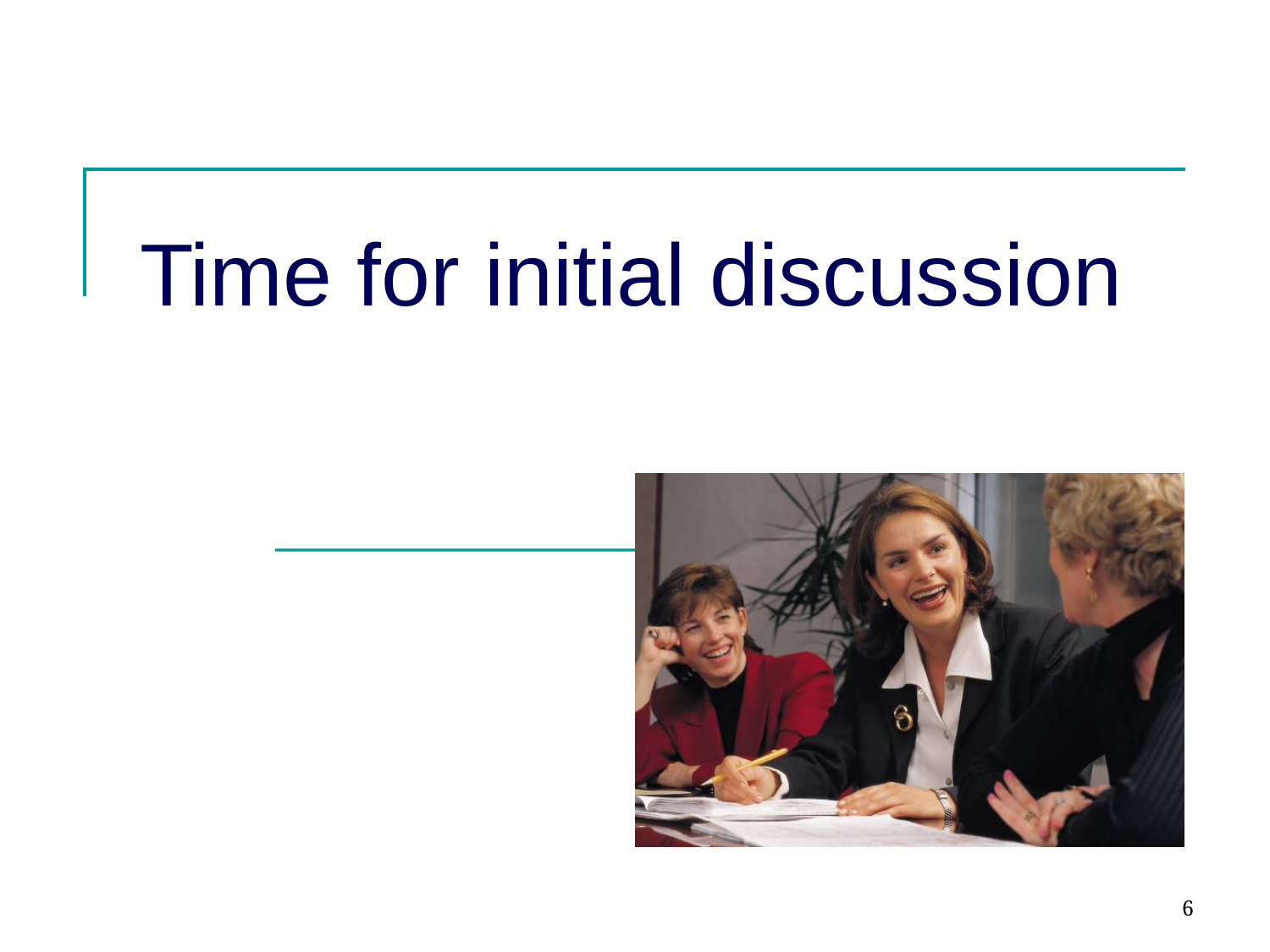

# Time for initial discussion
6

## Slide 7
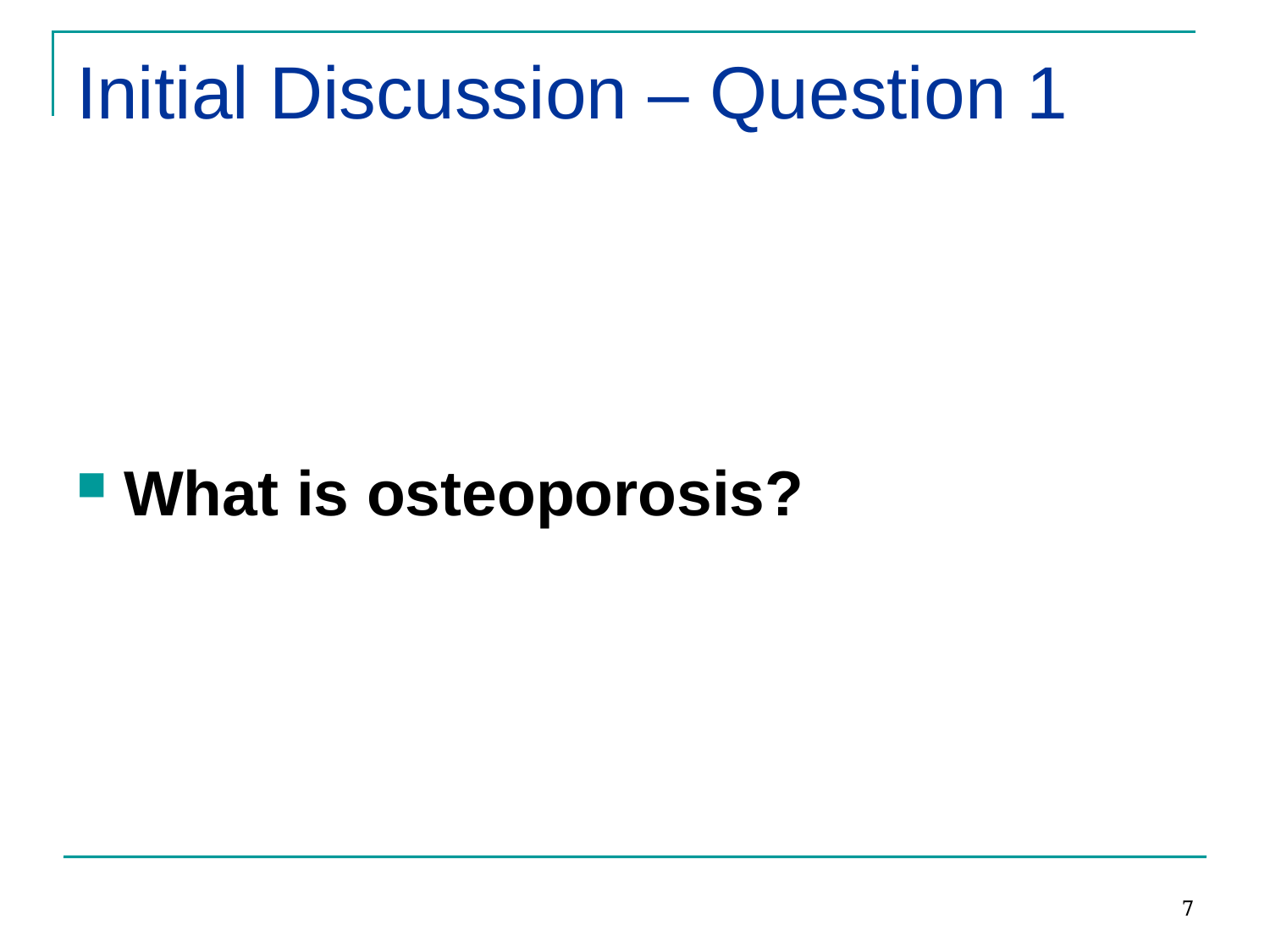

# Initial Discussion – Question 1
What is osteoporosis?
7

## Slide 8
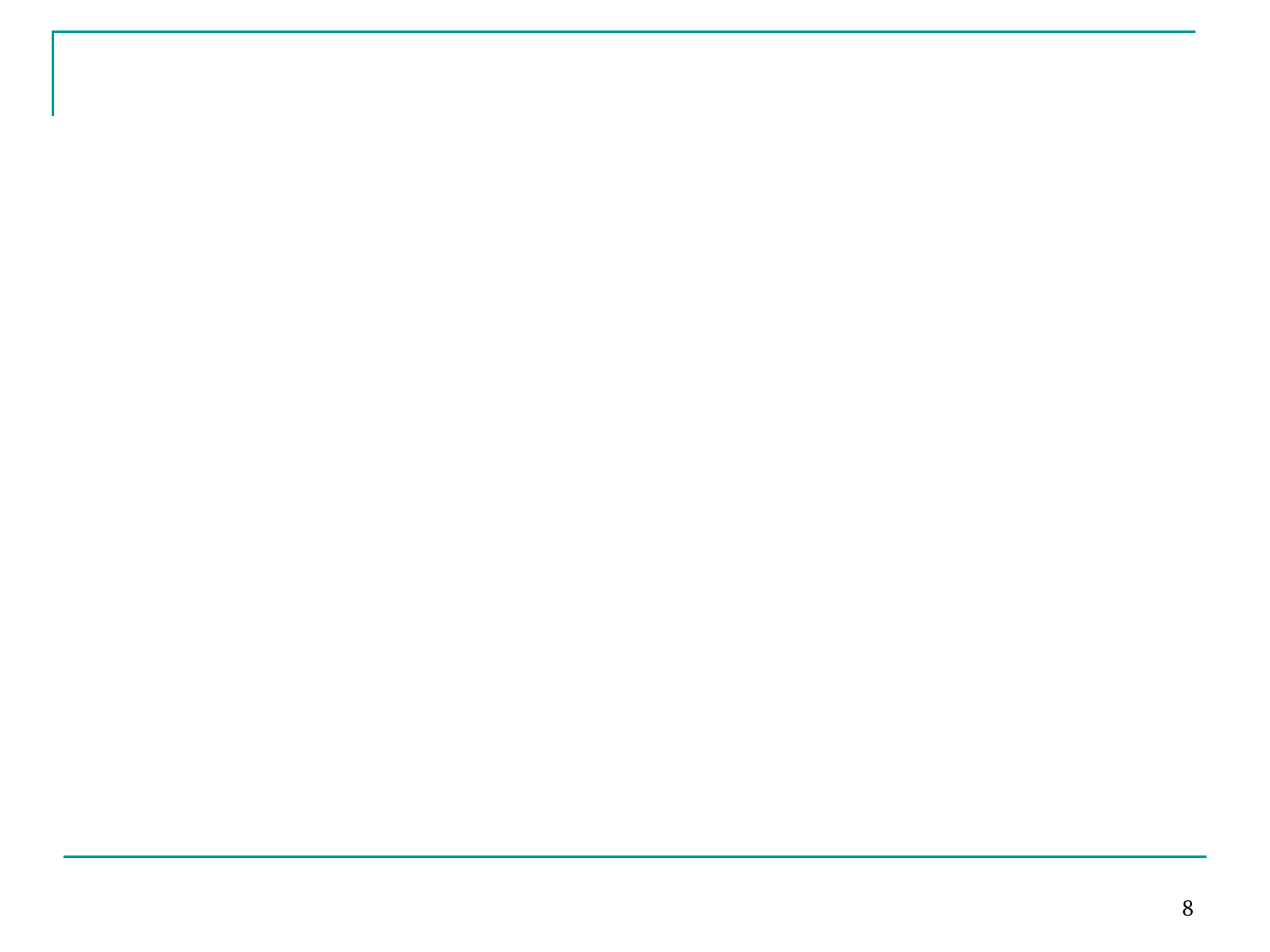

#
8

## Slide 9
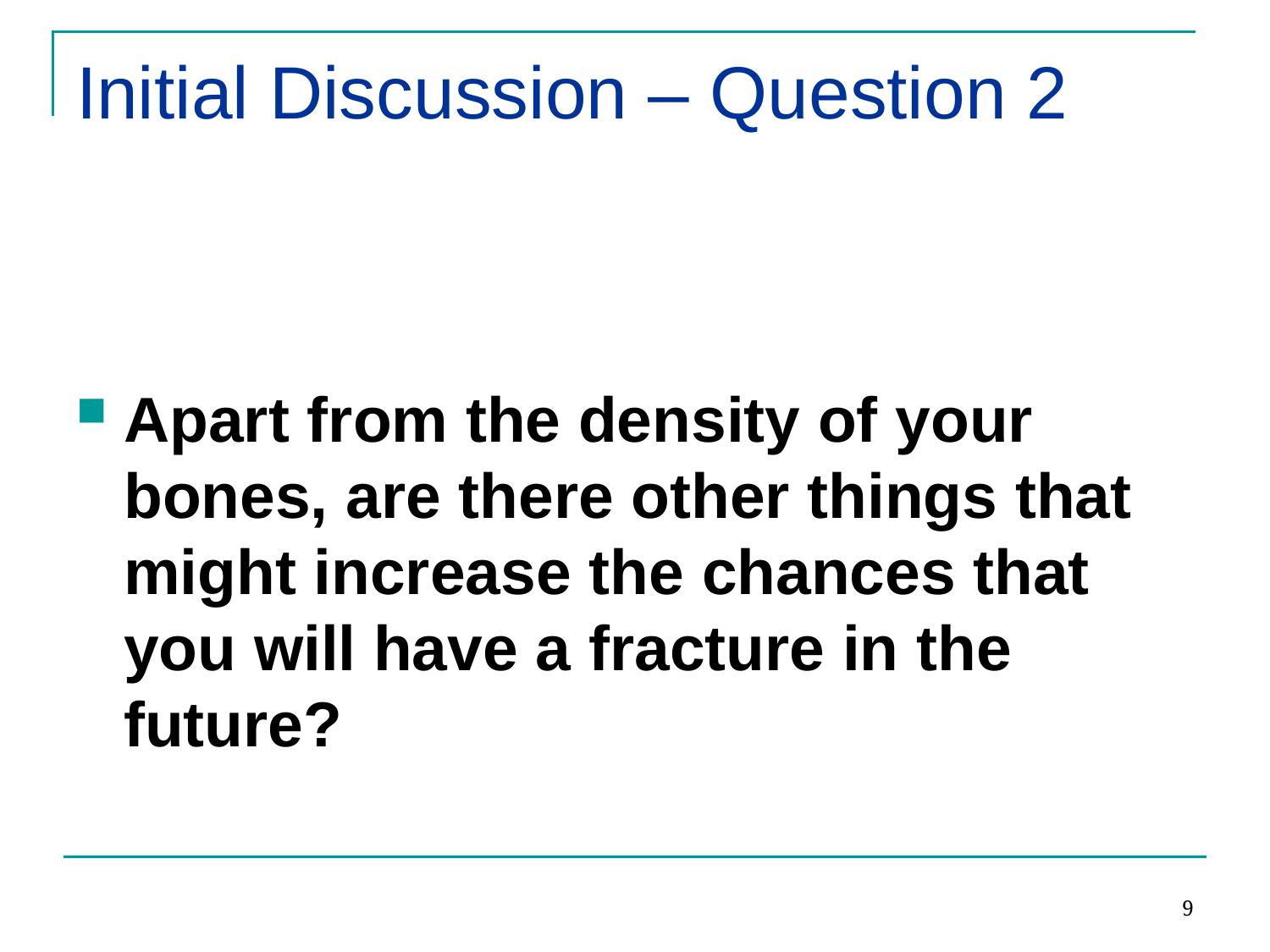

# Initial Discussion – Question 2
Apart from the density of your bones, are there other things that might increase the chances that you will have a fracture in the future?
9

## Slide 10
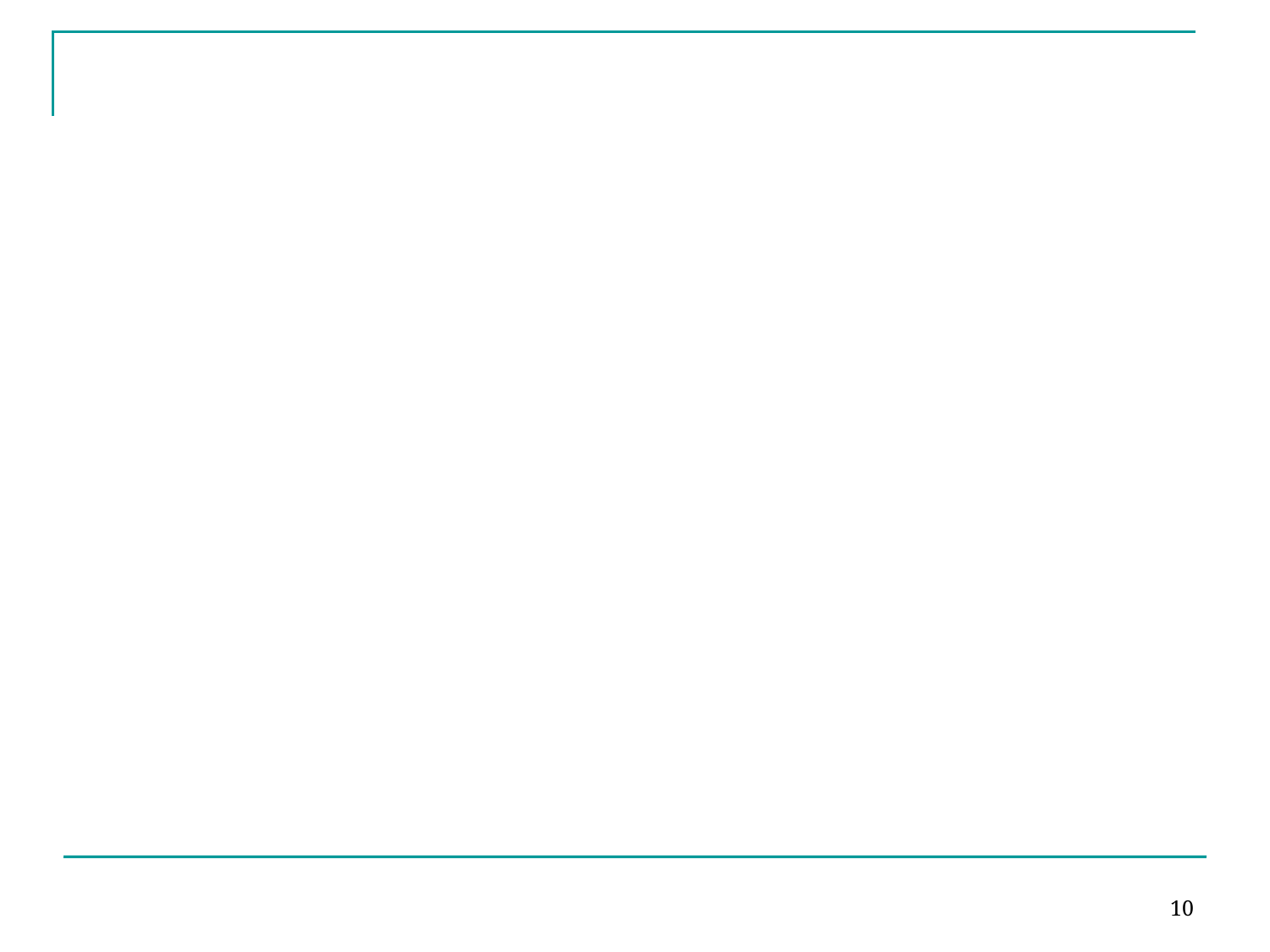

#
10

## Slide 11
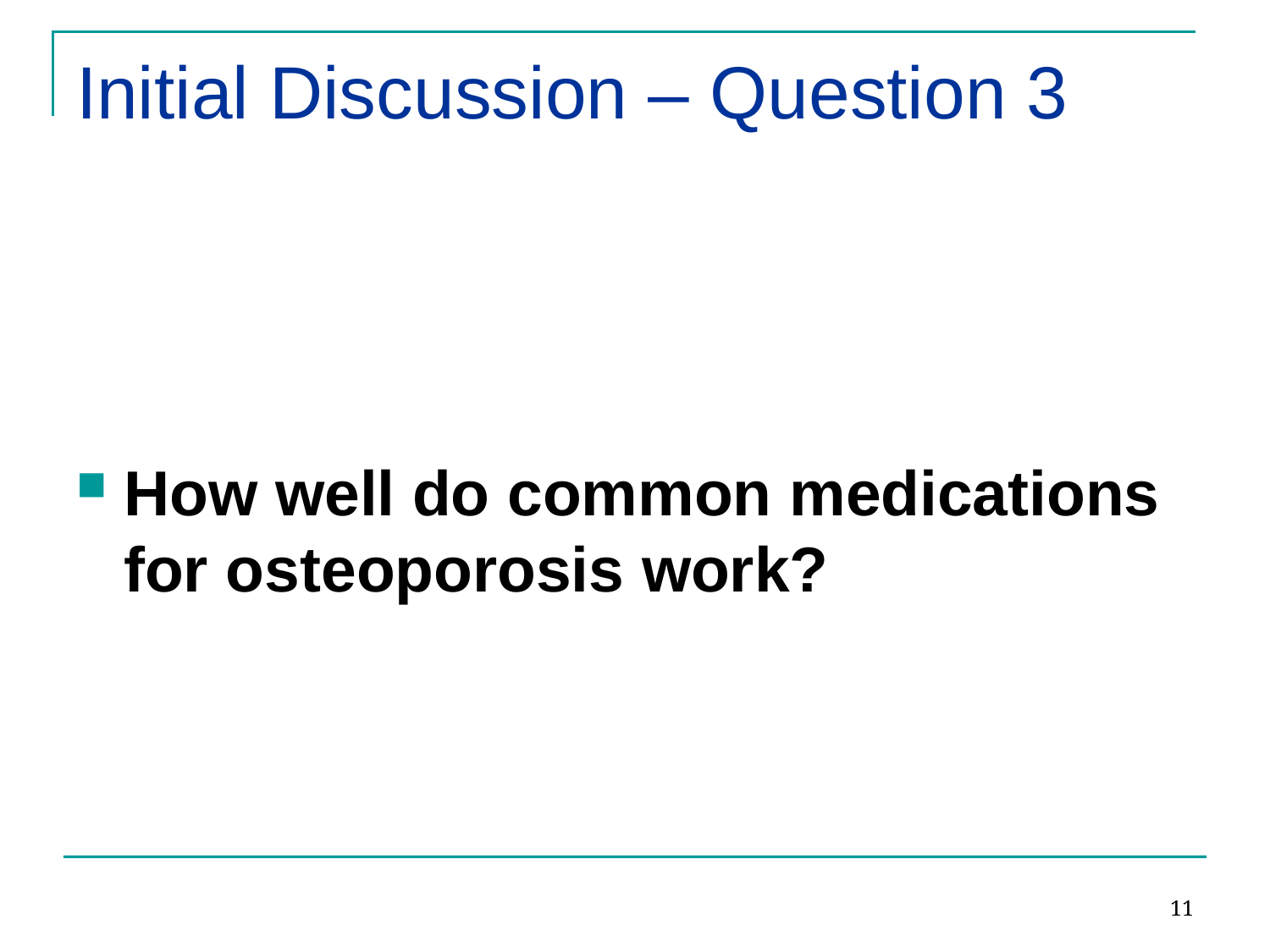

# Initial Discussion – Question 3
How well do common medications for osteoporosis work?
11

## Slide 12
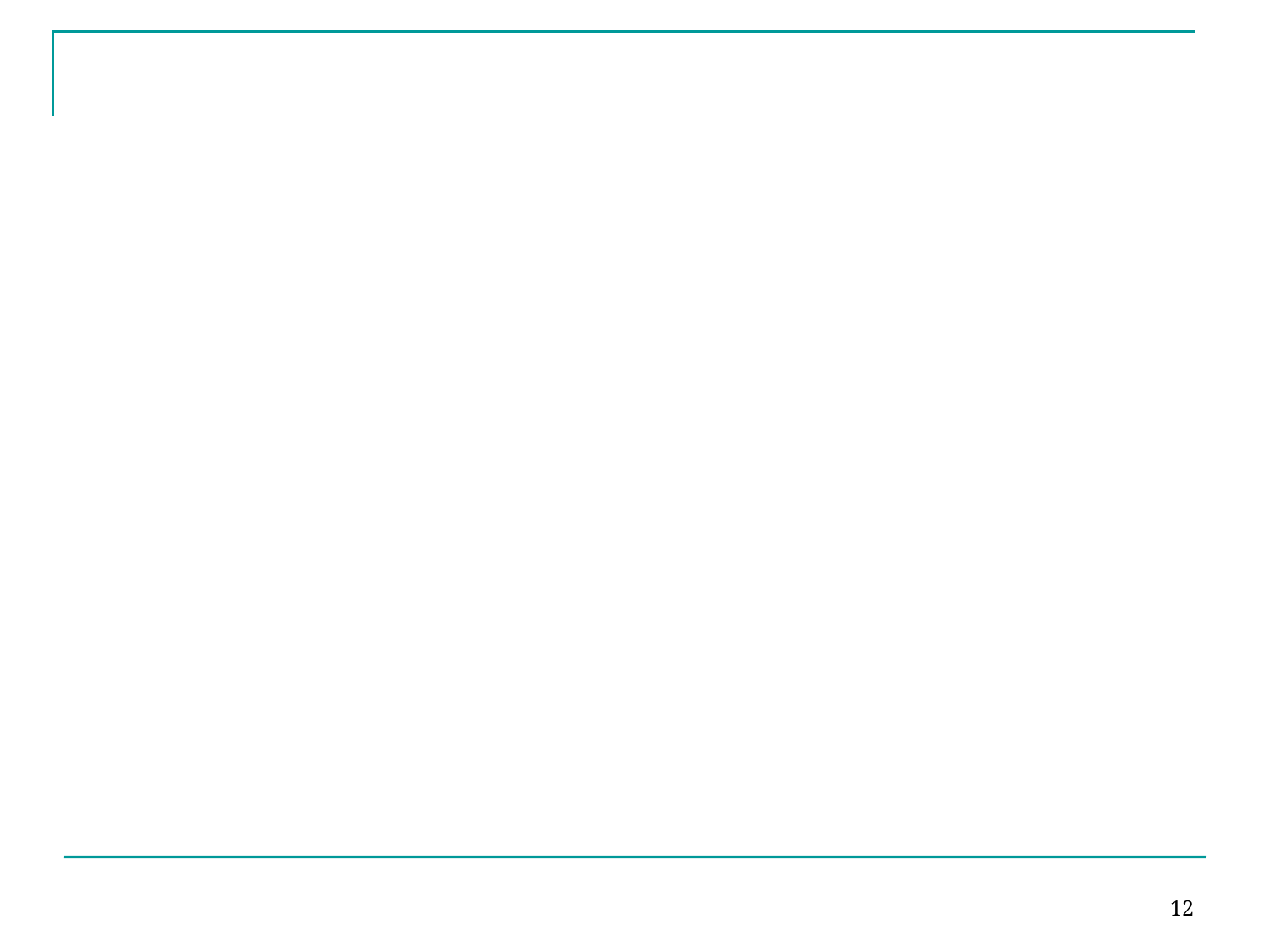

#
12

## Slide 13
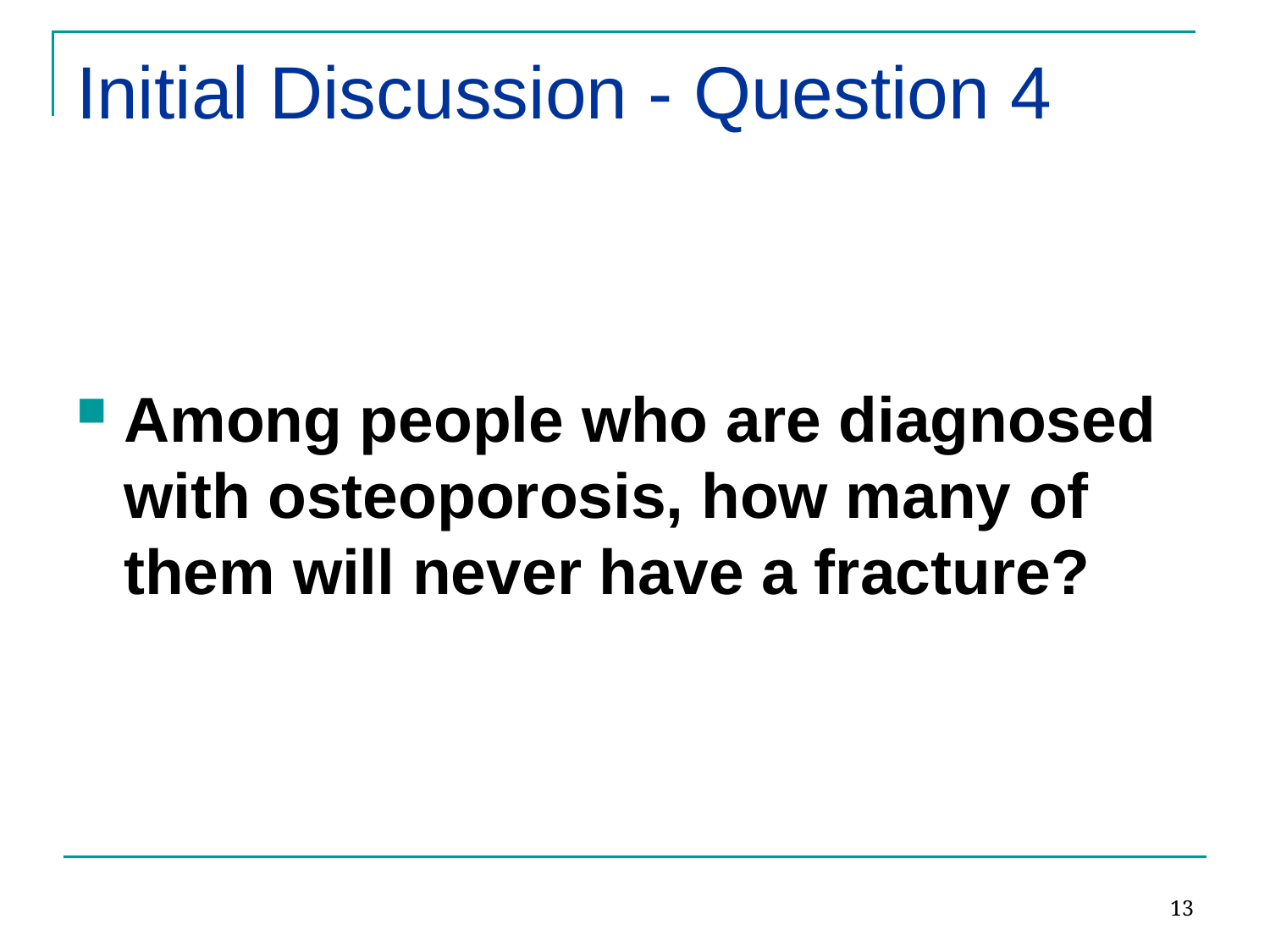

# Initial Discussion - Question 4
Among people who are diagnosed with osteoporosis, how many of them will never have a fracture?
13

## Slide 14
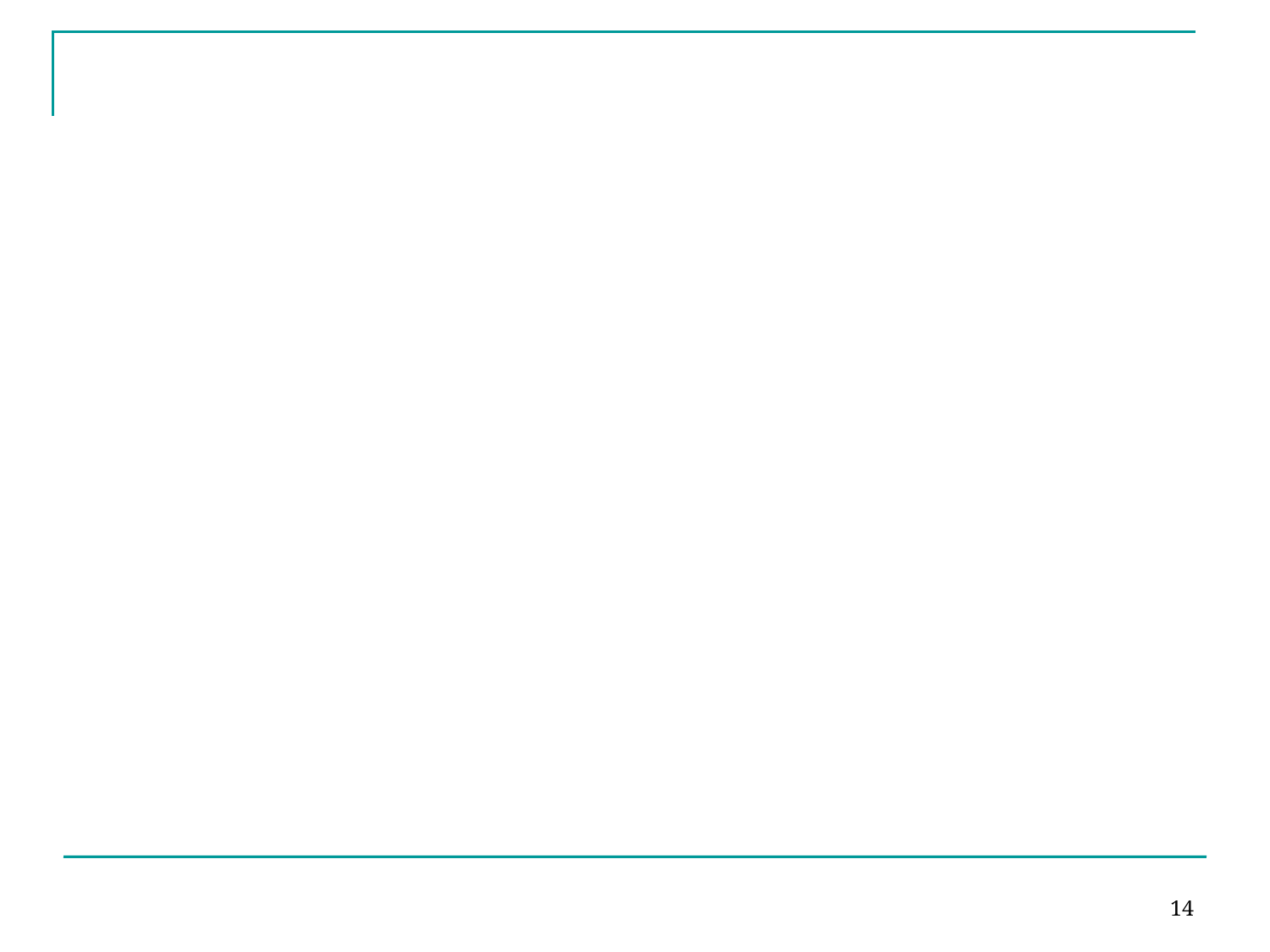

#
14

## Slide 15
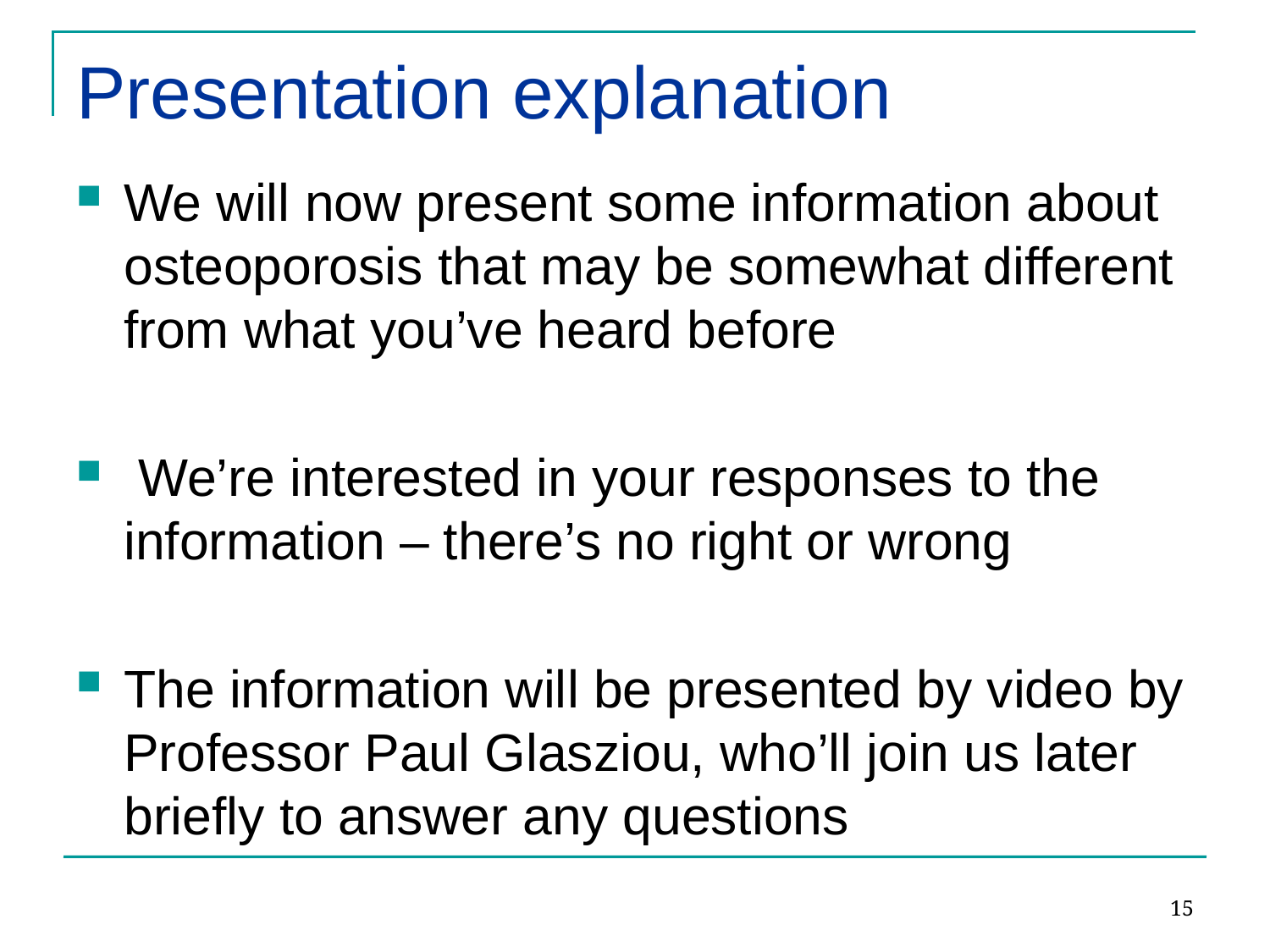

# Presentation explanation
We will now present some information about osteoporosis that may be somewhat different from what you’ve heard before
 We’re interested in your responses to the information – there’s no right or wrong
The information will be presented by video by Professor Paul Glasziou, who’ll join us later briefly to answer any questions
15

## Slide 16
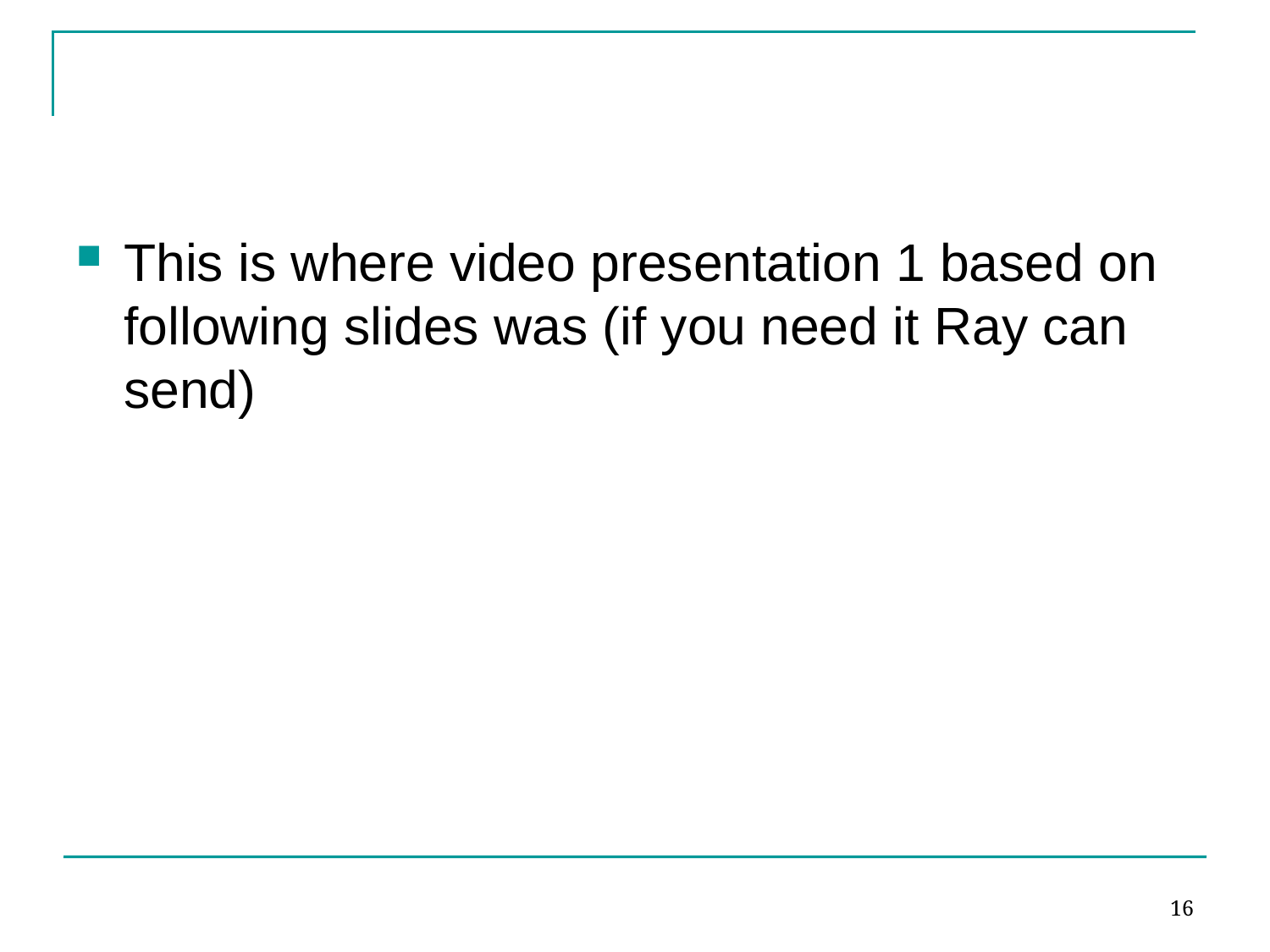

#
This is where video presentation 1 based on following slides was (if you need it Ray can send)
16

## Slide 17
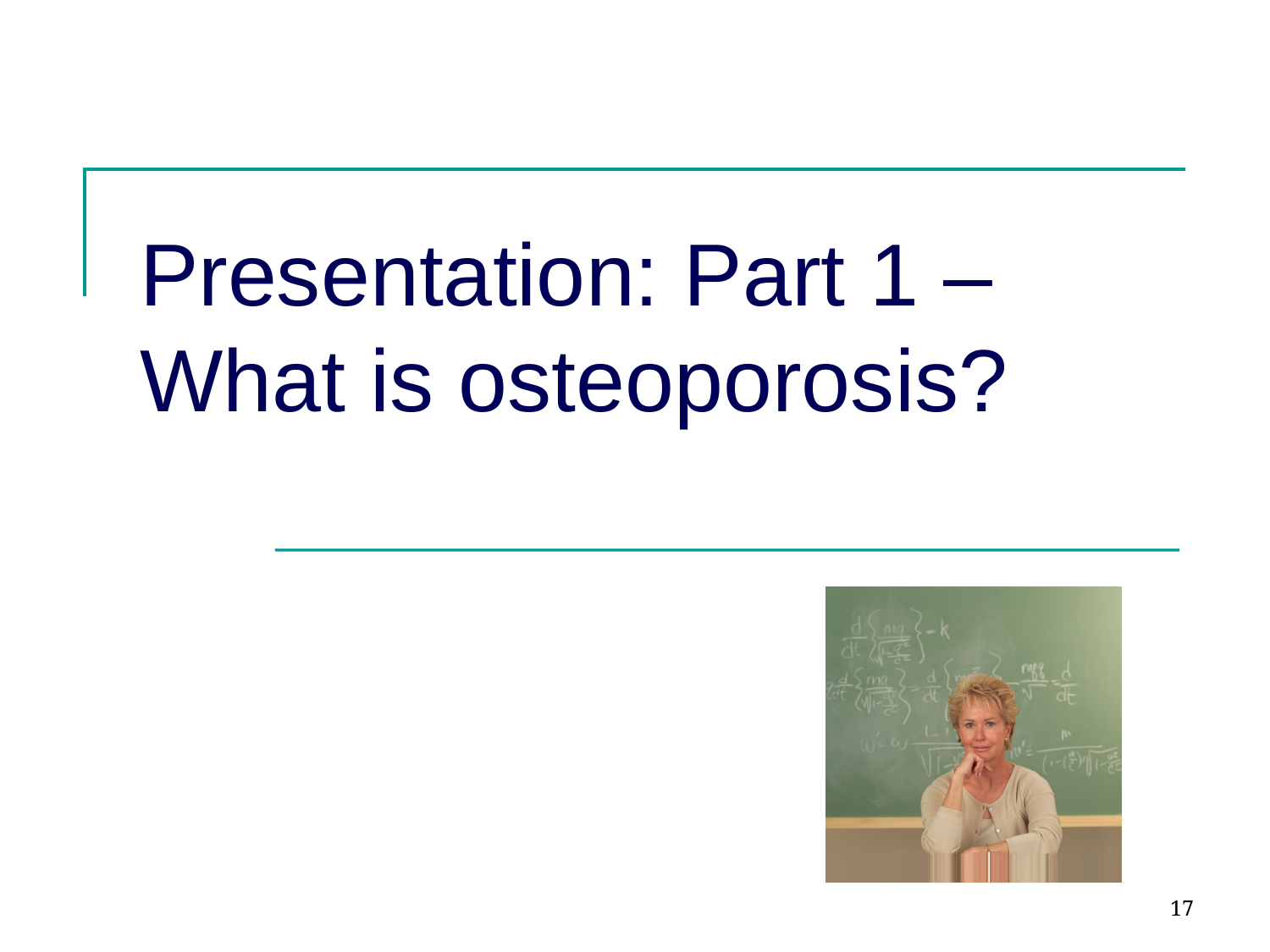

# Presentation: Part 1 – What is osteoporosis?
17

## Slide 18
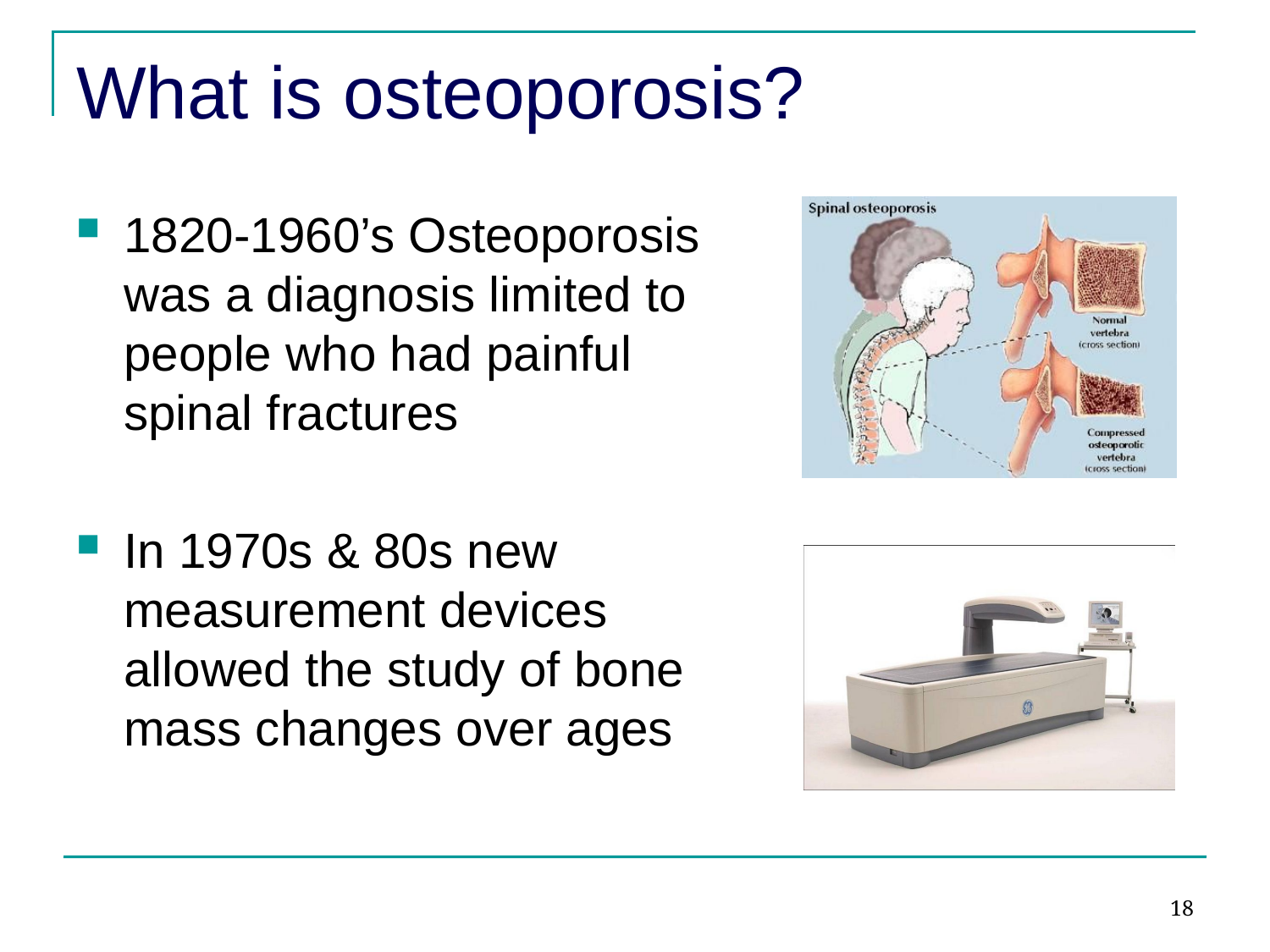

# What is osteoporosis?
1820-1960’s Osteoporosis was a diagnosis limited to people who had painful spinal fractures
In 1970s & 80s new measurement devices allowed the study of bone mass changes over ages
18

## Slide 19
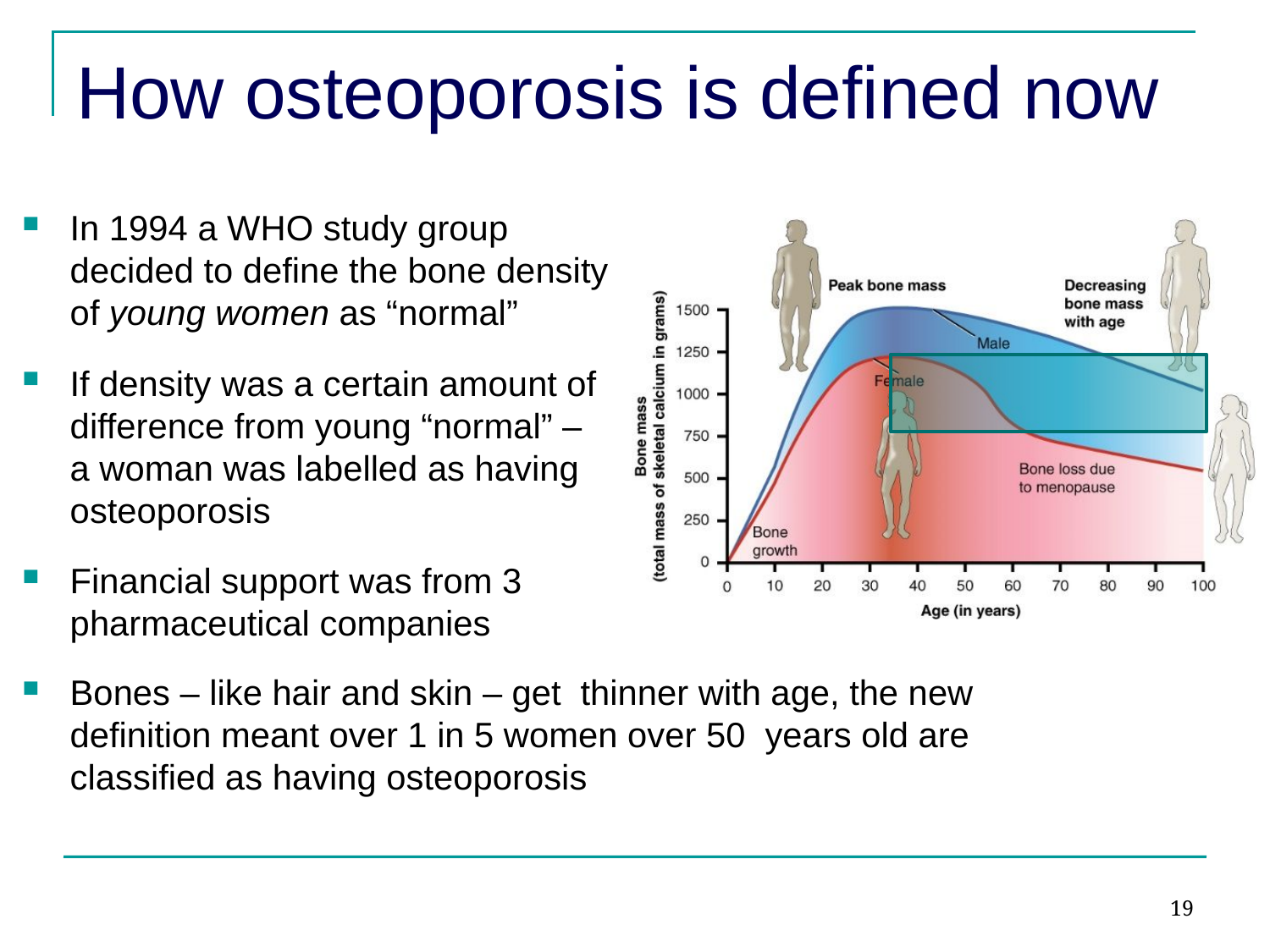

# How osteoporosis is defined now
In 1994 a WHO study group decided to define the bone density of young women as “normal”
If density was a certain amount of difference from young “normal” – a woman was labelled as having osteoporosis
Financial support was from 3 pharmaceutical companies
Bones – like hair and skin – get thinner with age, the new definition meant over 1 in 5 women over 50 years old are classified as having osteoporosis
19

## Slide 20
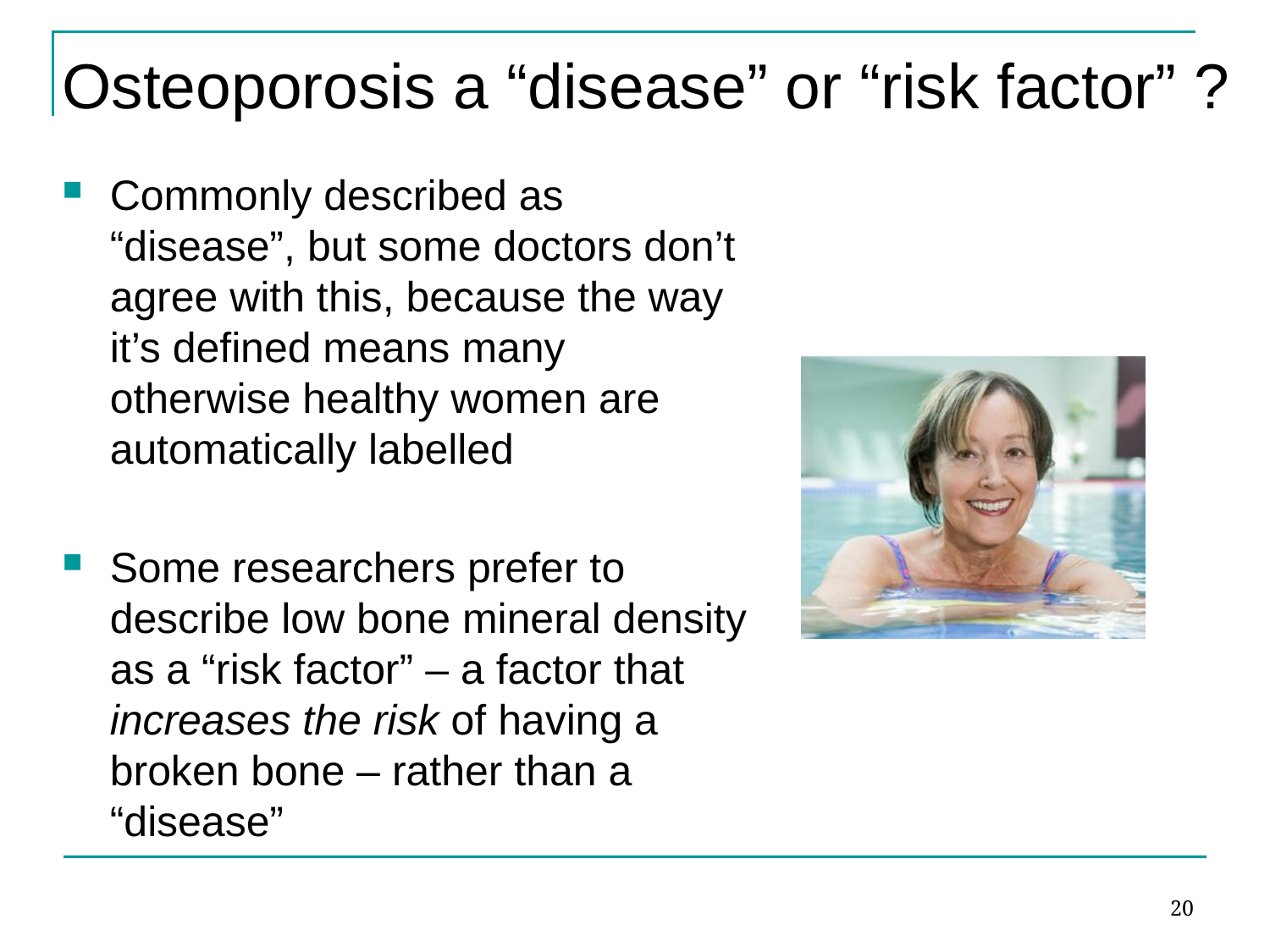

# Osteoporosis a “disease” or “risk factor” ?
Commonly described as “disease”, but some doctors don’t agree with this, because the way it’s defined means many otherwise healthy women are automatically labelled
Some researchers prefer to describe low bone mineral density as a “risk factor” – a factor that increases the risk of having a broken bone – rather than a “disease”
20

## Slide 21
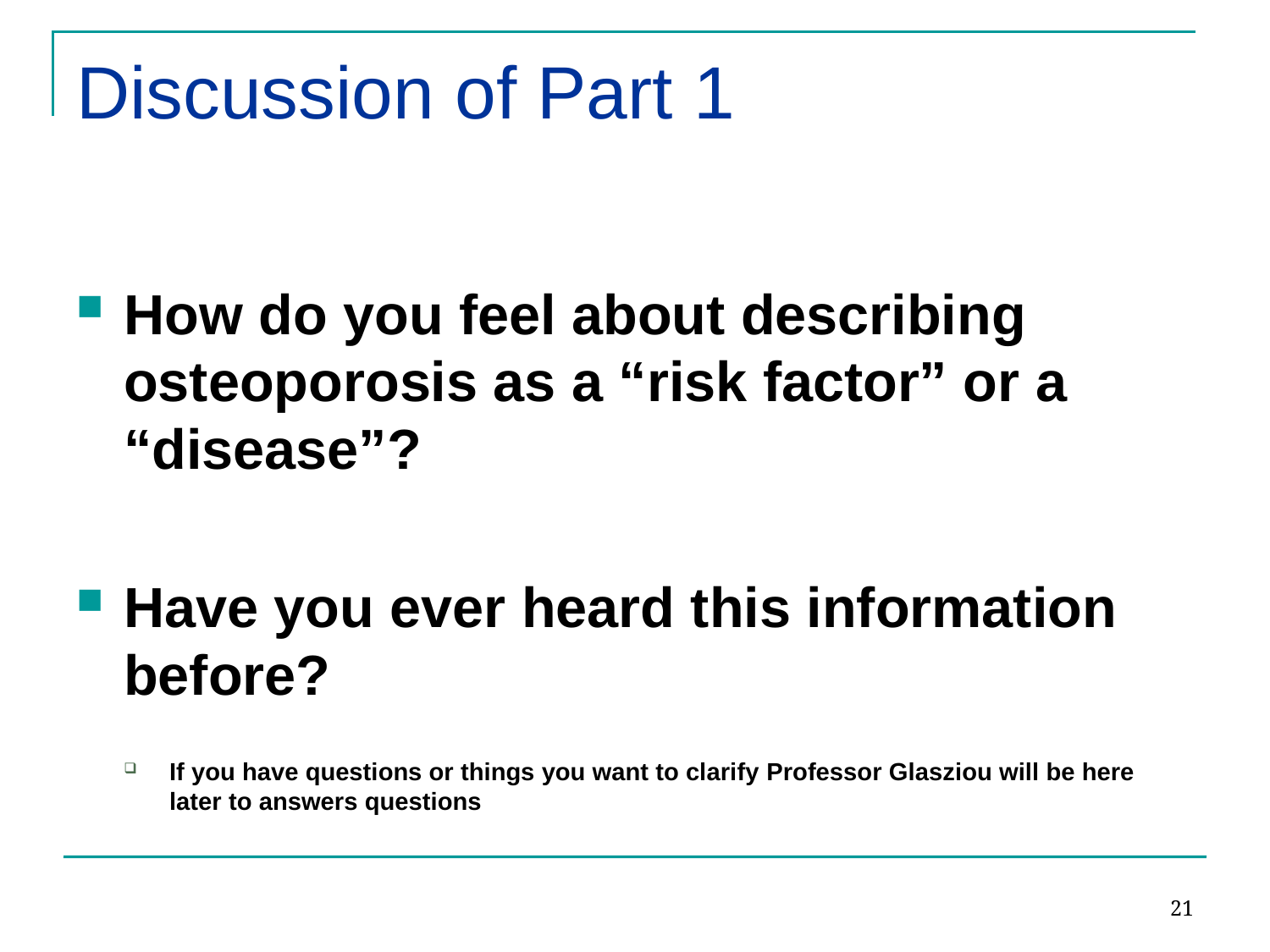

# Discussion of Part 1
How do you feel about describing osteoporosis as a “risk factor” or a “disease”?
Have you ever heard this information before?
If you have questions or things you want to clarify Professor Glasziou will be here later to answers questions
21

## Slide 22
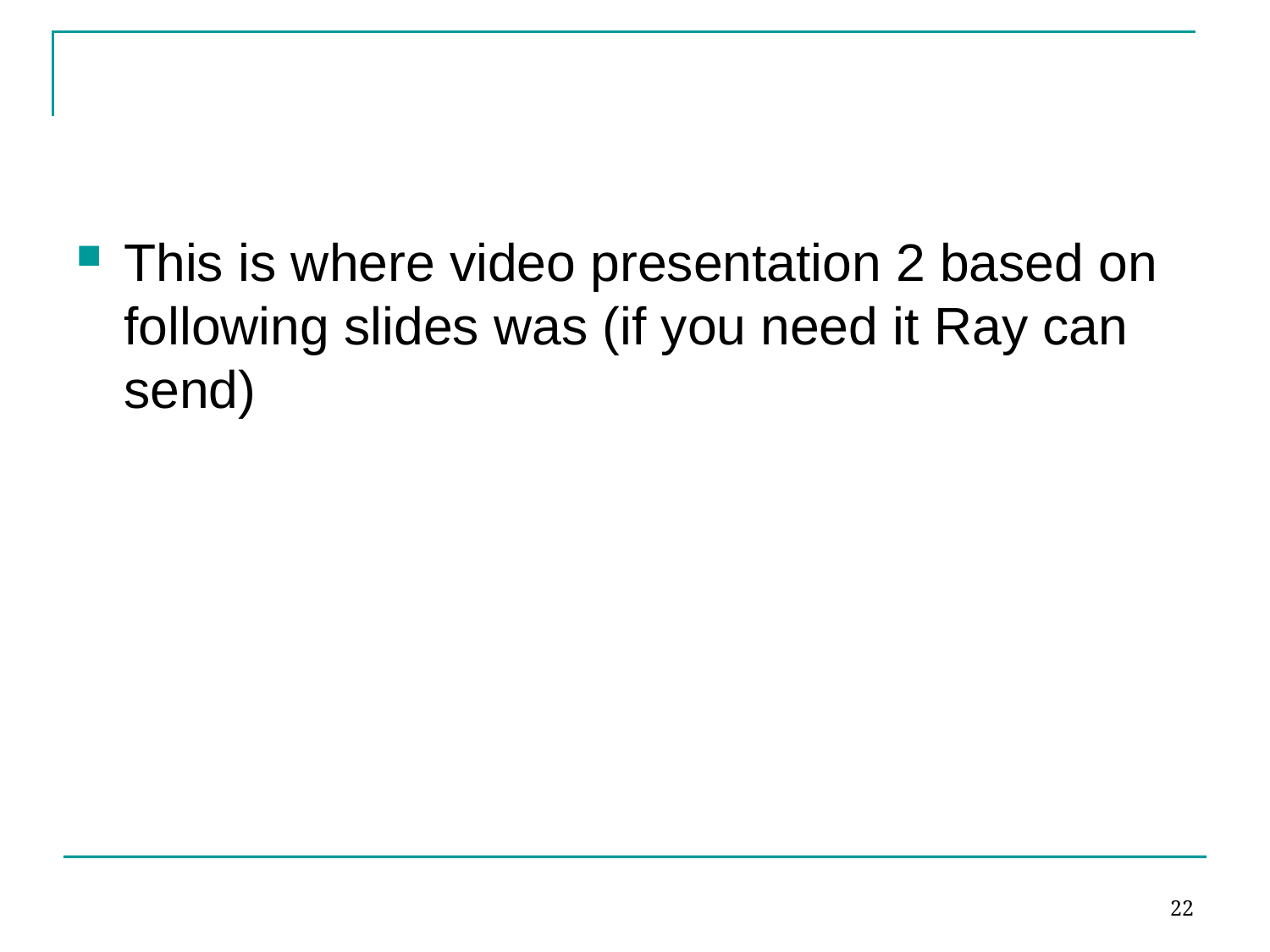

#
This is where video presentation 2 based on following slides was (if you need it Ray can send)
22

## Slide 23
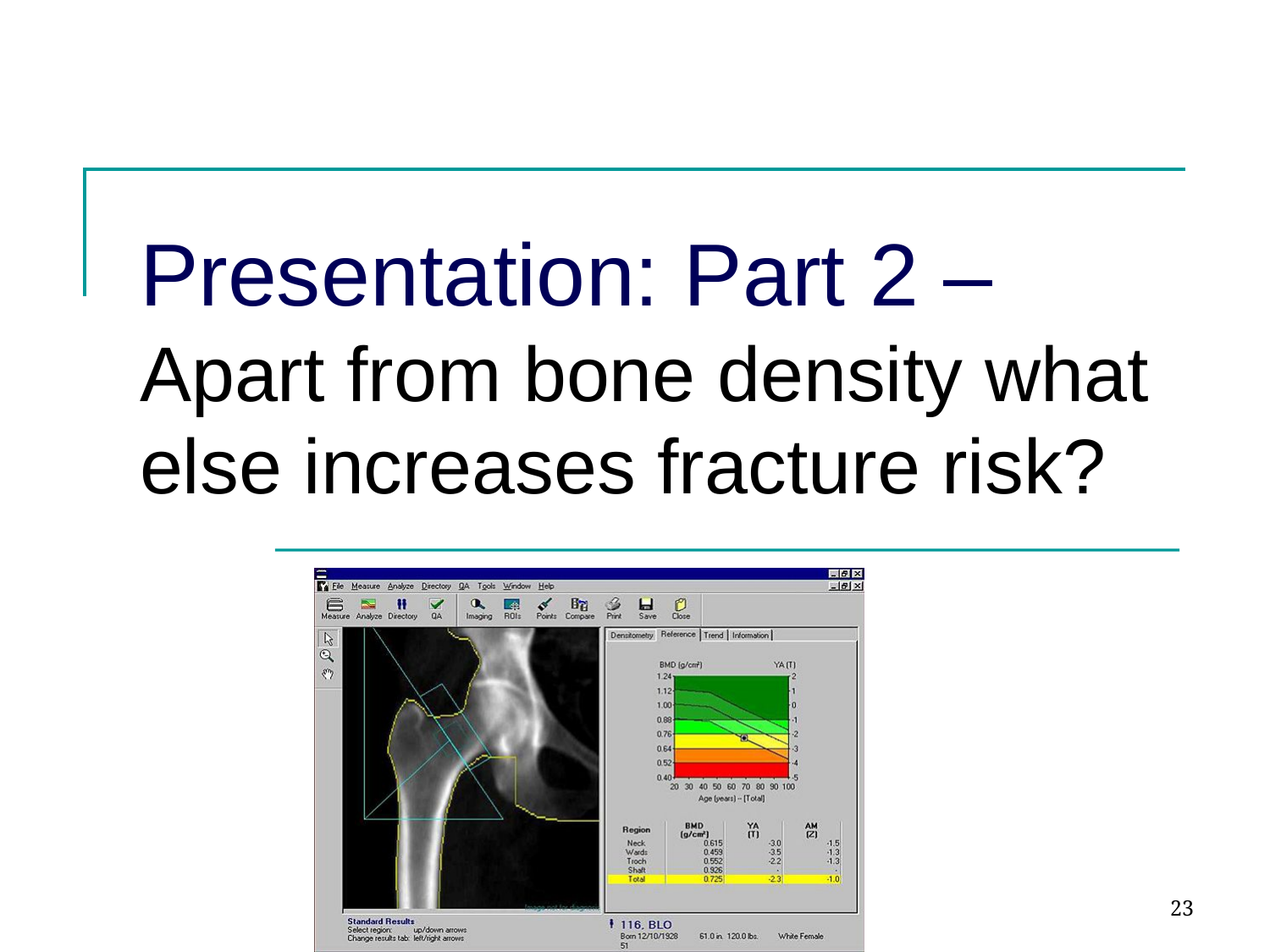

# Presentation: Part 2 – Apart from bone density what else increases fracture risk?
23

## Slide 24
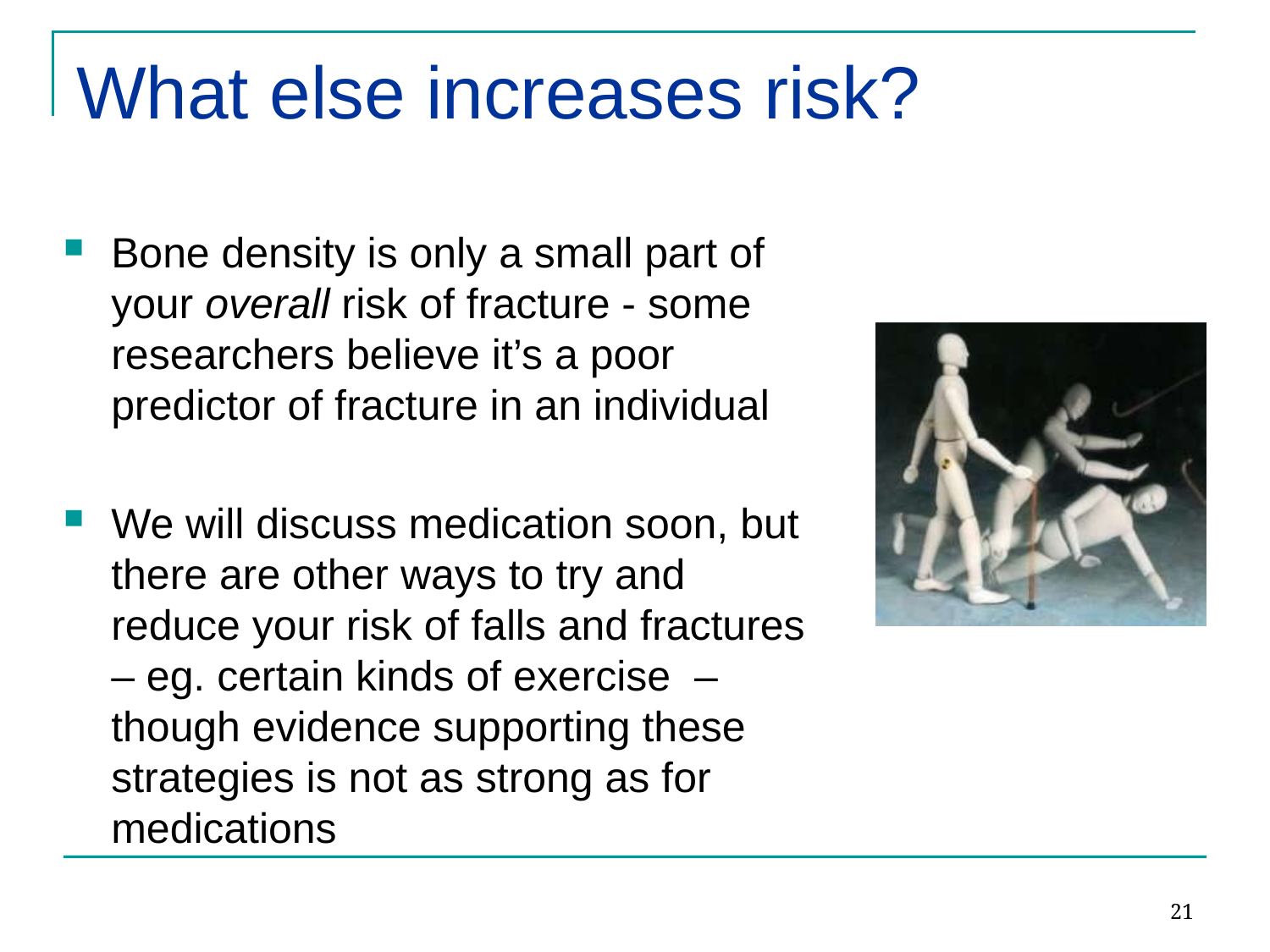

# What else increases risk?
Bone density is only a small part of your overall risk of fracture - some researchers believe it’s a poor predictor of fracture in an individual
We will discuss medication soon, but there are other ways to try and reduce your risk of falls and fractures – eg. certain kinds of exercise – though evidence supporting these strategies is not as strong as for medications
21

## Slide 25
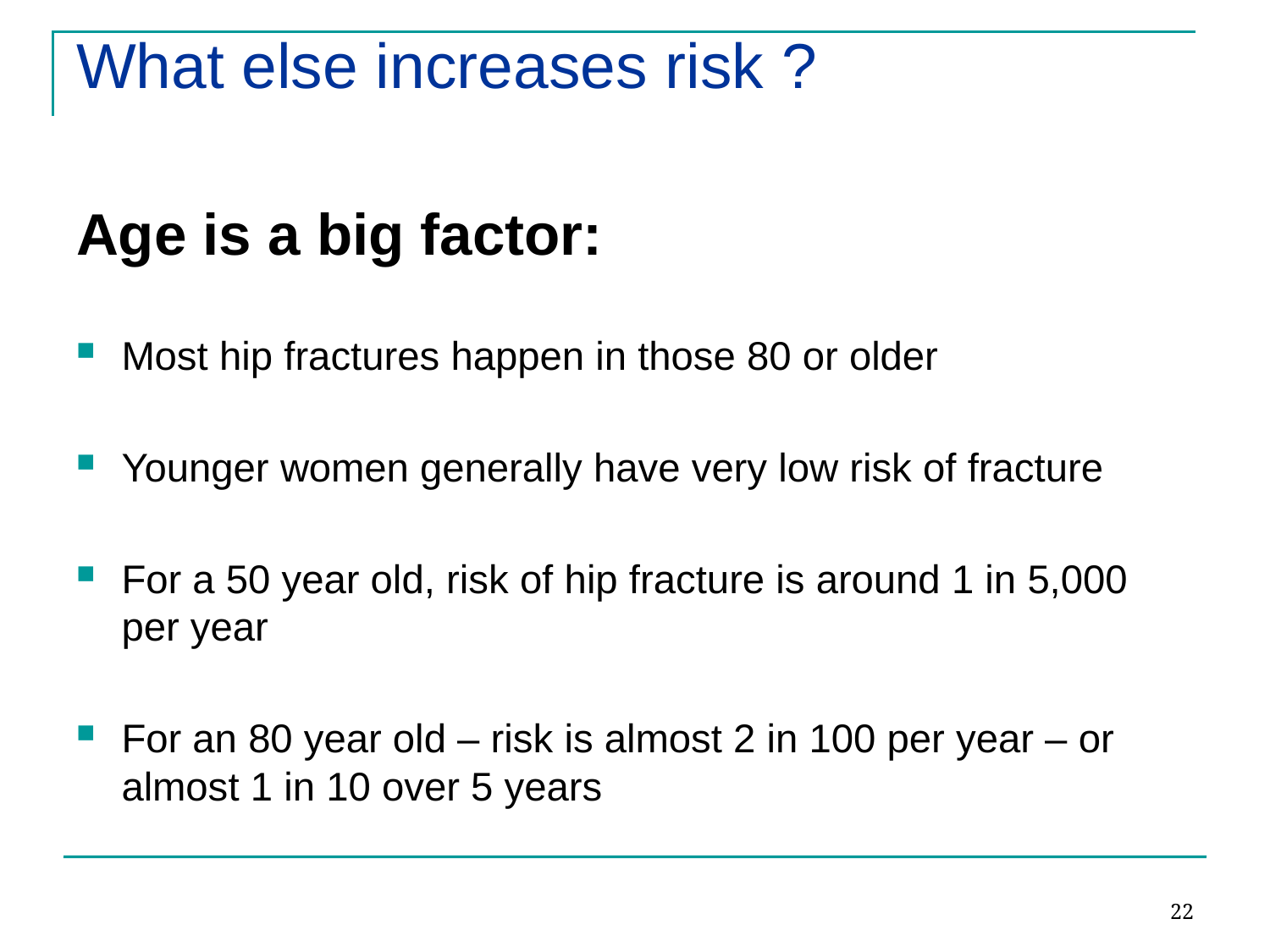

# What else increases risk ?
Age is a big factor:
Most hip fractures happen in those 80 or older
Younger women generally have very low risk of fracture
For a 50 year old, risk of hip fracture is around 1 in 5,000 per year
For an 80 year old – risk is almost 2 in 100 per year – or almost 1 in 10 over 5 years
22

## Slide 26
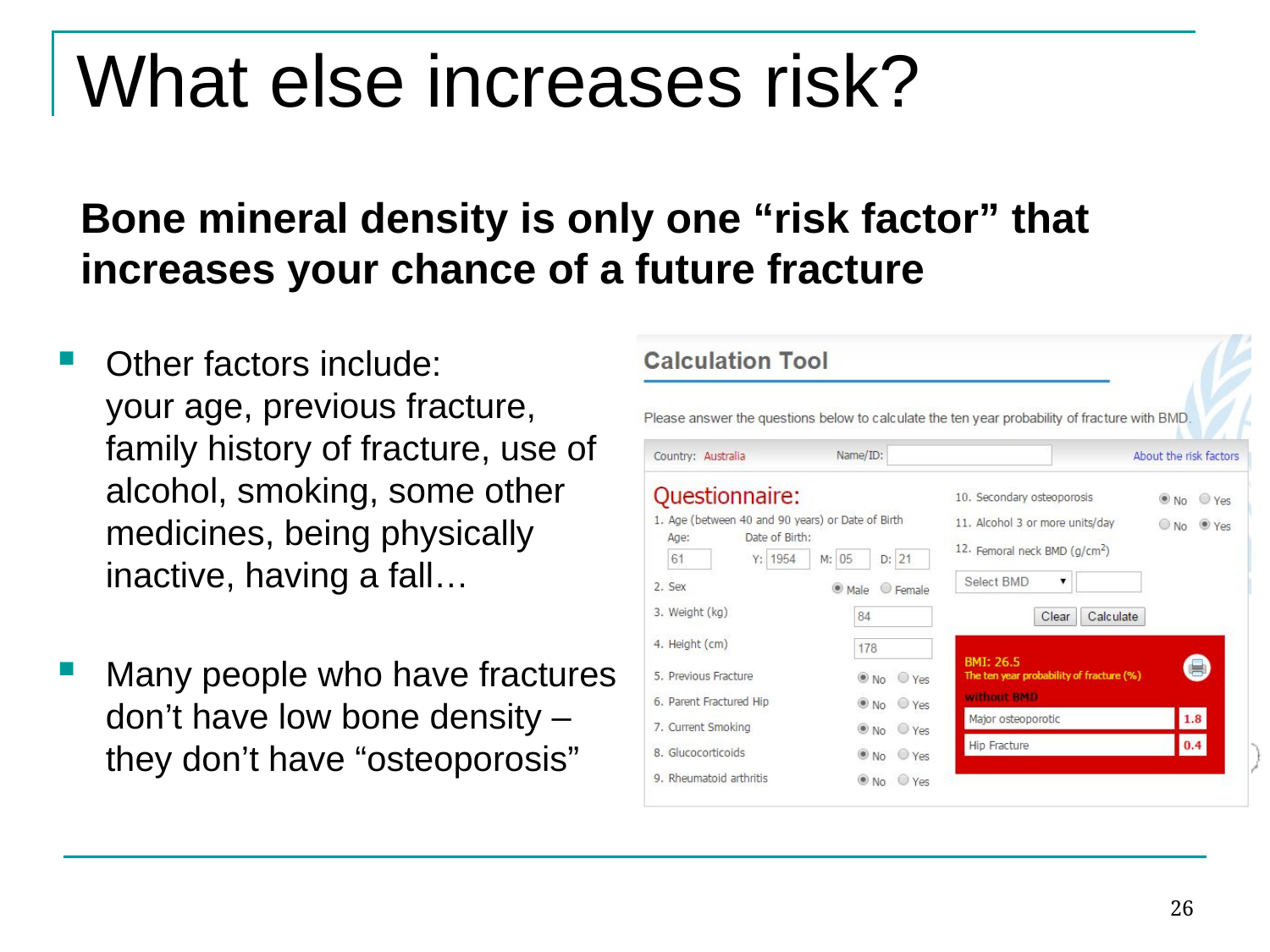

# What else increases risk?
Bone mineral density is only one “risk factor” that
increases your chance of a future fracture
Other factors include:
your age, previous fracture, family history of fracture, use of alcohol, smoking, some other medicines, being physically inactive, having a fall…
Many people who have fractures don’t have low bone density – they don’t have “osteoporosis”
26

## Slide 27
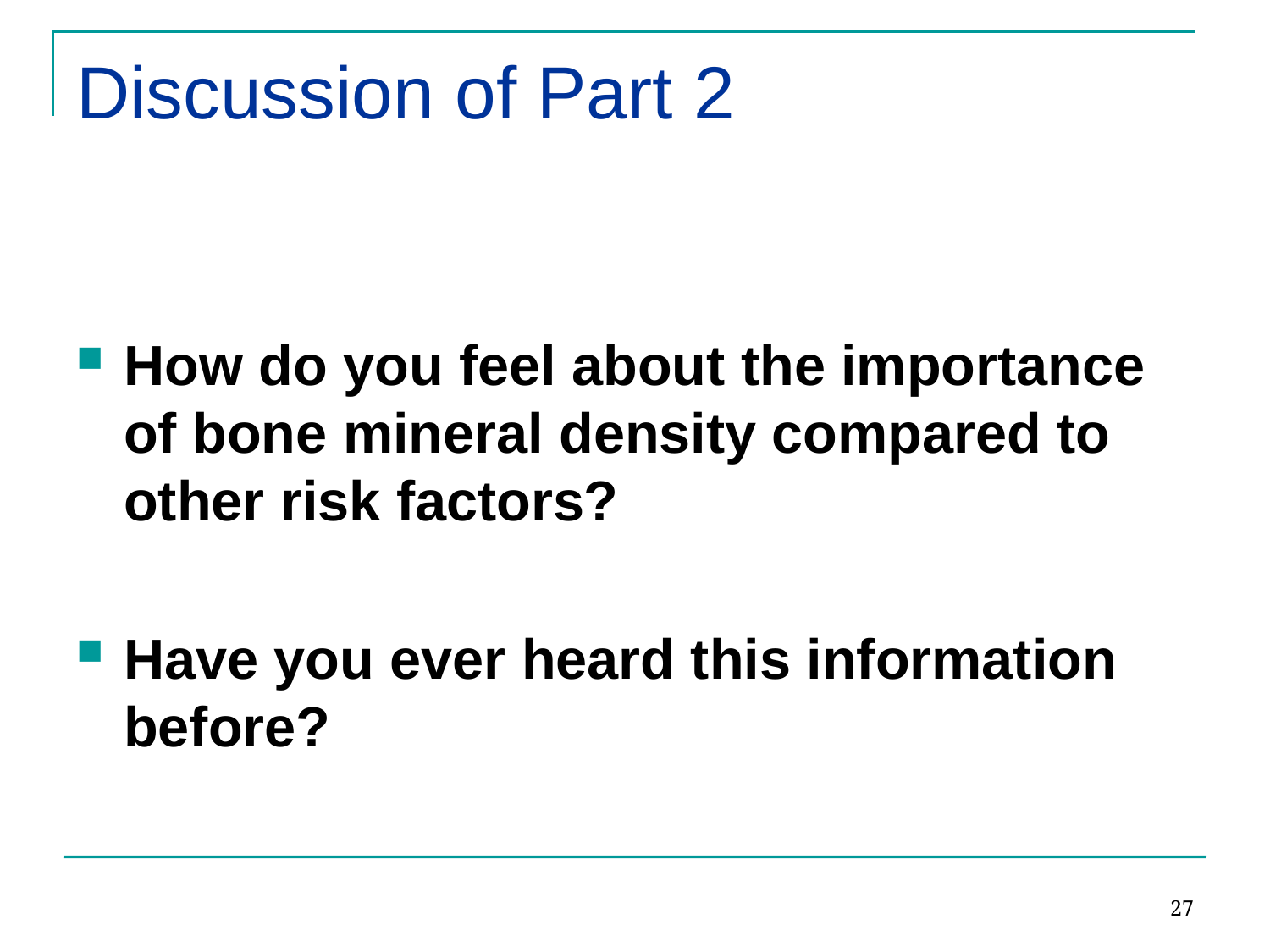

# Discussion of Part 2
How do you feel about the importance of bone mineral density compared to other risk factors?
Have you ever heard this information before?
27

## Slide 28
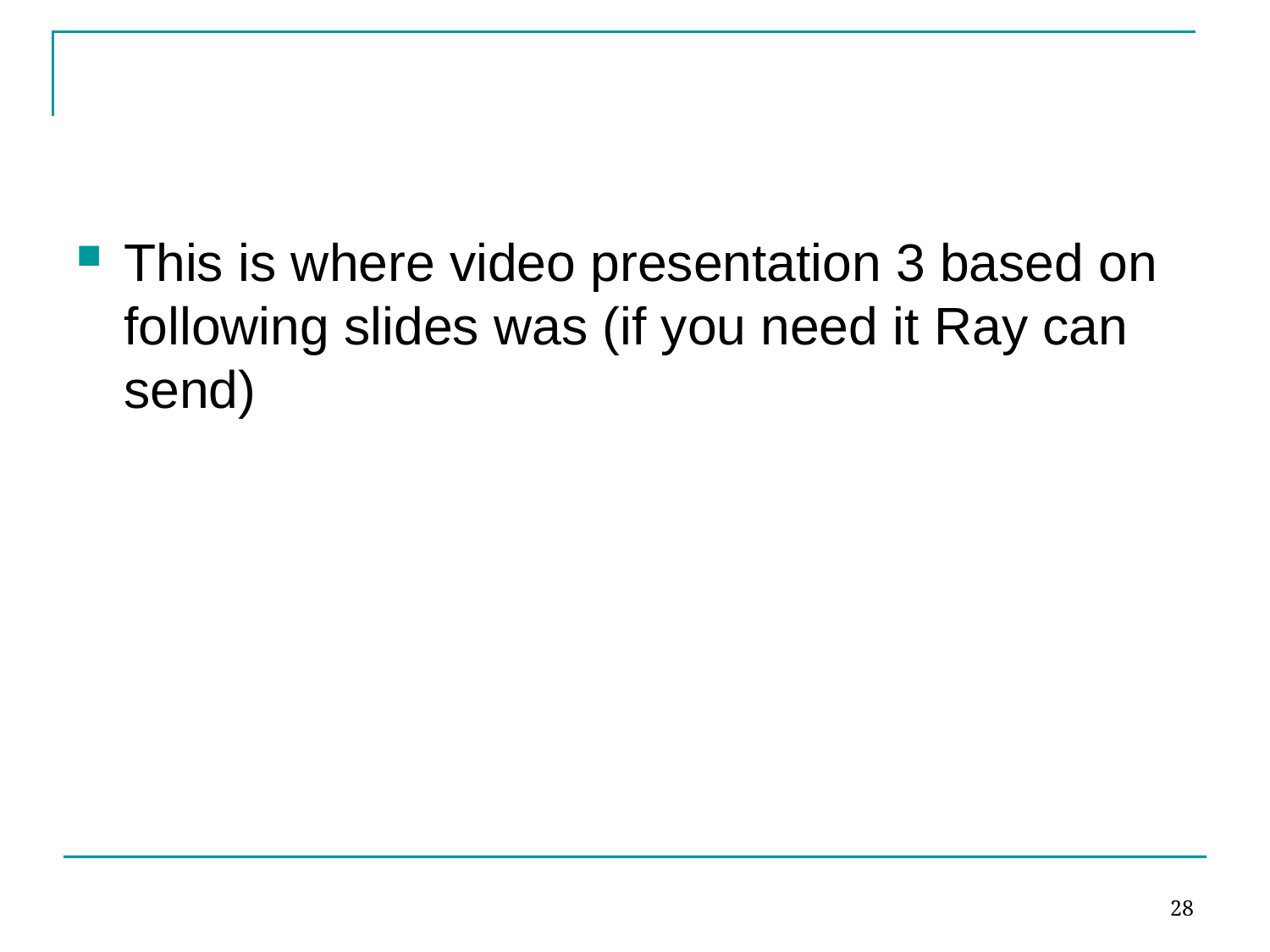

#
This is where video presentation 3 based on following slides was (if you need it Ray can send)
28

## Slide 29
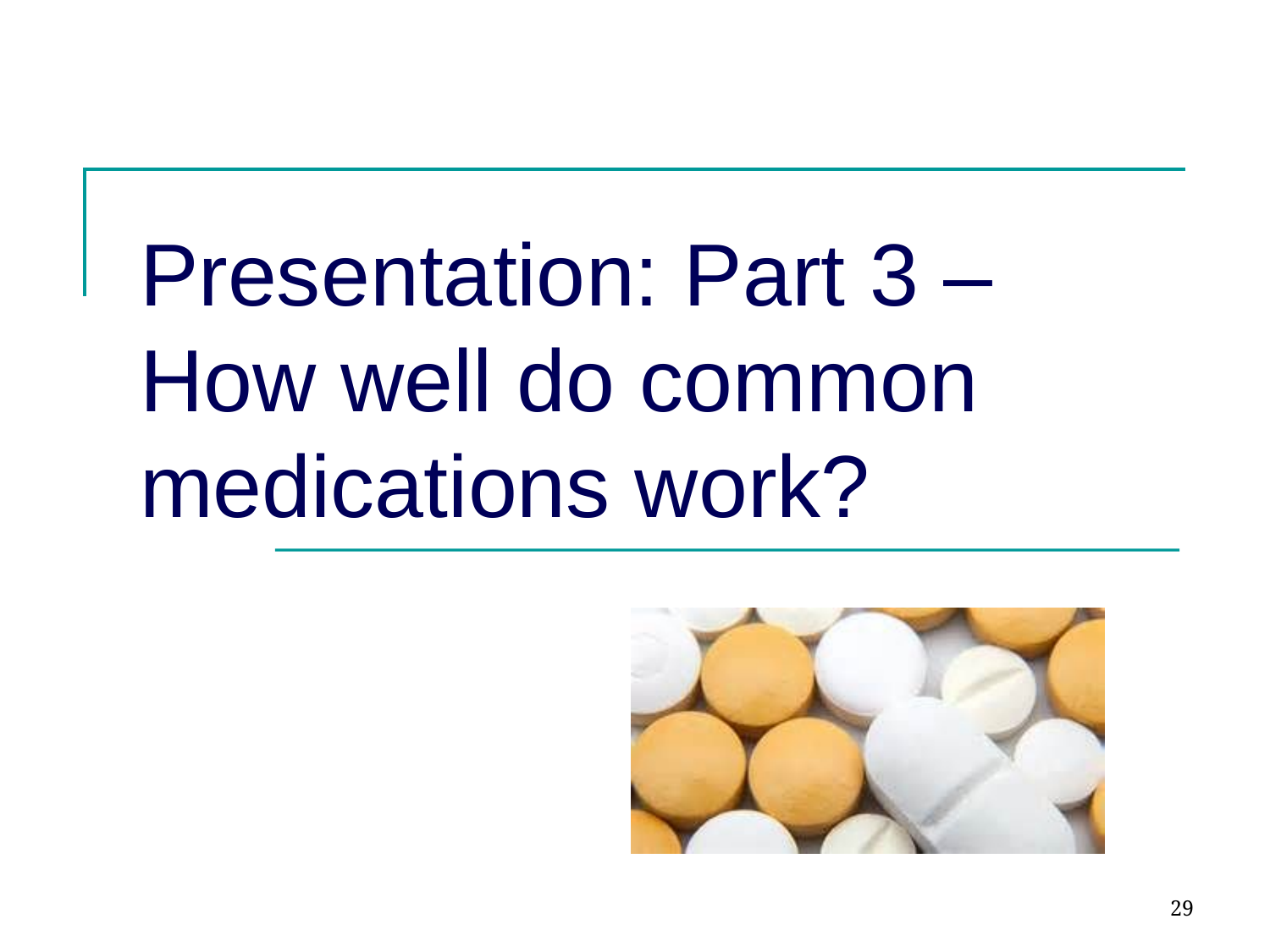

# Presentation: Part 3 – How well do common medications work?
29

## Slide 30
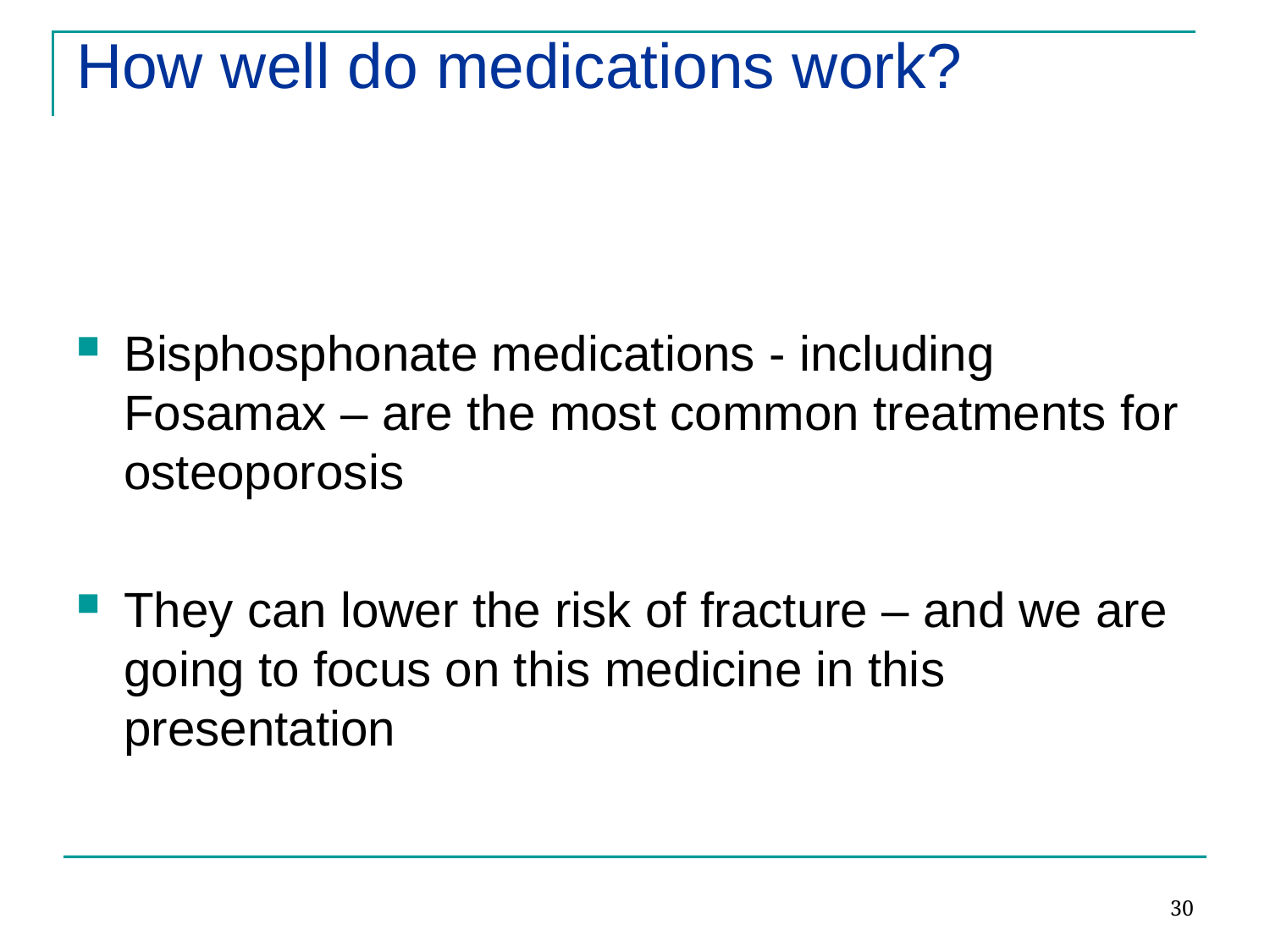

# How well do medications work?
Bisphosphonate medications - including Fosamax – are the most common treatments for osteoporosis
They can lower the risk of fracture – and we are going to focus on this medicine in this presentation
30

## Slide 31
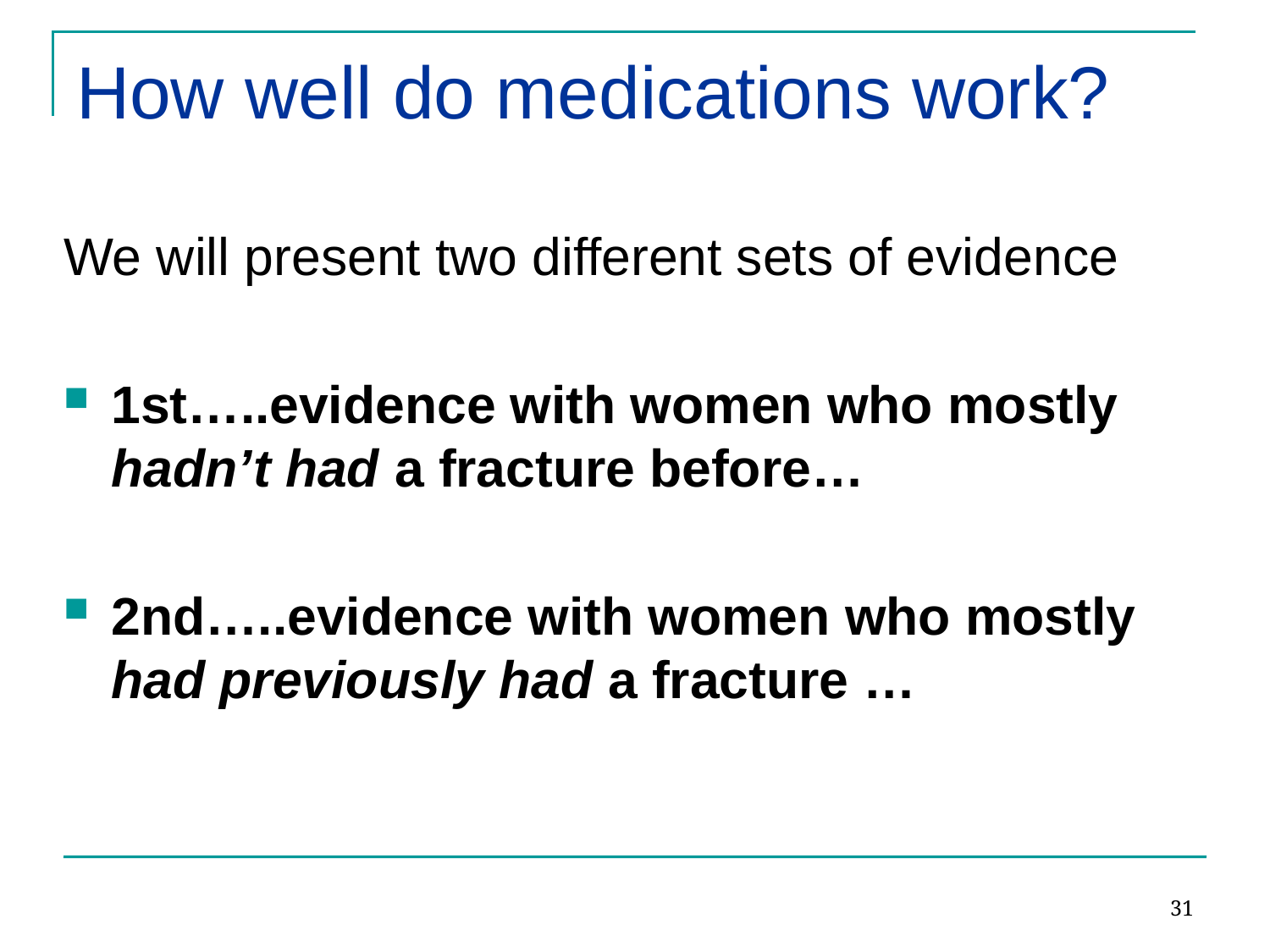

# How well do medications work?
We will present two different sets of evidence
1st…..evidence with women who mostly hadn’t had a fracture before…
2nd…..evidence with women who mostly had previously had a fracture …
31

## Slide 32
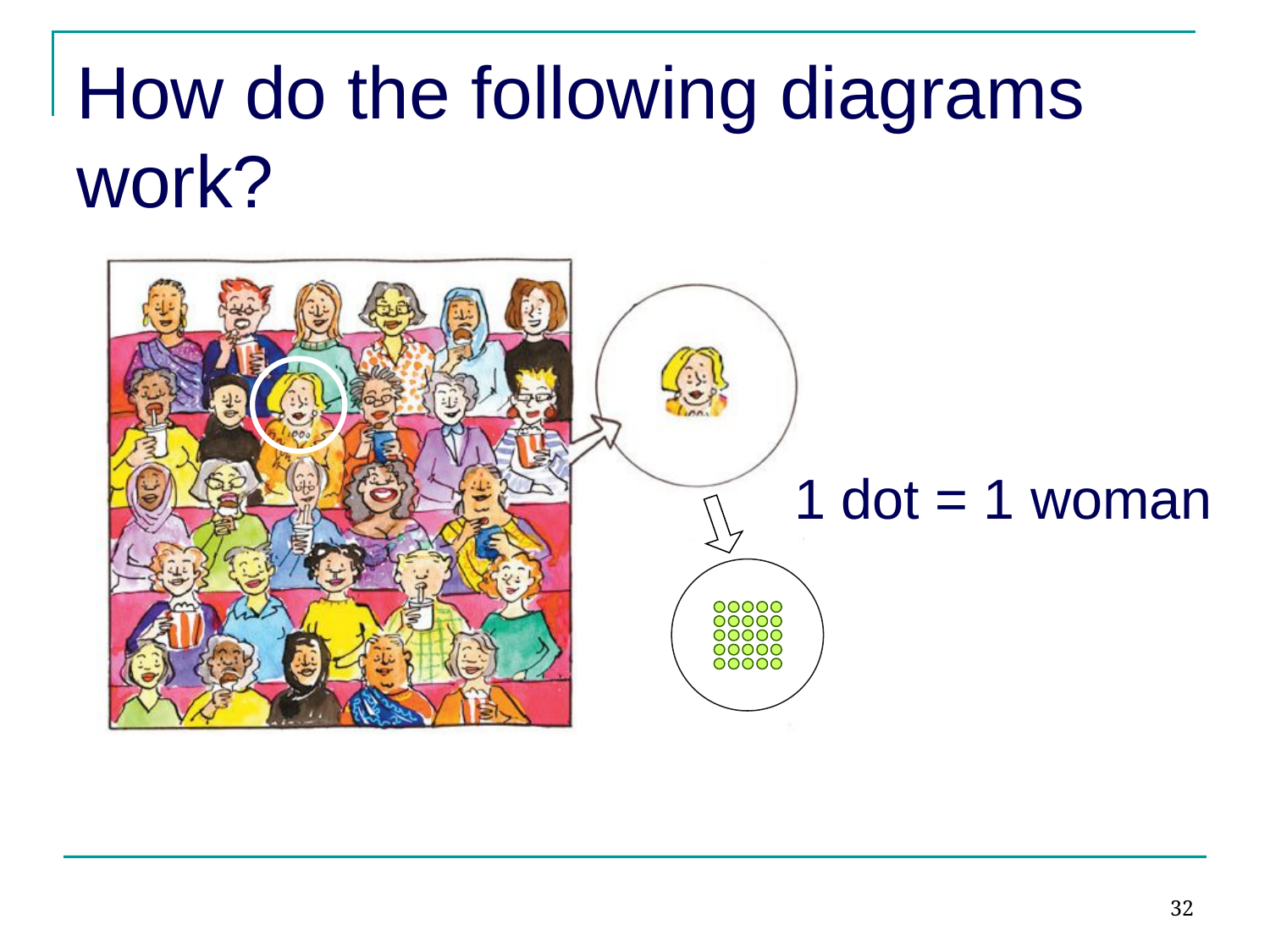

# How do the following diagrams work?
1 dot = 1 woman
32

## Slide 33
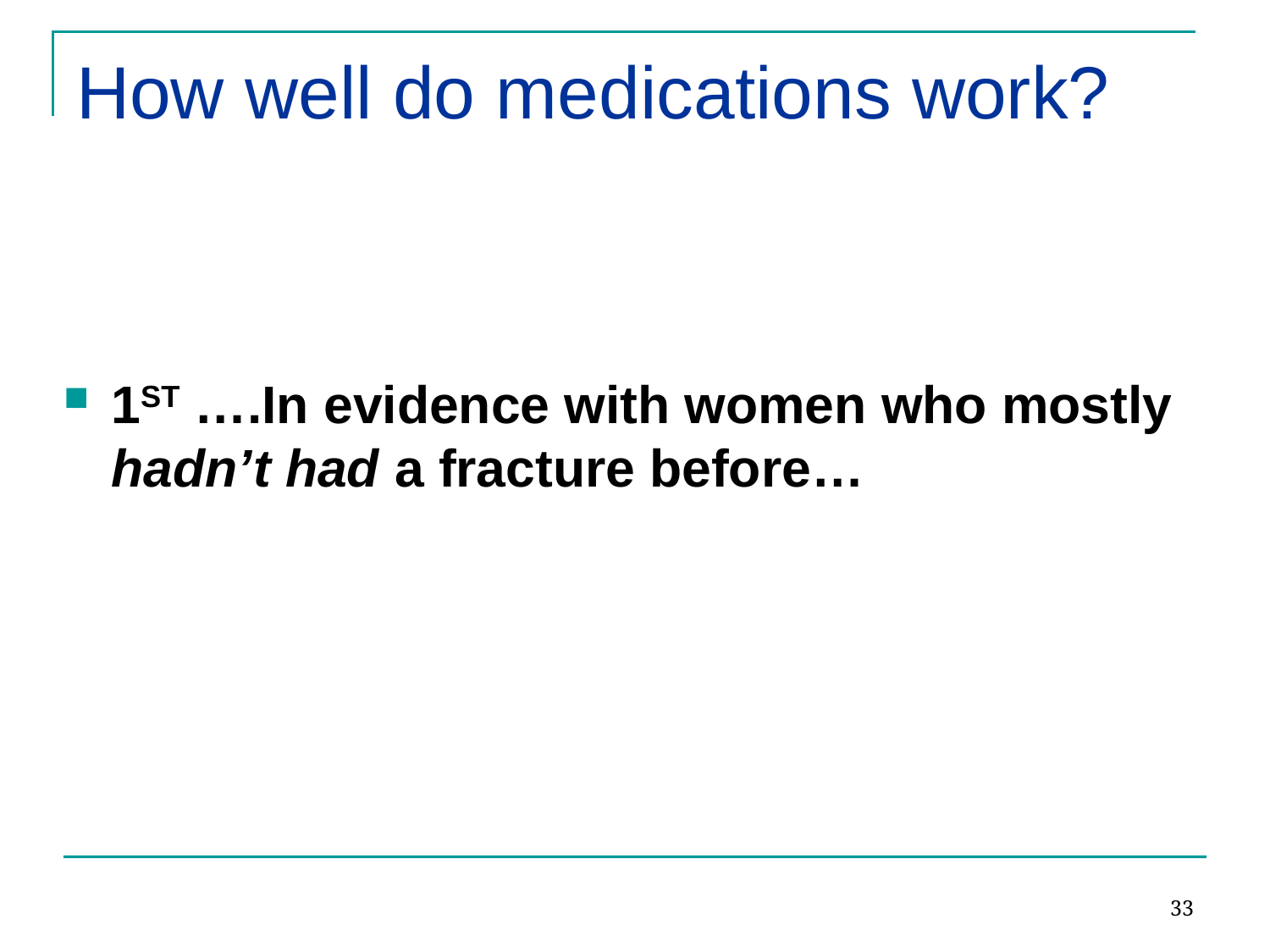

# How well do medications work?
1ST ….In evidence with women who mostly hadn’t had a fracture before…
33

## Slide 34
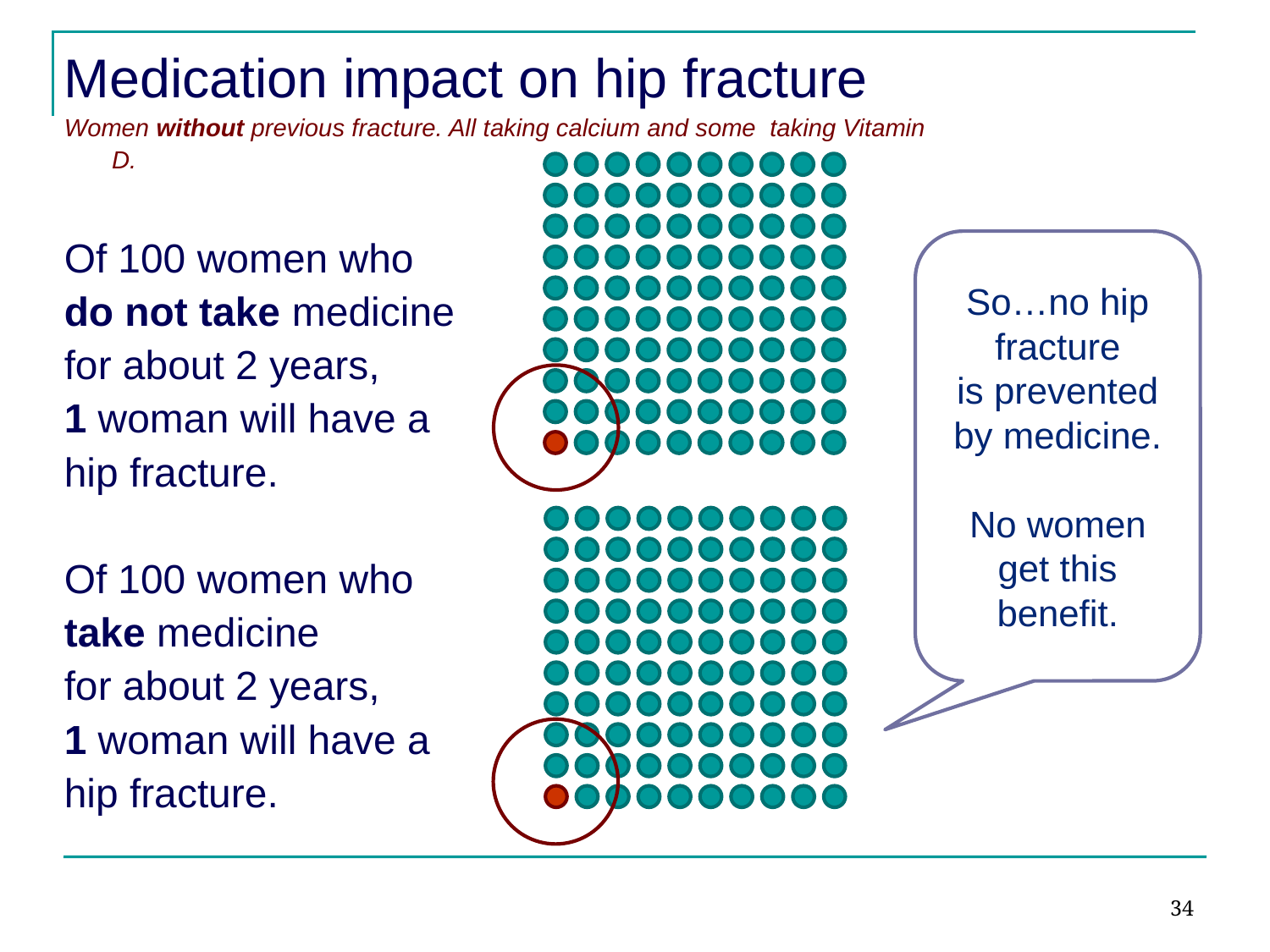

Medication impact on hip fracture
Women without previous fracture. All taking calcium and some taking Vitamin D.
Of 100 women who
do not take medicine
for about 2 years,
1 woman will have a
hip fracture.
Of 100 women who
take medicine
for about 2 years,
1 woman will have a
hip fracture.
So…no hip fracture
is prevented by medicine.
No women get this benefit.
34

## Slide 35
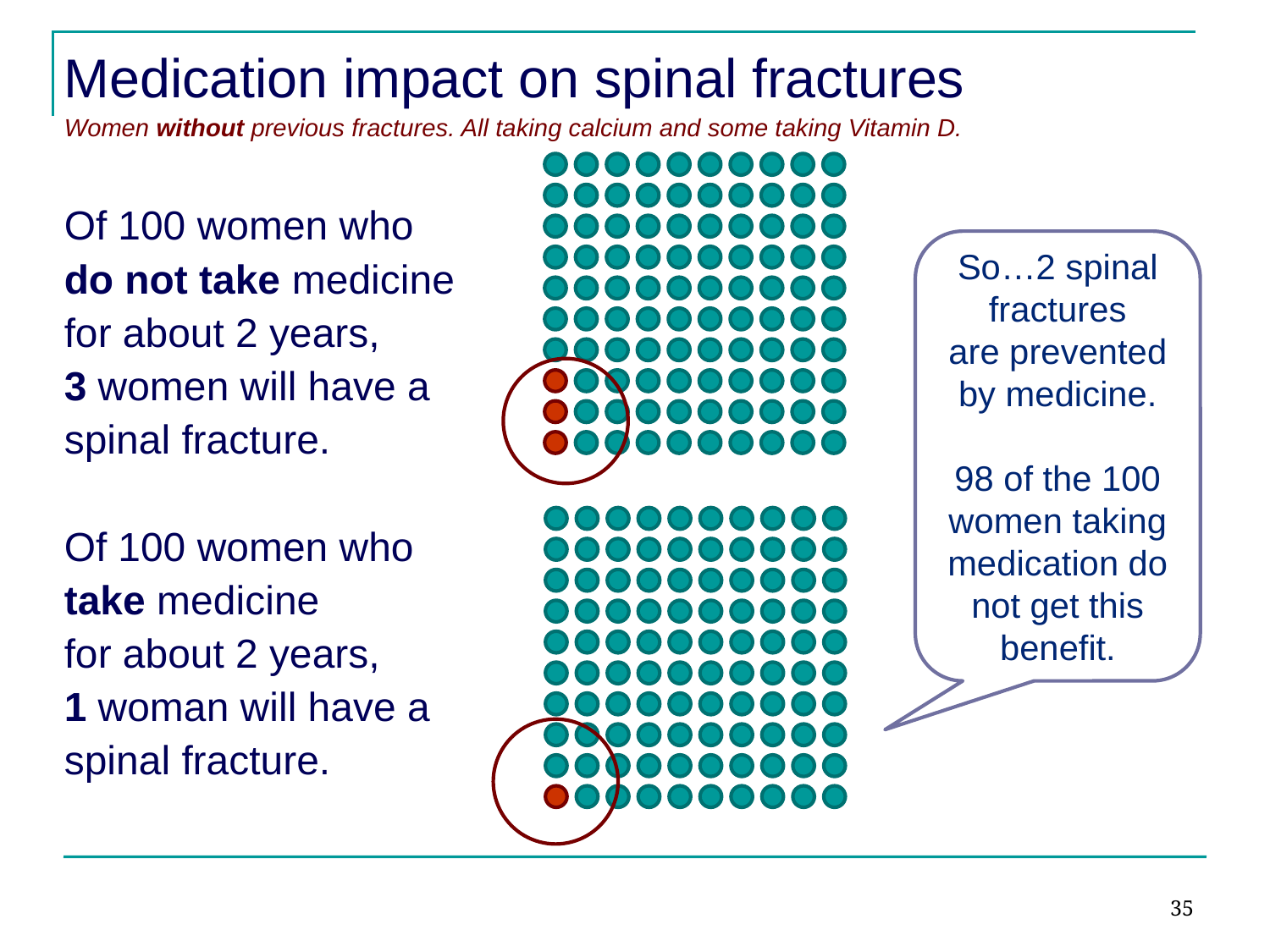

Medication impact on spinal fractures
Women without previous fractures. All taking calcium and some taking Vitamin D.
Of 100 women who
do not take medicine
for about 2 years,
3 women will have a
spinal fracture.
Of 100 women who
take medicine
for about 2 years,
1 woman will have a
spinal fracture.
So…2 spinal fractures
are prevented by medicine.
98 of the 100 women taking medication do not get this benefit.
35

## Slide 36
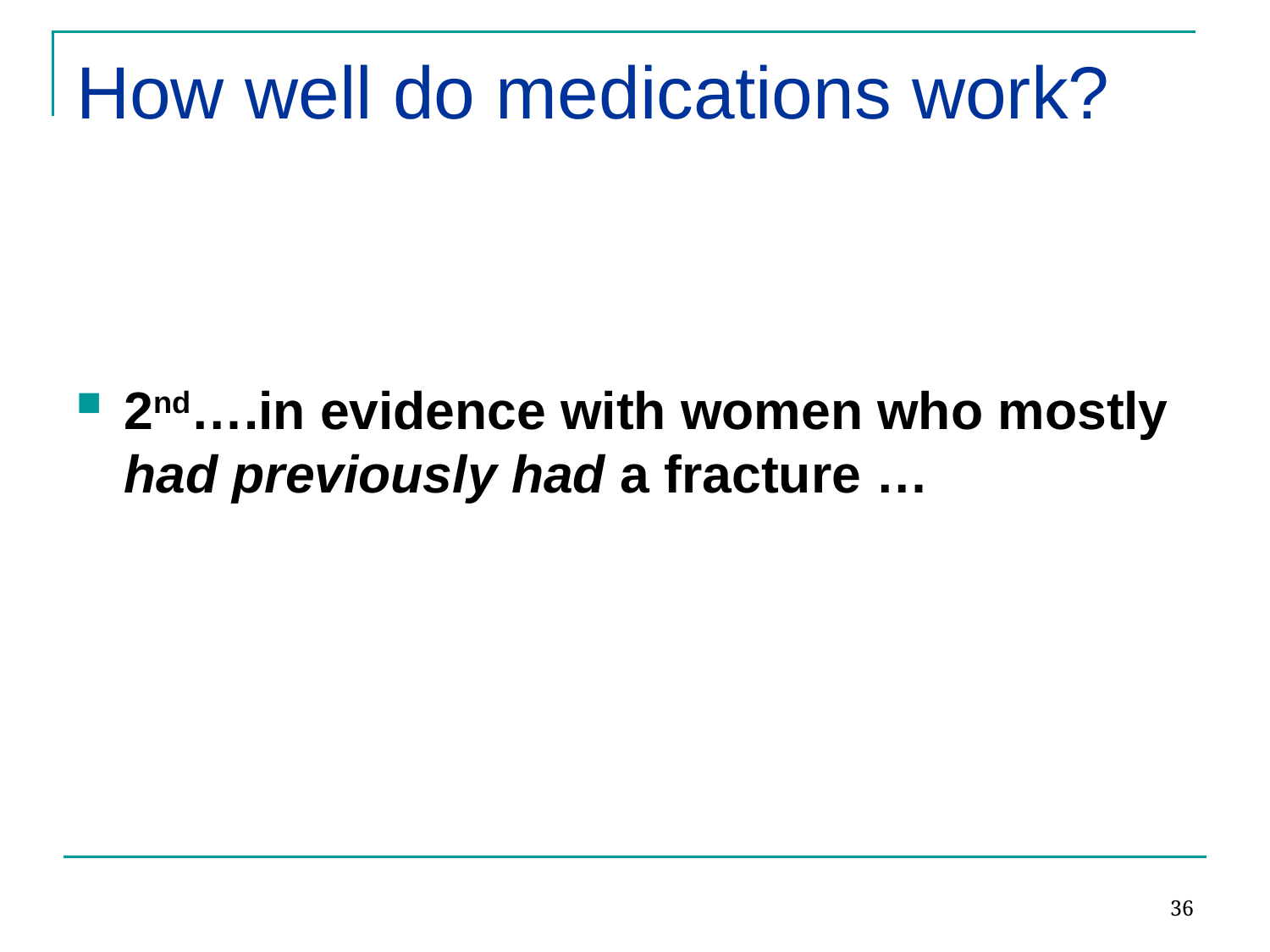

# How well do medications work?
2nd….in evidence with women who mostly had previously had a fracture …
36

## Slide 37
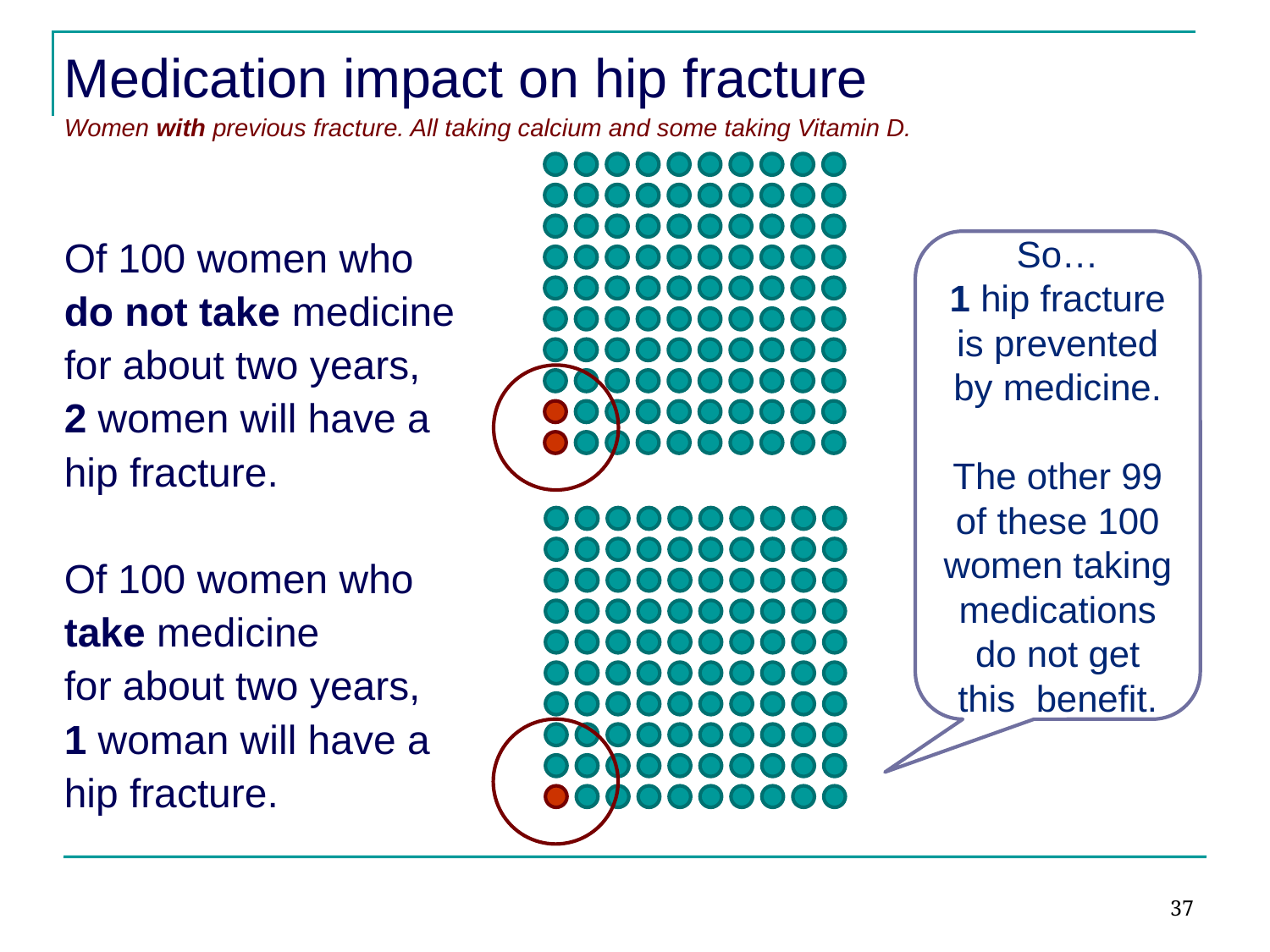

Medication impact on hip fracture
Women with previous fracture. All taking calcium and some taking Vitamin D.
Of 100 women who
do not take medicine
for about two years,
2 women will have a
hip fracture.
Of 100 women who
take medicine
for about two years,
1 woman will have a
hip fracture.
So…
1 hip fracture
is prevented by medicine.
The other 99 of these 100 women taking medications do not get this benefit.
37

## Slide 38
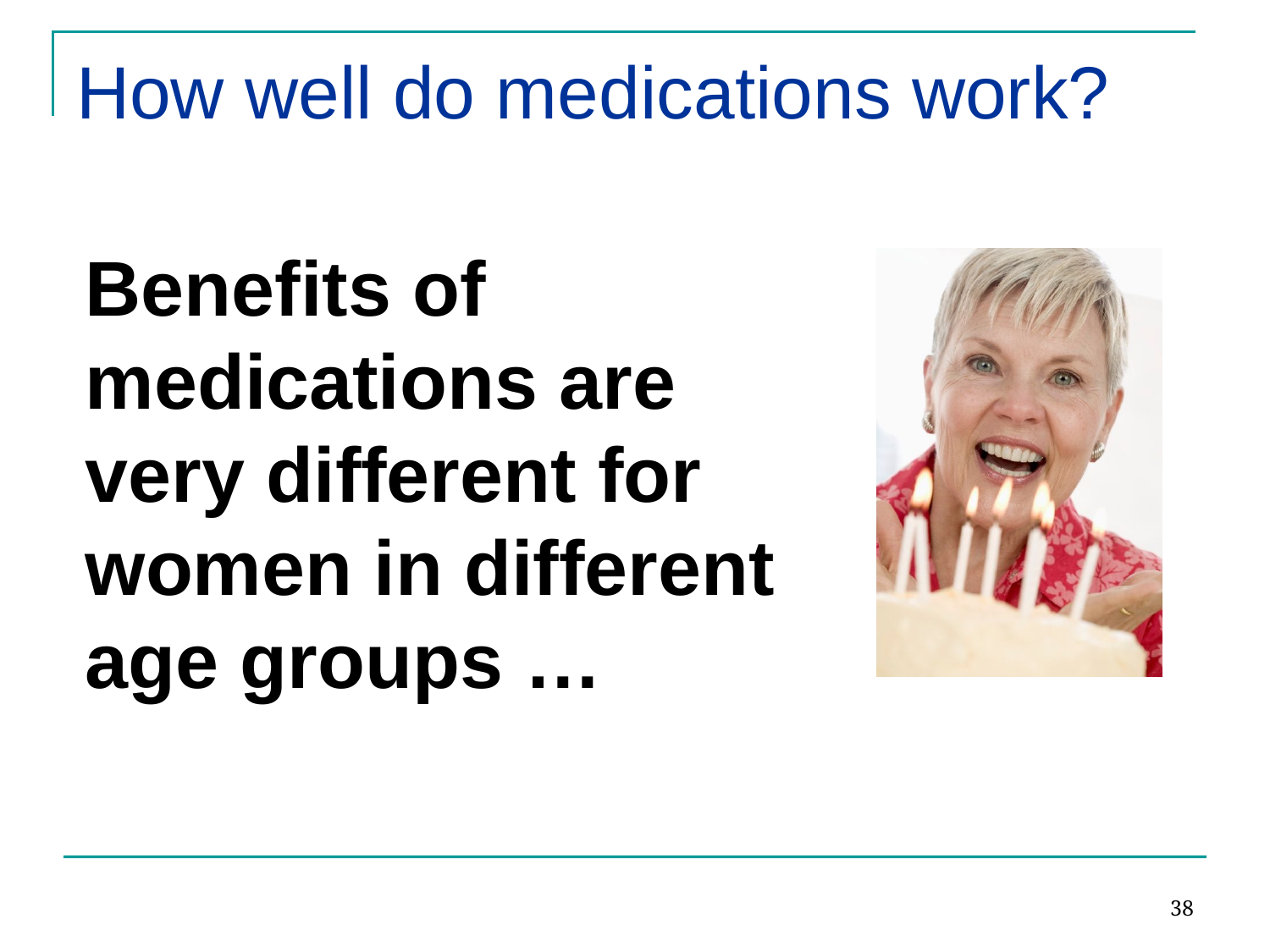

# How well do medications work?
Benefits of medications are very different for women in different age groups …
38

## Slide 39
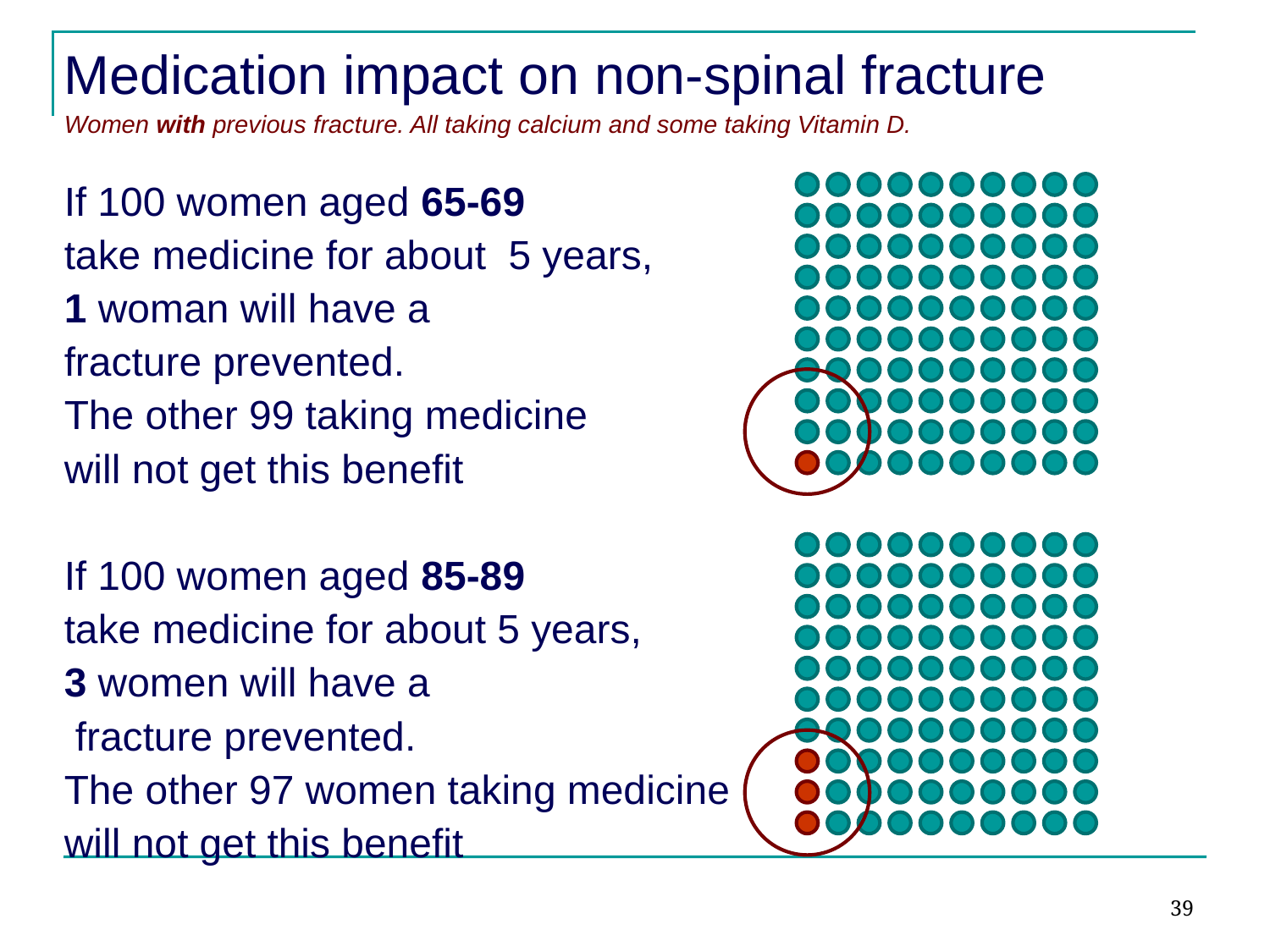

Medication impact on non-spinal fracture
Women with previous fracture. All taking calcium and some taking Vitamin D.
If 100 women aged 65-69
take medicine for about 5 years,
1 woman will have a
fracture prevented.
The other 99 taking medicine
will not get this benefit
If 100 women aged 85-89
take medicine for about 5 years,
3 women will have a
 fracture prevented.
The other 97 women taking medicine
will not get this benefit
39

## Slide 40
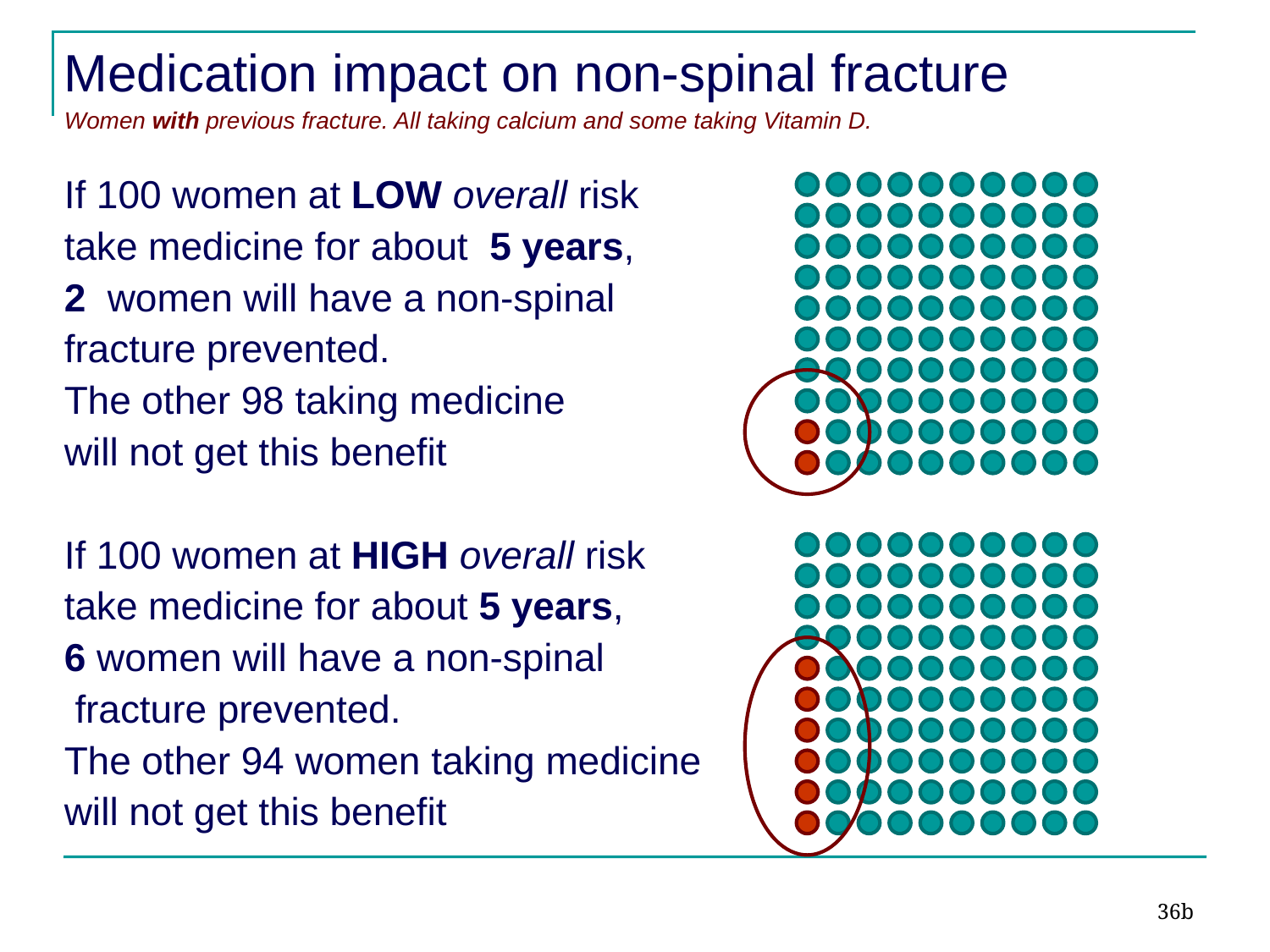

Medication impact on non-spinal fracture
Women with previous fracture. All taking calcium and some taking Vitamin D.
If 100 women at LOW overall risk
take medicine for about 5 years,
2 women will have a non-spinal
fracture prevented.
The other 98 taking medicine
will not get this benefit
If 100 women at HIGH overall risk
take medicine for about 5 years,
6 women will have a non-spinal
 fracture prevented.
The other 94 women taking medicine
will not get this benefit
36b

## Slide 41
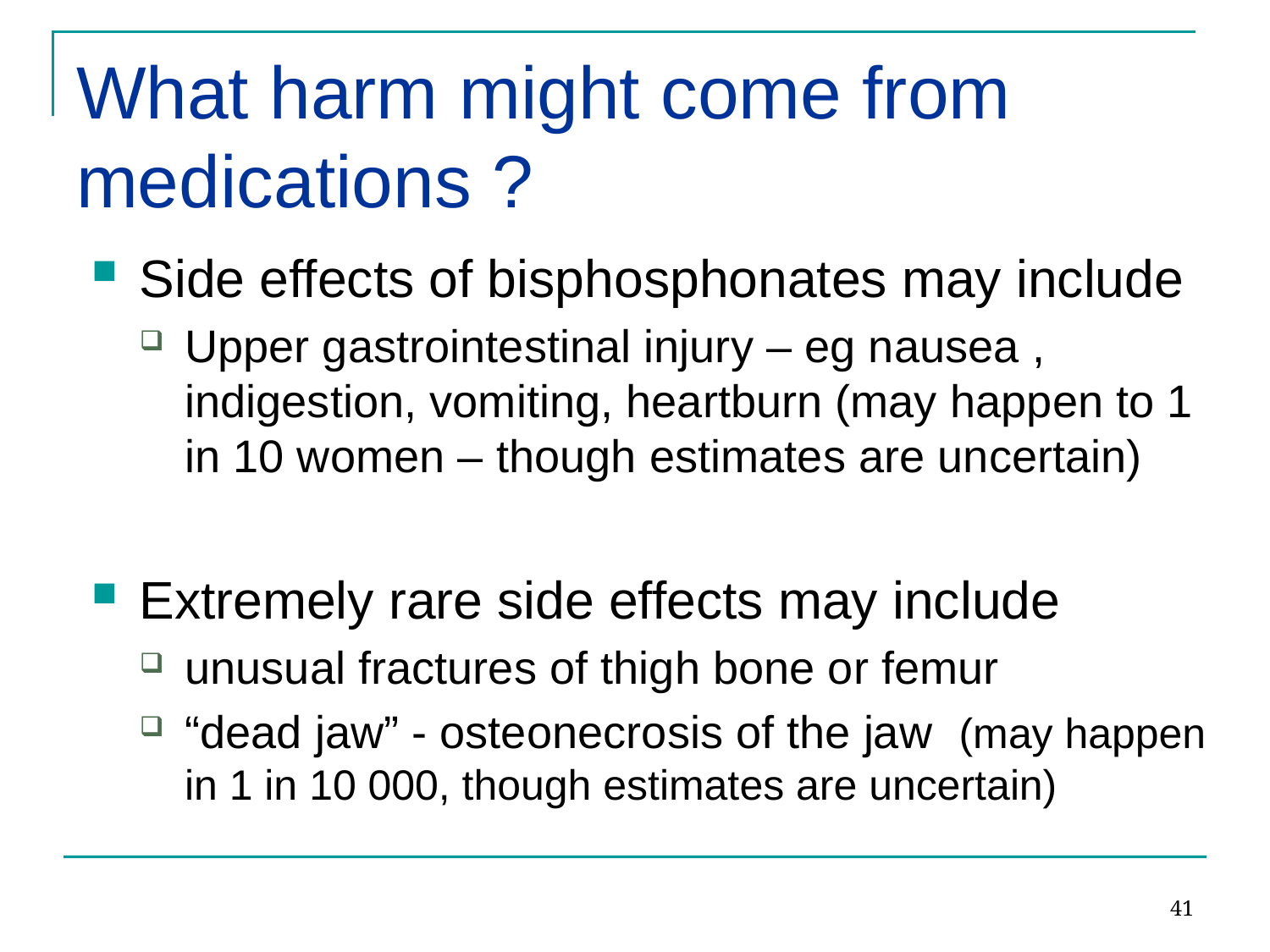

# What harm might come from medications ?
Side effects of bisphosphonates may include
Upper gastrointestinal injury – eg nausea , indigestion, vomiting, heartburn (may happen to 1 in 10 women – though estimates are uncertain)
Extremely rare side effects may include
unusual fractures of thigh bone or femur
“dead jaw” - osteonecrosis of the jaw (may happen in 1 in 10 000, though estimates are uncertain)
41

## Slide 42
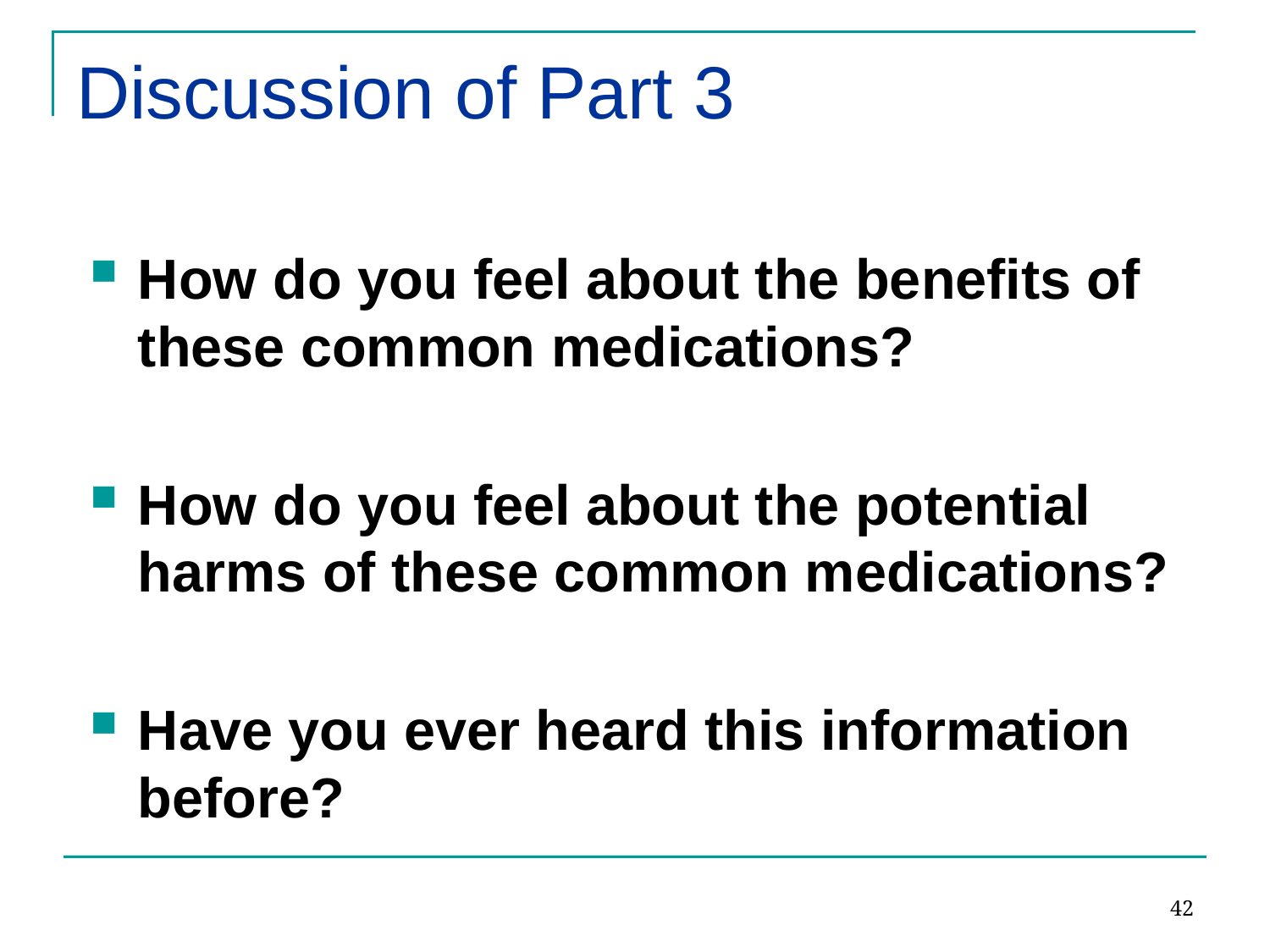

# Discussion of Part 3
How do you feel about the benefits of these common medications?
How do you feel about the potential harms of these common medications?
Have you ever heard this information before?
42

## Slide 43
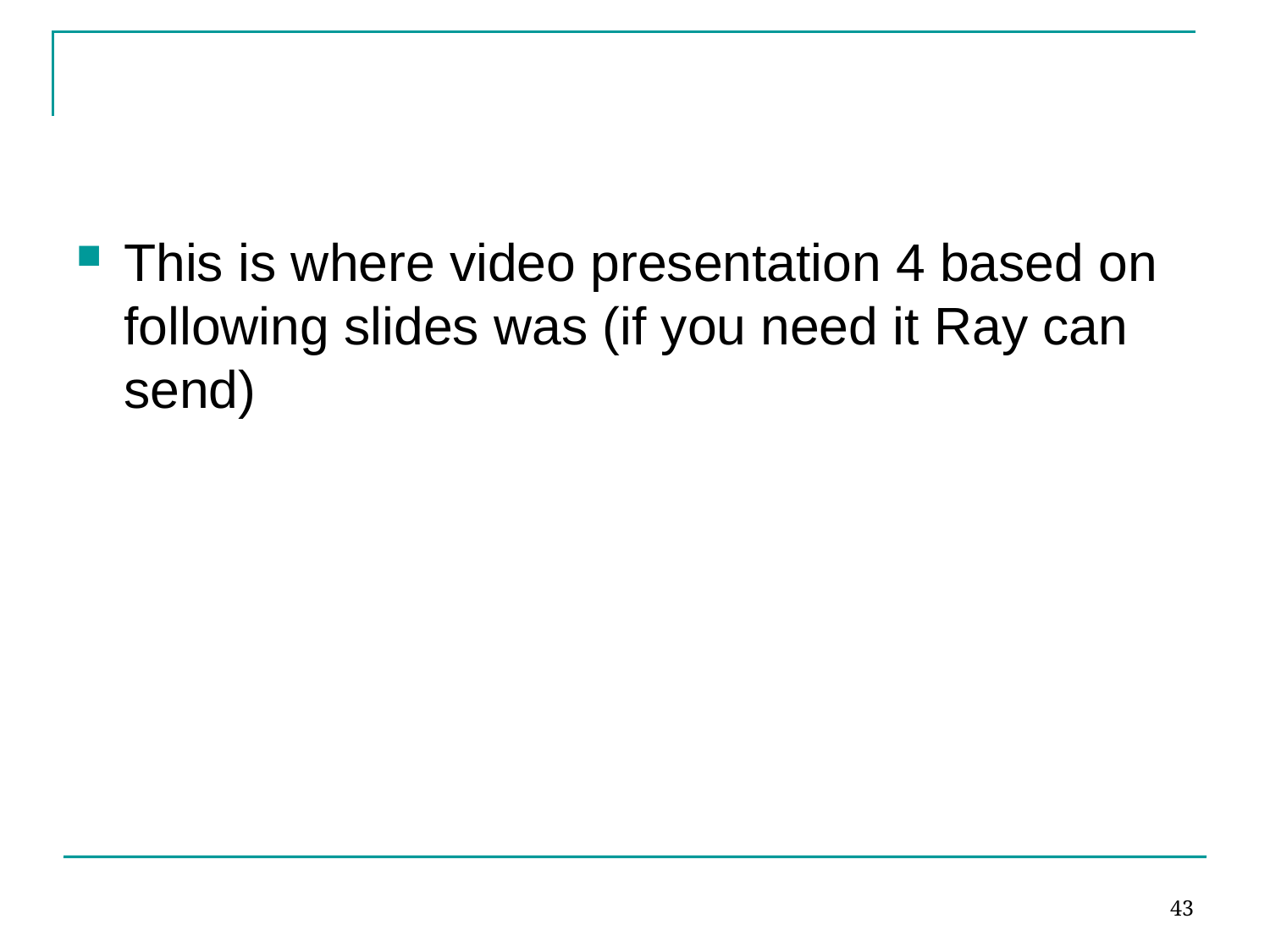

#
This is where video presentation 4 based on following slides was (if you need it Ray can send)
43

## Slide 44
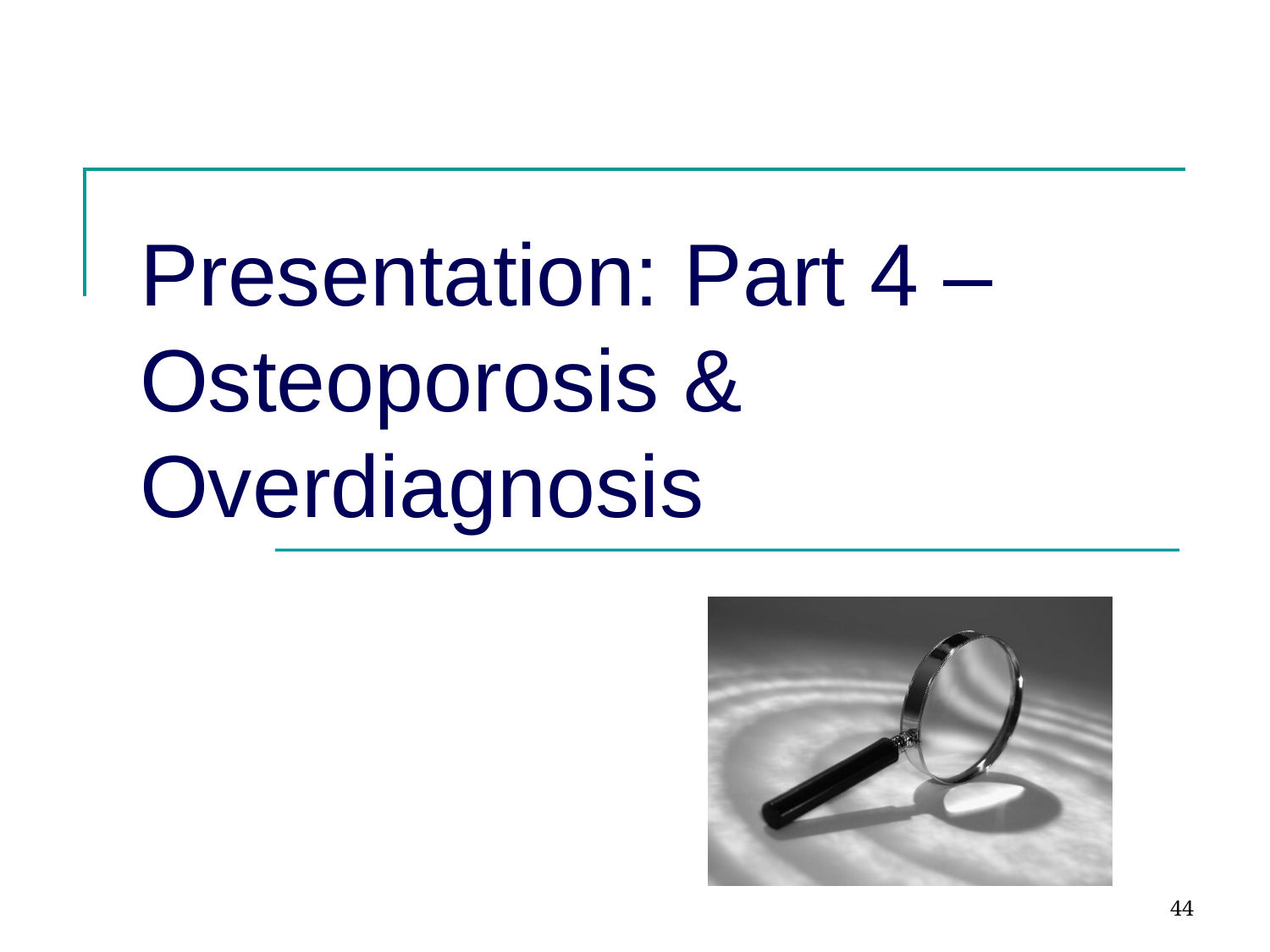

# Presentation: Part 4 –Osteoporosis & Overdiagnosis
44

## Slide 45
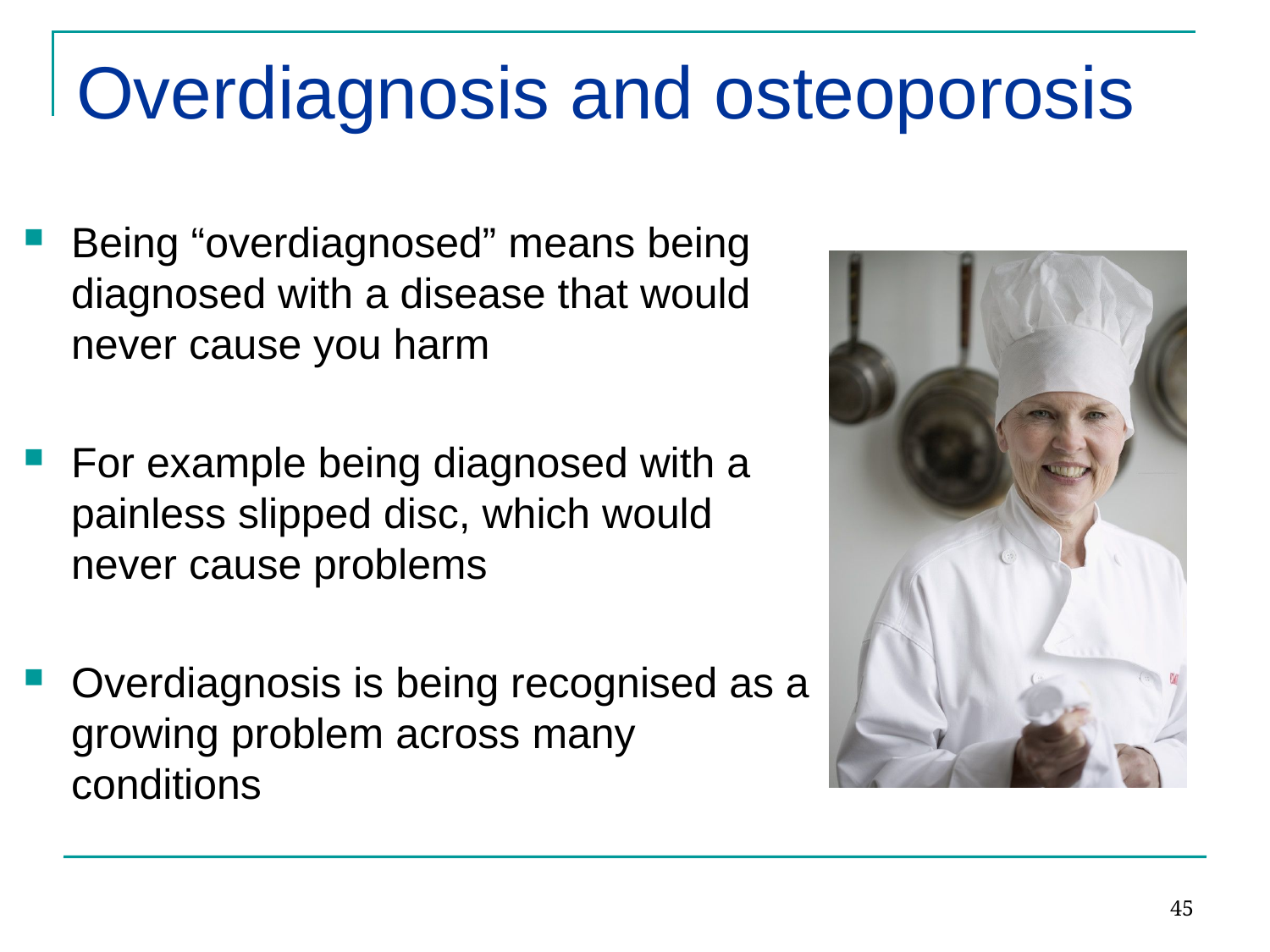

Being “overdiagnosed” means being diagnosed with a disease that would never cause you harm
For example being diagnosed with a painless slipped disc, which would never cause problems
Overdiagnosis is being recognised as a growing problem across many conditions
# Overdiagnosis and osteoporosis
45

## Slide 46
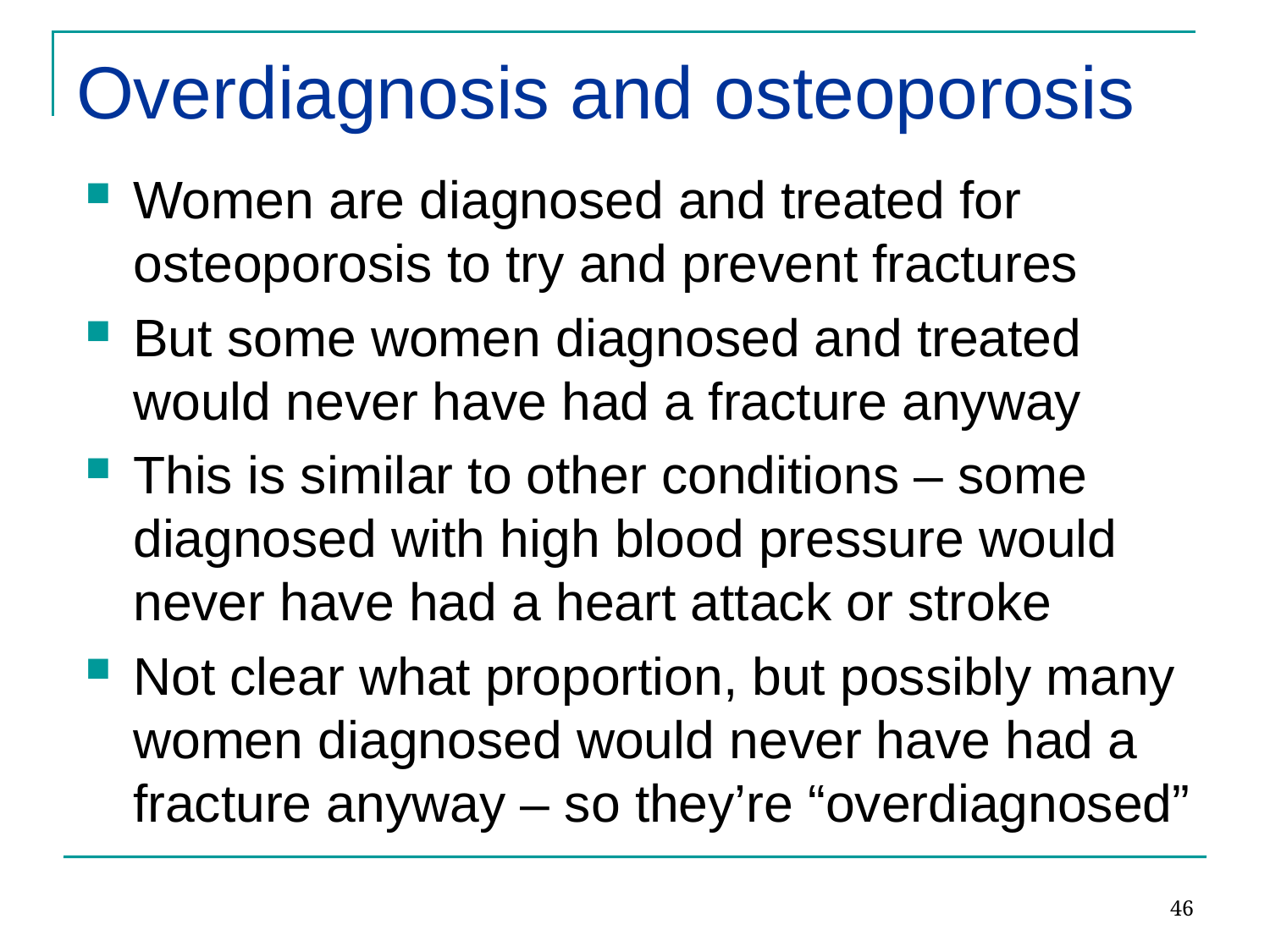

# Overdiagnosis and osteoporosis
Women are diagnosed and treated for osteoporosis to try and prevent fractures
But some women diagnosed and treated would never have had a fracture anyway
This is similar to other conditions – some diagnosed with high blood pressure would never have had a heart attack or stroke
Not clear what proportion, but possibly many women diagnosed would never have had a fracture anyway – so they’re “overdiagnosed”
46

## Slide 47
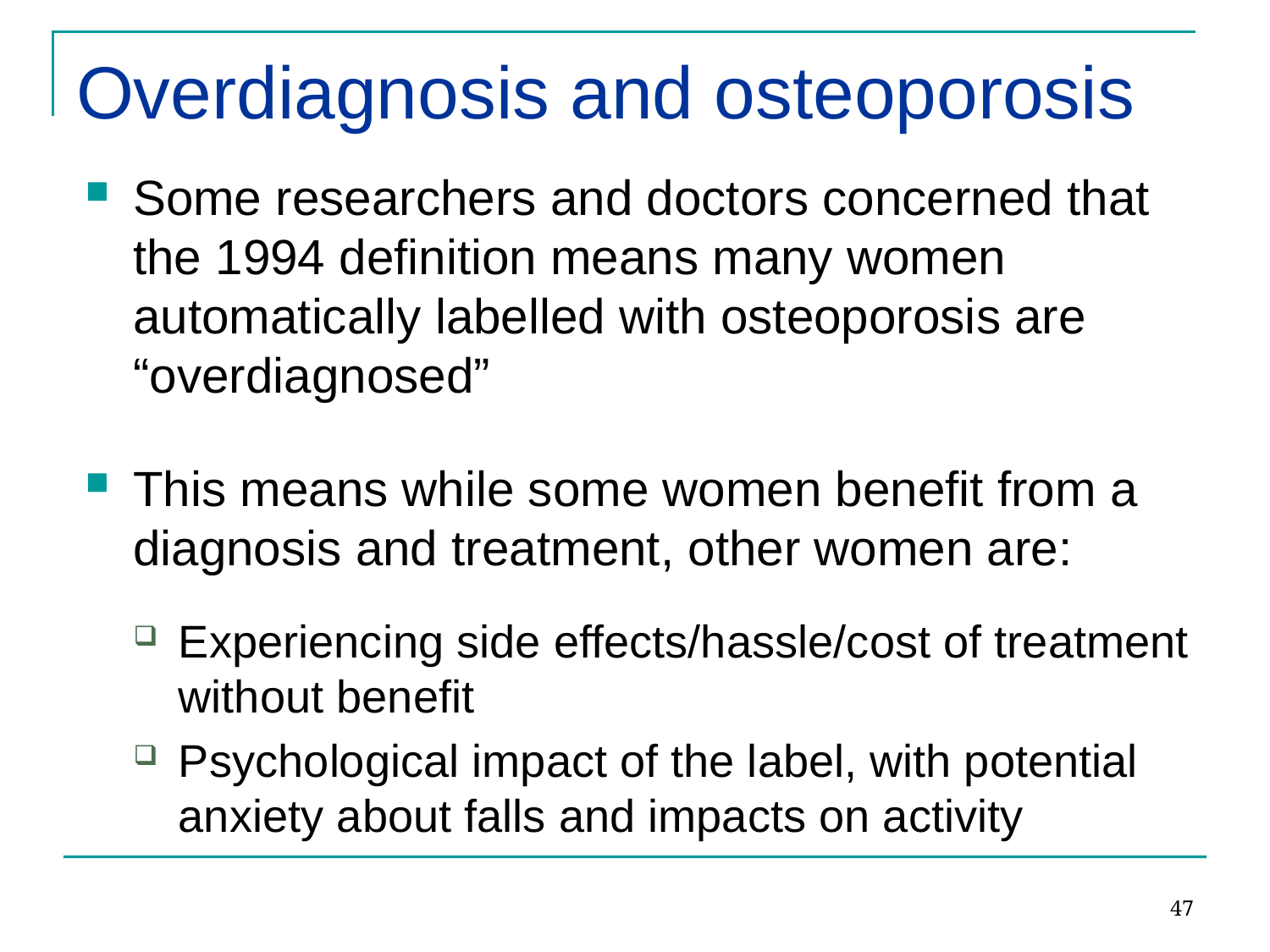

# Overdiagnosis and osteoporosis
Some researchers and doctors concerned that the 1994 definition means many women automatically labelled with osteoporosis are “overdiagnosed”
This means while some women benefit from a diagnosis and treatment, other women are:
Experiencing side effects/hassle/cost of treatment without benefit
Psychological impact of the label, with potential anxiety about falls and impacts on activity
47

## Slide 48
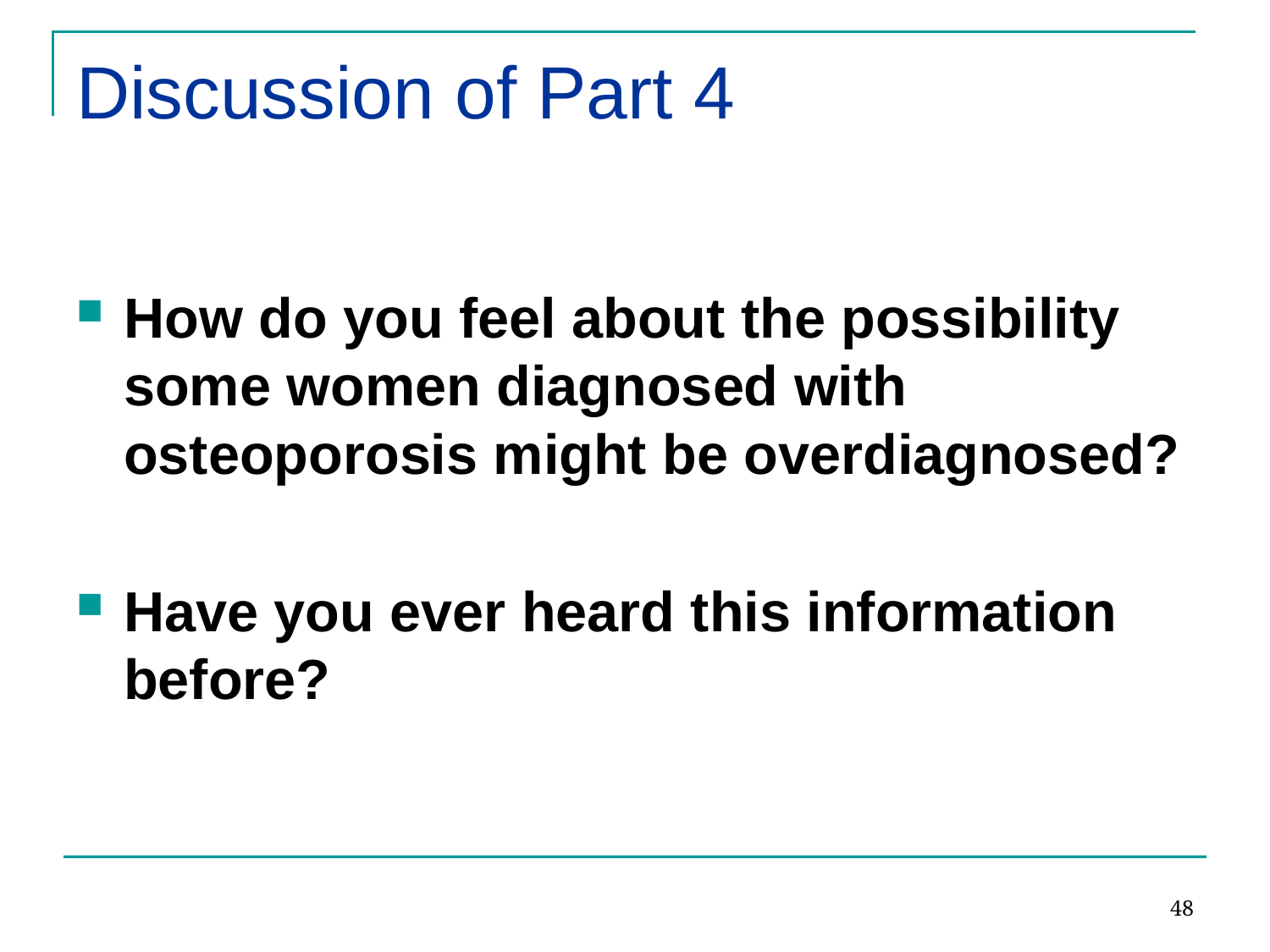

# Discussion of Part 4
How do you feel about the possibility some women diagnosed with osteoporosis might be overdiagnosed?
Have you ever heard this information before?
48

## Slide 49
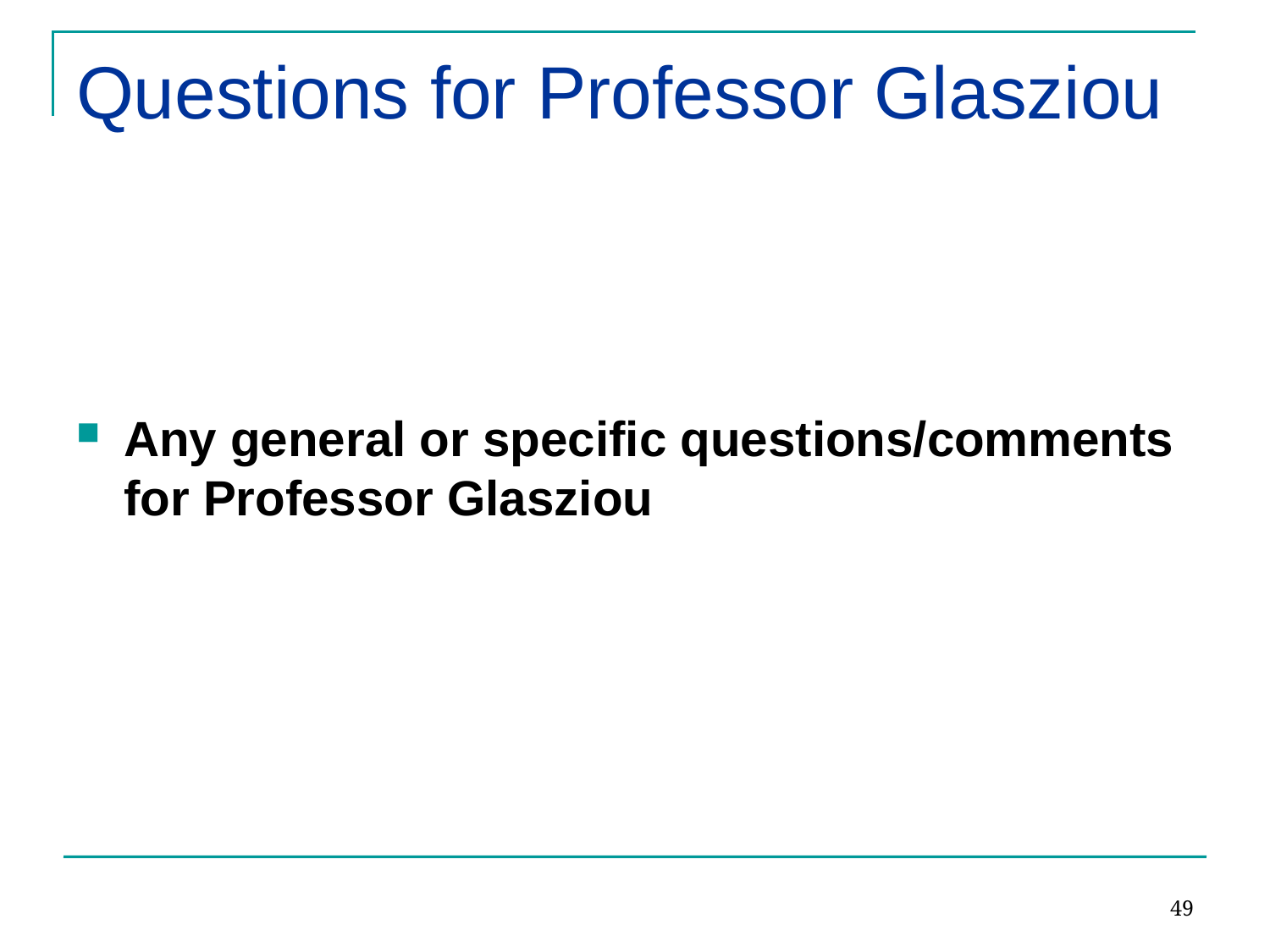

# Questions for Professor Glasziou
Any general or specific questions/comments for Professor Glasziou
49

## Slide 50
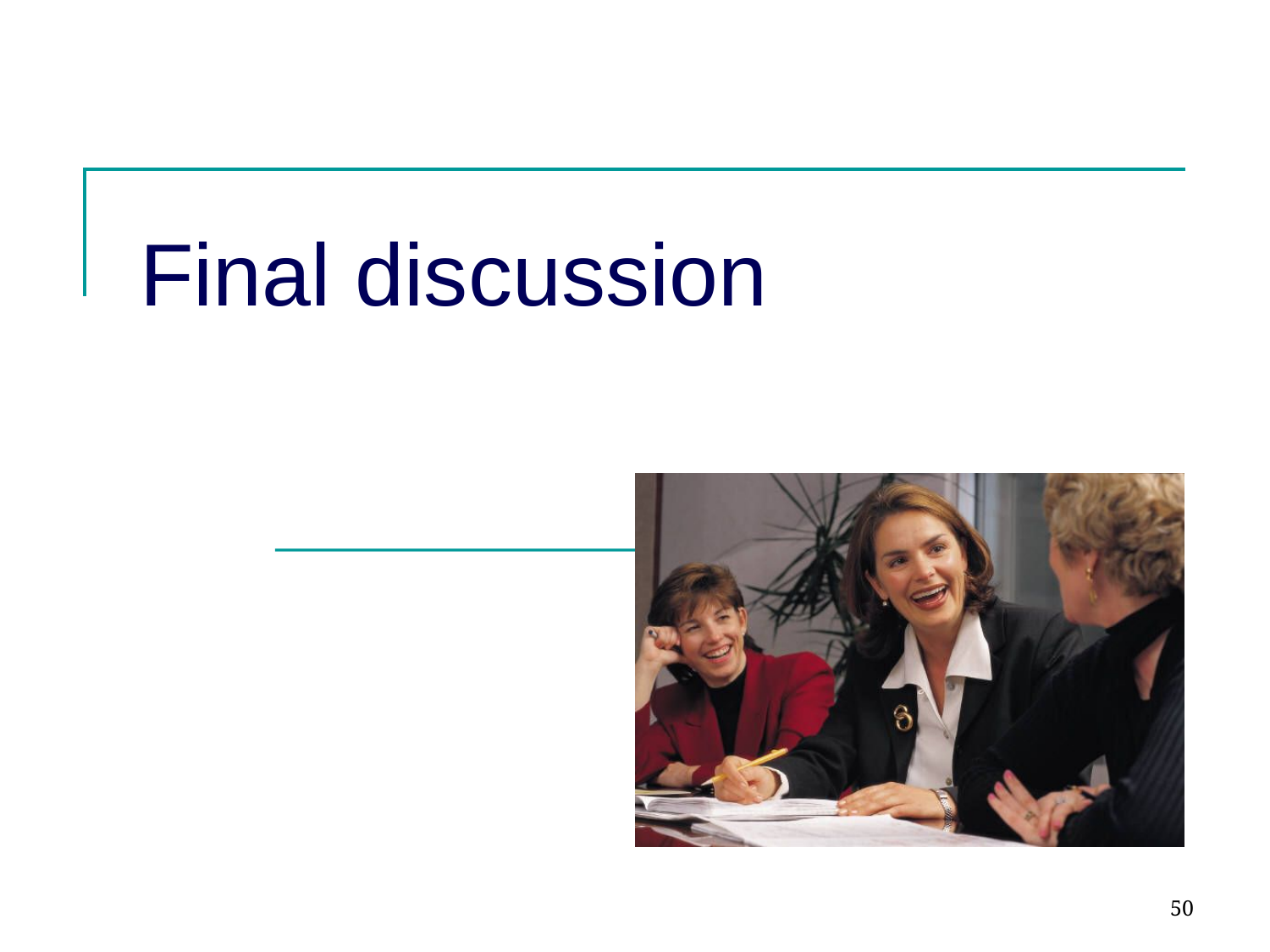

# Final discussion
50

## Slide 51
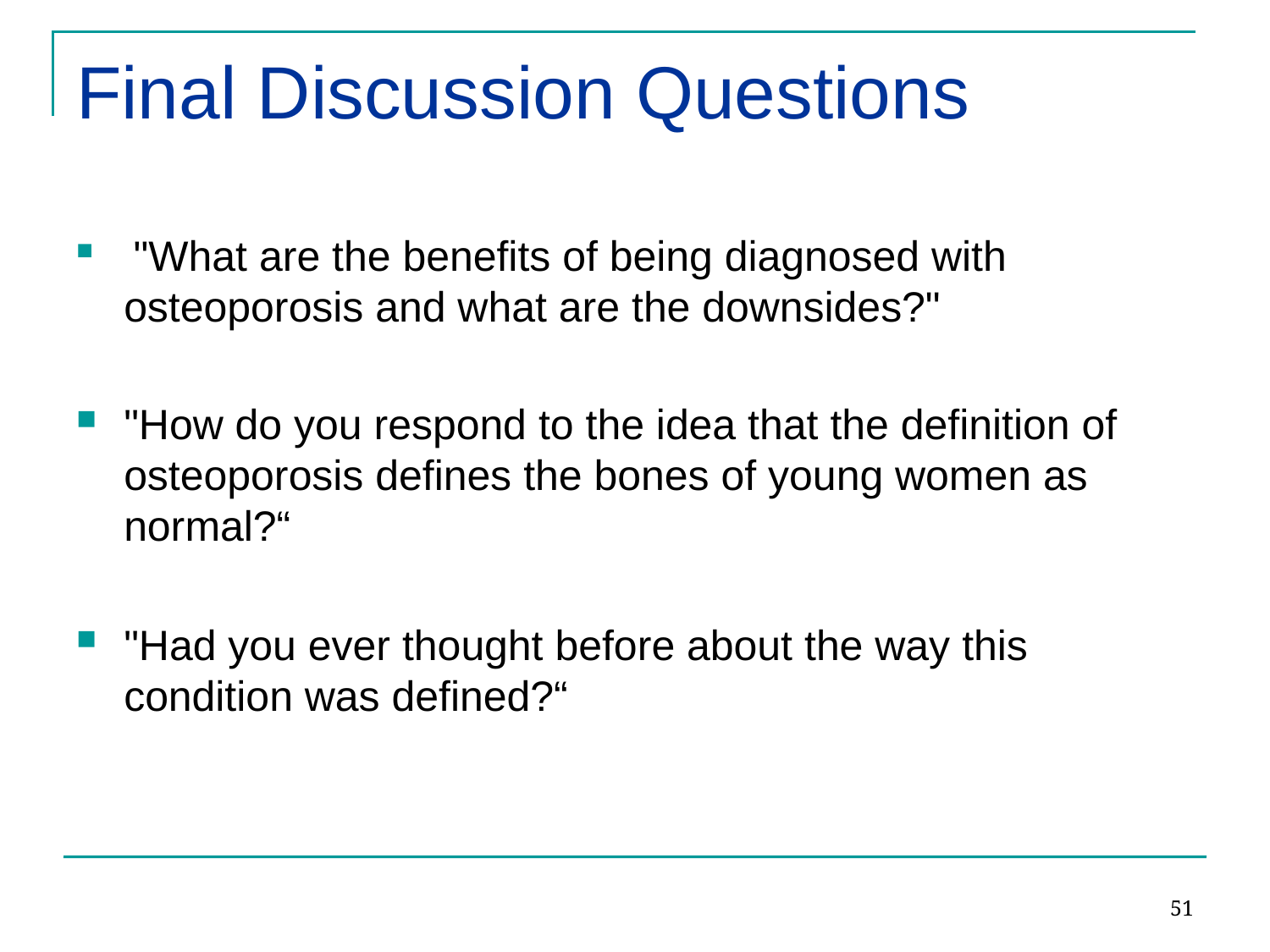

# Final Discussion Questions
 "What are the benefits of being diagnosed with osteoporosis and what are the downsides?"
"How do you respond to the idea that the definition of osteoporosis defines the bones of young women as normal?“
"Had you ever thought before about the way this condition was defined?“
51

## Slide 52
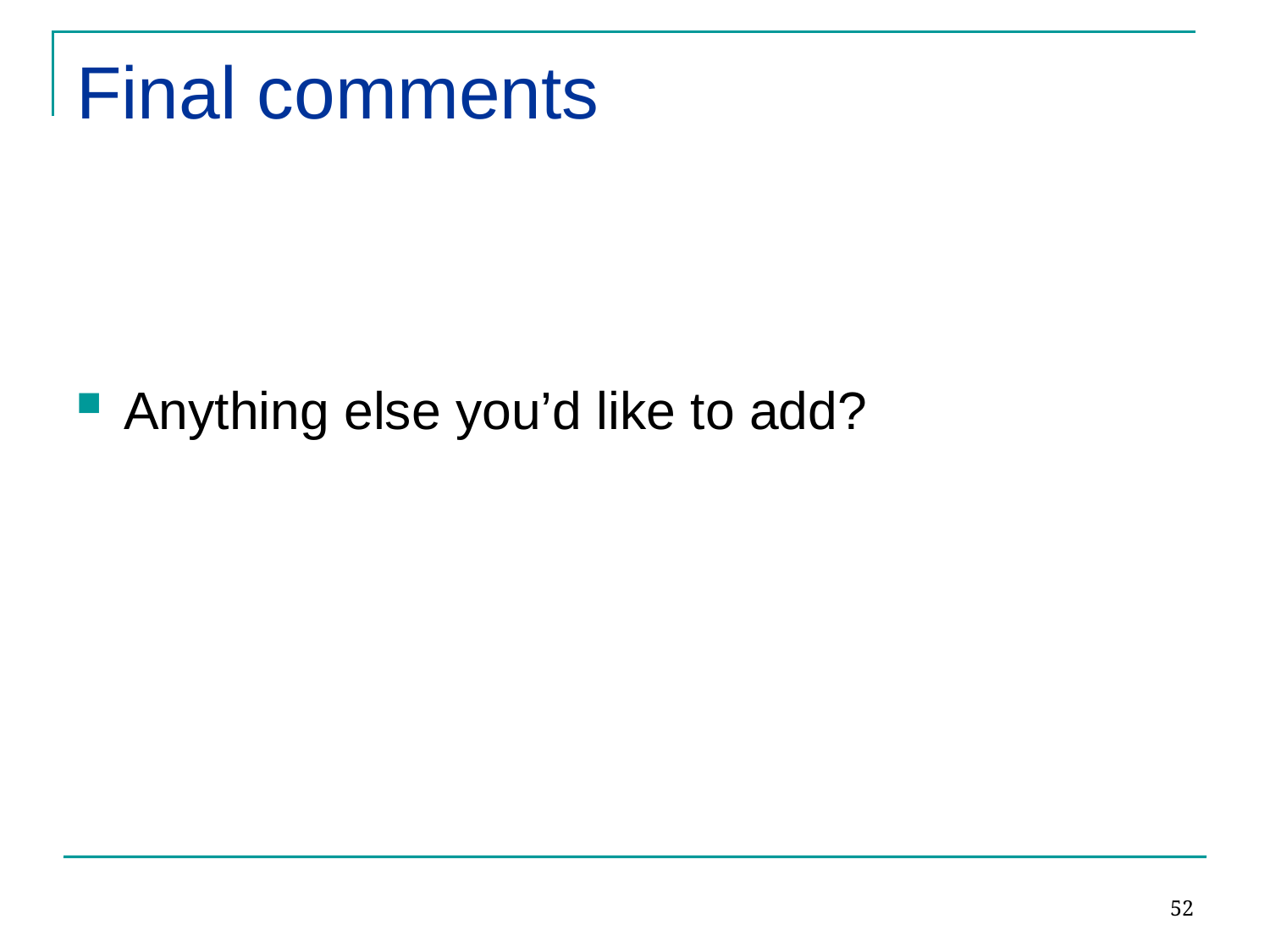

# Final comments
Anything else you’d like to add?
52

## Slide 53
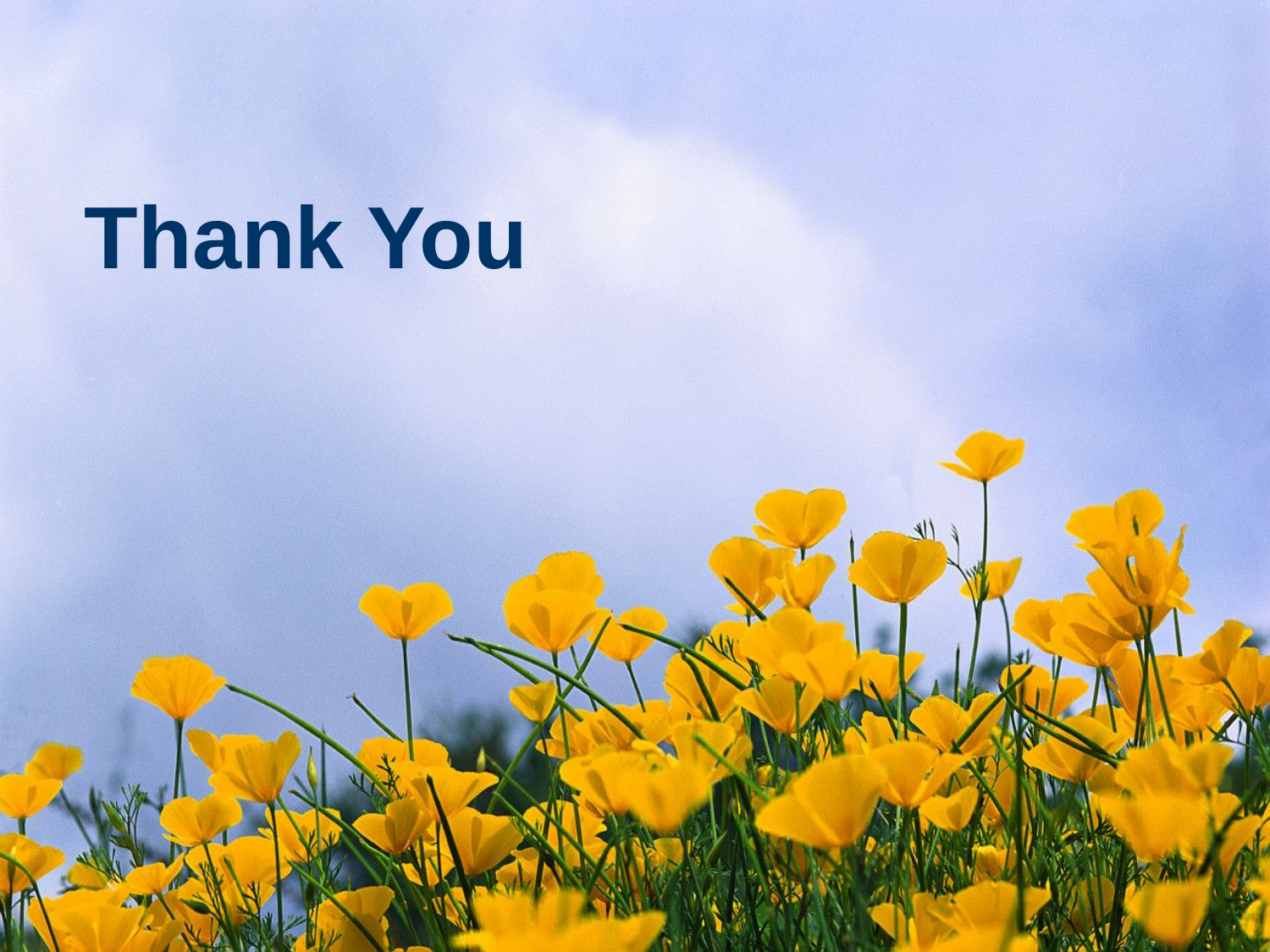

# Thank You
53
