# Supplementary material for: Communicating about overdiagnosis: Learning from community focus groups on osteoporosis
Source: PLoS One. 2017 Feb 3;12(2):e0170142. doi: 10.1371/journal.pone.0170142 (PMC5291414; doi:10.1371/journal.pone.0170142)
Supplement: S4 Text — (PDF) [file pone.0170142.s004.pdf]

TRANSCRIPT

Audio Name: *Osteo Presentations - Professor Paul Glasziou*

Audio Length: *15 minutes*

Start of Transcript

[Section 1]

Paul Glasziou: Good morning. I'm Paul Glasziou. I'm a general practitioner and a clinical researcher and I'm particularly interested in screening. I want to talk to you a little bit about what is osteoporosis.

So first, a little bit of history about osteoporosis. If we go back a century, osteoporosis was a diagnosis that was limited to people who had had painful spinal fractures, so they had vertebra like this in your back but those had been crushed. Sometimes those are pain free but sometimes they're painful and they can cause that stooped posture and severe curvature that you can see in some old people, but it was pretty rare.

Then in the 1970s and 1980s, we developed new devices that could allow the study of bone change over the age, so this allowed us to see osteoporosis long before we got to the crush fractures and stooped spines that we have in some patients. The problem was, we needed to come up with a definition and so for a couple of decades people hadn't decided where exactly to draw the line.

In 1994, a WHO, World Health Organisation, study group decided to define bone density of young women as being normal. So the usual bone masses increases until you're about 30 both in men and women and then slowly declines with age, about one per cent per year. So if density was a certain amount of difference from this young normal, a woman then was labelled as having osteoporosis.

In that study group, there was financial support from three pharmaceutical companies and we have to remember that bones, like hair and skin, get thinner with age. The new definition meant that about one in five women over the age of 50 years old would be classified as having osteoporosis. So just let's have a look at why that is.

If we draw a box like this, the occasional woman at the age of 30 might have osteoporosis, but many women, once they got into their 60s, 70s and 80s, would be defined by this somewhat arbitrary definition the WHO invented to have osteoporosis before there was any fracture.

So, a question arises, is osteoporosis a disease or a risk factor? The symptomatic thing is the fracture, whereas there are a number of predictors for getting that fracture. So while it's commonly described as a disease, some doctors don't agree with this because the way it's defined means that many otherwise healthy women are being automatically labelled as having this condition. Some researchers prefer to describe low bone mineral density prior to the fracture as a risk factor, a factor that increases the risk of having a broken bone, rather than calling it a disease before the broken bone occurs.

[Section 2]

Paul Glasziou: Hello, it's Paul Glasziou back again. I want to now talk to you about, apart from bone density which we talked about last time, what else increases the risk of fracture. Okay, so what else increases risk? So bone density which we talked about is only one small part of your overall risk of fractures and some researchers believe it's a pretty poor predictor of fracturing in an individual. So we'll discuss medications as one way of improving bone density and reducing fractures, but there are other ways to try and reduce falls and fractures, for example, certain kinds of exercise improving bone strength and balance, though the evidence supporting these strategies is not as strong as the medications.

So what else increases risk? Well, age is a big factor. Most hip fractures happen in those people who are 80 or older. Younger

women generally have a very low risk of fractures, particularly hip fracture. For a 50-year-old, the risk of a hip fracture is about one in 5000 per year, whereas for an 80-year-old, the risk is almost two in 100 per year, or almost one in 10 over five years.

So what else does increase the risk? The bone mineral density, which defines osteoporosis, is only one of the risk factors that increases your chance of a fracture. So in the picture here, the person is falling, so what's your risk of falling, but also the way that you fall as well and the cushioning when you fall. So a list of those factors includes your age, previous fractures, family history of fracture, use of alcohol, smoking, some other medicines, particularly those that make you prone to falling, being physically inactive and having a fall. So many people who have fractures don't have low bone mineral density, that is, they don't have osteoporosis; it's all those other factors that can come into play on whether you have a fracture or not.

You can calculate the total risk from all of those things with one of these sorts of tools that many folk have developed to calculate your risk, which basically includes all of those factors. You can see the bone mineral density is just one; it's number 12 here.

[Section 3]

Paul Glasziou: Okay, it's Paul Glasziou again and in part three we're going to talk about how well do common medications work, specifically for osteoporosis. So for a little while we're going to forget all of those other risk factors and just concentrate on osteoporosis. So how well do the medications work? We'll focus on bisphosphonate medications, including Fosamax, which some of you might have heard of, because they're the most common treatments for osteoporosis. They can lower the risk of fracture and we're going to focus on this medicine for the presentation.

So, we're going to present two different sets of evidence; first, evidence with women who mostly haven't had a fracture before and secondly, evidence with women who mostly had a previous fracture, because the risk is different in those two groups.

Okay, before we start though, let me just explain the diagrams that we are going to use. Instead of drawing pictures of women, we're just going to use dots to represent one woman each, so you'll see a lot of figures like this.

So, let's go back to that first piece of evidence which is women who haven't had a fracture before. Okay, so let's imagine 100 women and what's the impact of the medication, the bisphosphonate or Fosamax, on hip fracture in women without a previous fracture? So first of all, if 100 women do not take medicine for about two years, one woman will have a fracture. That's the one in 100 here. Okay, now if 100 women took the medicine for about two years, one will have a fracture. That's the one woman here in 100. Okay, so, no hip fractures are prevented by the medicine. That is, in this group of women, nobody gets a benefit for hip fracture.

But let's look at spinal fracture. Again, we're talking about women who haven't had a previous fracture and in the 100 women who don’t take the medicines, the control group, three women will have a spinal fracture, those ones there. But of the 100 women who take the bisphosphonate, the Fosamax, for two years, only one will have a fracture. So this time it's made a difference to spinal fracture, that is, two spinal fractures are prevented by the medicine. But 98 of the women taking the medication do not get that benefit; most of them are the green dots here.

So, let's move on to the second one we said, the evidence in women who have had a previous fracture and who are therefore, as we said earlier, at a higher risk of a fracture. Same sorts of diagrams again, in 100 women who did not take the medicine, two would get a hip fracture and 100 women who take the medicines, one would get a hip fracture. So in this case, in women with a previous fracture, we've now made a difference. One hip fracture is prevented over the two years by the medicine, but again, 99 of the women taking the medication won't get this benefit.

So, how well do the medicines work? The benefits of medications are very different for women in different age groups. We've seen

that the presence of a previous hip fracture makes a difference, but if you remember back, it's just - sorry, previous fracture - but if you remember back, it's just one of the many risk factors. We're just going to focus now on the influence of age because it's the biggest risk factor.

So, let's look at women aged 60 to 65 with a previous fracture; 100 women take the medicine for about five years, one woman will have a fracture prevented, the other 99 will not get benefit. Okay, in women aged 85 to 89, older, taking the medication for five years, three women will have a fracture prevented this time. So the difference here is that the older women are more at risk of a fracture and therefore giving them the medication has a greater benefit and this is for non-spinal fractures generally.

Okay, so let's look at the medications' impact on non-spinal fractures. If 100 women who are at low overall risk take the medicine for about five years, two women will have a non-spinal fracture prevented. Of course the other 98 taking the medicine won't get this benefit over the five years. Okay, but if we take 100 women who are at high overall risk taking the medicine, again for about five years, six women will have a non-spinal fracture prevented and the other 94 of course won't get the benefit.

Okay, in considering the decision to take any medication though, we always have to weigh up the benefits and harms. So what harm might come from these medications? So the side effects of these bisphosphonates may include some upper gastrointestinal injury, that is, your stomach; things like nausea, indigestion, vomiting, heartburn may happen to about one in 10 women but there's some uncertainty in that estimate.

Then there are some extremely rare but more important side effects which may be an unusual fracture of the thigh bone or femur can occur, because of an unusual effect. There's also the dead jaw, this so-called osteonecrosis of the jaw, which happens to about one in 10,000 women, particularly if they're having dental treatment and they continue on their bisphosphonates. Again, there is some uncertainty within that estimate.

[Section 4]

Paul Glasziou: Hi, it's Paul Glasziou again for our final part which is to talk about osteoporosis as now defined by bone mineral density and overdiagnosis. So, being overdiagnosed, what does that mean? It means being diagnosed with a disease that would never cause you harm but has the potential to cause harm through being over-treated or just simply being labelled with a condition and you then worry about it.

Okay, so for example, being diagnosed with a painless slipped disc which would never cause problems. If you do a back x-ray sometimes you can find these and people who have no symptoms at all can just worry that it was there. So overdiagnosis is being recognised as a growing problem across many conditions where we're finding things, from imaging, for example, or through definitions, that would never harm people and can cause them overtreatment and concerns.

Okay, so let's specifically look at overdiagnosis and osteoporosis. Women are diagnosed and treated for osteoporosis to try and prevent fractures. Fractures are the thing that we're really worried about, not just having the thin bones. As a consequence, some women are diagnosed and treated who would never have a fracture, the thing that we're really worried about. This is similar to other conditions, sometimes being diagnosed with high blood pressure, you'd never have a heart attack or a stroke, but people take the medication to prevent that. It's not clear what proportion of possibly many women diagnosed would never have had a fracture anyway, so we can think of them as being overdiagnosed.

So some researchers and doctors have been concerned that the 1994 definition that we went through earlier, which was even recognised at the time as being somewhat arbitrary, means that many women automatically labelled with osteoporosis are overdiagnosed. This means while some women benefit from a diagnosis and treatment, other women are experiencing the side effects, hassle, the cost of treatment, but without getting the benefit. There's also the psychological impact of the label with the

potential anxiety about falls and its impacts on activity that can be a harm as well as the side effects of the medications and overtreatment.

End of transcript
